# Late Relapse and Reinfection in HCV Patients Treated with Direct-Acting Antiviral (DAA) Drugs

**DOI:** 10.3390/v13061151

**Published:** 2021-06-16

**Authors:** Claudia Minosse, Cesare E. M. Gruber, Martina Rueca, Chiara Taibi, Mauro Zaccarelli, Elisabetta Grilli, Marzia Montalbano, Maria R. Capobianchi, Andrea Antinori, Gianpiero D’Offizi, Fiona McPhee, Anna Rosa Garbuglia

**Affiliations:** 1 Virology Unit, National Institute for Infectious Diseases, INMI Lazzaro Spallanzani IRCCS, Via Portuense 292, 00149 Rome, Italy; claudia.minosse@inmi.it (C.M.); cesare.gruber@inmi.it (C.E.M.G.); martina.rueca@inmi.it (M.R.); maria.capobianchi@inmi.it (M.R.C.); argarbuglia@iol.it (A.R.G.); 2 Infectious Disease—Clinical Department, National Institute for Infectious Diseases, INMI Lazzaro Spallanzani IRCCS, Via Portuense 292, 00149 Rome, Italy; mauro.zaccarelli@inmi.it (M.Z.); elisabetta.grilli@inmi.it (E.G.); marzia.moltalbano@inmi.it (M.M.); andrea.antinori@inmi.it (A.A.); gianpiero.doffizi@inmi.it (G.D.); 3 Bristol-Myers Squibb Research and Development, Cambridge, MA 02142, USA; fiona.mcphee@bms.com

**Keywords:** hepatitis C virus, HCV, HCV reinfection, HCV late relapse, next-generation sequencing, NGS, phylogenetic

## Abstract

The risk of hepatitis C virus (HCV) recurrence after direct-acting antiviral (DAA) treatment is <0.5%. However, the distinction between HCV RNA late relapse and reinfection still represents a challenge in virological diagnostics. The aim of this study was to employ next-generation sequencing (NGS) to investigate HCV RNA recurrence in patients achieving a sustained virologic response (SVR) at least six months post-treatment. NGS was performed on plasma samples from six HCV-positive patients (Pt1–6) treated with DAA. NGS of HCV NS5B was analyzed before treatment (T0), after HCV RNA rebound (T1), and, for Pt3, after a second rebound (T2). Reinfection was confirmed for Pt5, and for the first rebound observed in Pt3. Conversely, viral relapse was observed when comparing T0 and T1 for Pt6 and T1 and T2 for Pt3. Z-scores were calculated and used to predict whether HCV-positive patient samples at different time points belonged to the same quasispecies population. A low *Z*-score of <2.58 confirmed that viral quasispecies detected at T0 and T1 were closely related for both Pt1 and Pt2, while the Z-score for Pt4 was suggestive of possible reinfection. NGS data analyses indicate that the Z-score may be a useful parameter for distinguishing late relapse from reinfection.

## 1. Introduction

Hepatitis C virus (HCV) infection is a major cause of chronic liver disease, with approximately 71 million chronically infected individuals worldwide [1,2,3].

Several phases have occurred in the history of antiviral therapy for the treatment of chronic HCV hepatitis. The first drug used in the treatment was interferon (IFN) with sustained virologic response (SVR) rates of less than 10%. In the late 1990s, ribavirin was combined with IFN, resulting in SVR rates of 35–40% in treated patients. An important turning point came with the introduction of pegylated interferon (peg-IFN) where SVR rates greater than 50% were achieved. The introduction of new direct-acting antivirals (DAAs) revolutionized HCV treatment, leading to the possibility of HCV elimination [4]. In fact, over 90% of all HCV genotypes respond to successful DAA treatment with minimal to moderate adverse effects [5]. The endpoint for HCV therapy is the achievement of SVR. SVR is defined as undetectable HCV RNA in serum or plasma 12 weeks (SVR12) or 24 weeks (SVR24) after the end of therapy, as determined by a sensitive molecular method with a lower limit of detection of ≤15 IU/mL [6].

HCV recurrence (both reinfection and late relapse) is defined as the reappearance of HCV RNA in treated patients after achieving SVR. Reinfection is suspected in cases of a recurrence of HCV infection after more than 12 or 24 weeks post-SVR [6]. The risk of recurrence after achieving an SVR is <0.5% and is related to reinfection or late relapse [7]. However, in special populations, reinfection could be confused with late relapse. In fact, reinfection events have been described mostly in high-risk patients such as drug users and patients coinfected with human immunodeficiency virus (HIV) [8,9,10,11]. The risk of HCV reinfection is higher in people who inject drugs (PWID) and share injection equipment. Additionally, reinfection is higher in men who have sex with men (MSM) and participate in risky sexual practices and chemsex activities, defined as the use of drugs to enhance sexual experience [11,12,13]. 

An unresolved question is how long the follow-up time should be after the end of treatment (EOT) before a DAA-treated patient is considered cured of HCV. The international guidelines indicate that 12 weeks after stopping therapy is sufficient. Moreover, Sarrazin et al. [7] demonstrated that of 12 patients who achieved SVR and subsequently had late recurrent viremia after treatment with a sofosbuvir (SOF)-based regimen, seven became reinfected with a different HCV strain post-treatment, while the remaining five patients had a virological relapse with the same strain. In most studies, Sanger sequencing analysis was performed to differentiate late relapse from reinfection, albeit with poor resolution [7,14]. Nevertheless, reinfection can be confirmed when the infection is caused by a different genotype or by a phylogenetically distant strain. Conversely, next-generation sequencing (NGS) can detect thousands of viral sequences in a single sample analysis, providing better insight into mutant spectra in viral populations [15]. Currently, NGS is widely used to investigate the emergence of HCV antiviral resistance [16], as well as for reconstructing HCV transmission networks [17].

NGS can be a useful tool to detect the degree of similarity of viral strains between and within individuals. To our knowledge, few studies have employed NGS to distinguish between late relapse and HCV reinfection, especially for PWIDs and transmission networks between individuals with high-risk behaviors [18].

The aims of the present study were to perform virological characterization of HCV-positive patient-derived samples by NGS to distinguish between late relapse and reinfection in DAA-treated patients with HCV RNA recurrence after achieving SVR24.

## 2. Materials and Methods

### 2.1. Patients 

The institutional ethics committee at the INMI L Spallanzani Hospital approved the protocol for the described study (Liver study and antiviral treatments, reference approval code 55/08, 29 October 2008), which was conducted in accordance with Good Clinical Practice, as defined by the principles of the Declaration of Helsinki. All patients provided written informed consent to be included in the study.

Plasma samples from six HCV-infected patients (Pt1–Pt6) treated with different DAA regimens at the INMI L Spallanzani Hospital and who presented with late viral rebound were considered for NGS analysis.

**Figure 1:**
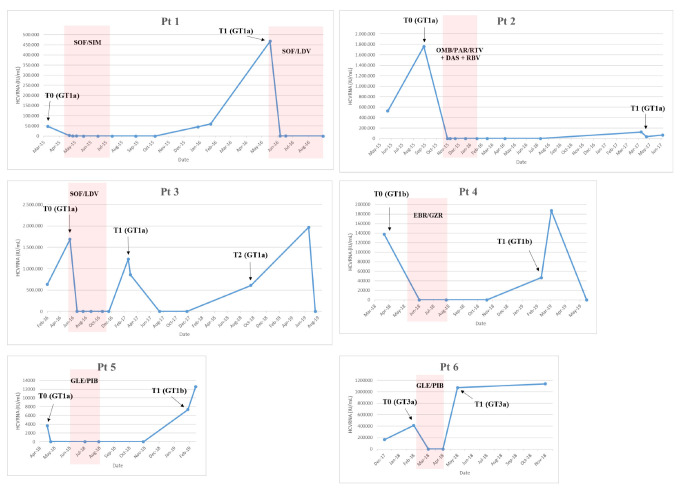
Patient HCV RNA viral load over time. The sampling times T0, T1, and T2 are indicated. The period and type of treatment are indicated in the red box. DAS: Dasabuvir; EBR: Elbasvir; GLE: Glecaprevir; GZR: Grazoprevir; OMB: Ombitasvir; PAR: Paritaprevir; PIB: Pibrentasvir; RBV Ribavirina; RTV: Ritonavir; SIM: Simeprevir; SOF: Sofosbuvir.

The first patient (Pt1, Figure 1) was a 54-year-old male with a history of drug addiction since 1982 to 1984, coinfected with HIV and HCV genotype (GT)1a, cirrhosis (Child Pugh A), and F4 fibrosis (stiffness of 15.7 kPa). The patient was treated with sofosbuvir/simeprevir from April 2015 to July 2015 and became HCV GT1a-positive in December 2015. On 22 January 2016, the HCV-resistance-associated substitution (RAS) R155K emerged in the NS3 protease (NS3-R155K). The patient was retreated with sofosbuvir/ledipasvir from May 2016 to November 2016.

The second patient (Pt2, Figure 1) was a 58-year-old male infected with HCV GT1a and had F2 fibrosis and a non-Hodgkin Lymphoma in remission. The patient was treated with ombitasvir/paritaprevir/ritonavir + dasabuvir/ribavirin for 12 weeks from 26 October 2015 to 19 January 2016 and achieved SVR24. After one year, the patient became HCV GT1a-positive. On 5 June 2017, HCV RAS Q30R in NS5A was detected.

Pt3 (Figure 1) was a 49-year-old male, active PWID, coinfected with HIV and HCV GT1a, and cirrhotic. The patient presented with compensated cirrhosis (MELD 8 and Child-Pugh score A5) and a Fibroscan value of 31 Kpa. The patient was treated with sofosbuvir/lepidasvir for 24 weeks from May to November 2016 but was shown to have an apparent relapse at post-treatment Week 12 (T1, Figure 1). After 2 months, spontaneous viral clearance was observed. The patient remained HCV-negative for an additional 3 months and returned to our center one year later and was HCV GT1a-positive (Pt3_T2 in Figure 1). No reported HCV RAS were detected (18 February 2019).

Pt4 (Figure 1) was a 51-year-old female infected with HCV GT1b and F1 fibrosis. The patient was treated from 6 May 2018 to 28 July 2018 with elbasvir/grazoprevir and achieved SVR12 but relapsed at 24 weeks post-treatment. The patient was retreated with sofosbuvir/velpatasvir/voxilaprevir from April 2019 to July 2019. HCV RASs were detected in NS5A: NS5A-L31V and -Y93H (2 March 2019: T1). Some of the patient’s relatives (sister and mother) were also HCV-positive.

Pt5 (Figure 1) was a 43-year-old male infected with HCV GT1a and F2 fibrosis. The patient was treated with glecaprevir/pibrentasvir for 8 weeks from 6 June 2018 to 30 July 2018. At EOT, the patient admitted to sexual exposure with an HCV-positive individual. At 12 weeks post-treatment, the patient presented with low-level viremia and at 24 weeks post-treatment was HCV GT1b-positive (Pt5_T1, Figure 1). At this time, no reported HCV RAS were detected.

Pt6 (Figure 1) was a 59-year-old male, former drug user, coinfected with HIV and HCV GT 3a with F2 fibrosis (Fibroscan value was 7.2 kPa) and treatment-naïve. The patient was treated with glecaprevir/pibrentasvir for 8 weeks from 22 February 2018, and although rapid HCV RNA declines were observed during treatment, HCV RNA quickly rebounded at EOT. The patient was never re-treated. The HCV NS5A-Y93H RAS emerged (22 November 2018). From clinical records, the patient appeared to be adherent to therapy, although HCV RNA levels never became undetectable; HCV < 25 IU/mL was achieved after two months of treatment.

### 2.2. Virological Determination 

HCV RNA levels were assessed using the HCV Abbott RealTime HCV Assay with a lower limit of detection (LLOD) of 12 IU/mL (Abbott Molecular System, RealTime™ HCV, Abbott Molecular Inc, Des Plaines, IL, USA).

HCV genotype was determined using the Real-time HCV genotype II assay (Abbott Molecular, Abbott Molecular Inc, Des Plaines, IL, USA).

### 2.3. HCV RNA Extraction, Amplification, and HCV RAS Determination 

Viral RNA was extracted from plasma using the QIASYMPHONY DSP virus/Pathogen kit with an automated Nucleic acid QIASYMPHONY instrument (Qiagen, Hilden, Germany). 

For HCV RAS analyses, the NS3 region was amplified using nested PCR using primers specific for GT1a or GT1b, as previously described [19,20]. Specific GT3a primers were designed with the following sequences: NS3_3a-for 5′-CCGATCACAGCATAYRCCC-3′ (3398–3416 nt in X76918.1) and NS3_3a_rev1 5′-CTTRCCRTAGGTGGARTAGG-3′ (4210–4191 nt in X76918.1) for first-round PCR and NS3_3a_rev2 5′- GGATGTTGGGGTCRATCCC-3’(4151-4133 nt in X76918.1) for second-round PCR. The reverse transcription and first-round PCR were conducted using ONEStep RT-PCR Kit (Qiagen); the nested PCR was performed using GoldTaq DNA Polymerase (Applied Biosystems, Foster City, CA, USA). 

The HCV NS5A region was amplified using nested PCR and pan-genotypic primers, as previously described [21].

The HCV NS5B region was amplified using nested PCR and pan-genotypic primers, as previously described [22]. To obtain a fragment of <600 bp for NGS analyses, a new inner forward primer was used for second-round PCR: 5′-TGGGIGTKCGYGTITGYGAGAA-3′ (8095–8116 nt in AF009606). The reverse transcription and first-round PCR were conducted using the ONEStep RT-PCR Kit (Qiagen); the second-round PCR was conducted using FastStart Taq DNA Polymerase (Roche, Molecular Systems, Pleasanton, CA USA). Positive and negative controls, which contained standardized viral RNA extracts and nuclease-free water, respectively, were included in each RT-PCR assay.

Resultant HCV amplicons (603 bp, 600 bp, 754 bp, 637 bp, and 543 bp for NS3 GT1a, GT1b and GT3a amplicons, NS5A, and NS5B, respectively) were sequenced directly using Prism BigDye in an ABI3100 DNA automated Sequencer (Applied Biosystems, Forster City, CA, USA).

HCV NS3, NS5A, and NS5B sequences were submitted to Geno2pheno [HCV] v. 0.92 [23] systems for resistance analyses.

### 2.4. Phylogenetic Sanger Analysis

Each patient-derived Sanger sequence was aligned, using CLUSTAL W program, with reference sequences (GT1a: EF407457.1; GT1b: EU781827.1; GT3a: X76918.1), available from GenBank. The best fit model was identified (considering The Bayesian information criterion (BIC) and the Akaike information criterion (AIC)) and a phylogenetic analysis performed using the maximum likelihood method with Kimura 2-parameter model+G, implemented in MEGAX software [24]. To evaluate the robustness, bootstrap probabilities were estimated using 500 replications.

### 2.5. Next-Generation Sequencing (NGS) Analysis

NGS was employed to analyze the HCV NS5B region from samples derived from six patients (Pt1–Pt6) collected before treatment (T0) and after HCV RNA rebound (T1). For Pt3, sequencing was also performed on a sample obtained after the second rebound (T2). 

The Ion-Torrent S5 NGS platform was used to analyze 13 samples collected from six patients. After purification of amplicons by AMPure XP beads (Beckman Coulter, Chaska, MN, USA) and quantitation with high-sensitivity dsDNA assay on Qubit 2.0 fluorometer (ThermoFisher Scientific, Waltham, MA, USA), libraries were prepared using 100 ng of amplified DNA using the Ion Xpress Plus Fragment Library kit (ThermoFisher Scientific, US. Waltham, Massachusetts) following manufacturer’s instructions. Resultant libraries were quantified on the Bioanalyzer 2100 (Agilent Technologies, Santa Clara, CA, USA) using a high-sensitivity kit and pooled to obtain a concentration of 100 pM.

For each sample, reads of less than 500 nt in length and with low mean quality (Phred < 20) were discarded. Pollux v.1.0.2 [25,26] was adopted to correct for erroneous substitutions. Forward-oriented reads were separated from reverse-oriented reads, and insertions and deletions were manually adjusted after global alignment with respect to the corresponding Sanger sequence using MUSCLE software v3.8.31 [27]. Corrected reads were clustered at 100% using CD-HIT v.4.6 [28]. Sequences represented by a cluster of greater than four identical reads and with at least one forward-oriented and one reverse-oriented read were retained for further analysis.

### 2.6. NGS Phylogenetic Analysis 

Maximum likelihood phylogenetic analyses were performed using IQ-TREE [29]: transition model with empirical base frequencies and Free Rate heterogeneity were selected with ModelFinder, and the best-fitted tree was established by performing 1000 bootstrap ultrafast replicates. The variants were identified by clustering all NGS reads from each sample, the Sanger sequences from each sample, and by adding the reference sequences for GT1a (EF407457.1), GT1b (EU781827.1), GT3a (X76918.1), and GT4d (DQ418786.1) to the NGS phylogenetic tree. The GT4d reference sequence was selected as an out-group.

For each patient, the genetic distance between and within each sample was calculated, and intra-sample nucleotide diversity (π) was estimated as the mean genetic distance within each sample [30,31].

### 2.7. Statistical Analysis 

The Mann–Whitney U test [32] was adopted to determine whether two samples at T0 and T1 from the same patient belonged to the same viral population. 

To calculate the Kimura-2-parameter distance and to plot in three-dimensional scale the genetic distances of every representative sequence of the quasispecies present in each sample with reference sequences denoting GT1a (EF407457.1), GT1b (EU781827.1), and GT3a (X76918.1), the “scatterplot3d” package was employed using home-made R script. 

With the assumption that distances between quasispecies and reference sequences follow a normal distribution, Z-scores were calculated for each patient to test the hypothesis that HCV sequences at different times belonged to the same quasispecies population. In particular, distance Z_GT1a_, Z_GT1b,_ and Z_GT3a_ scores were calculated for each patient with respect to reference sequences using the following equation:

$$Z=\;\frac{{\bar{X}}_{T1}-{\bar{X}}_{T0}}{\sqrt{{\left(\Delta X_{T1}\right)}^{2}+\;{\left(\Delta X_{T0}\right)}^{2}}},$$

with ${\bar{X}}_{T0}$, ${\bar{X}}_{T1}$, $\Delta X_{T0}$, and $\Delta X_{T1}$, respectively, representing the mean and standard deviation distances between quasispecies and reference sequences at T0 and T1 [33]. For *Z* > 2.58, it possible to consider T1 and T2 as two different quasispecies populations with a significance level α = 0.01.

Frequencies of intra-sample Single Nucleotide Variants (iSNV) and intra-sample Single Aminoacidic Variants (iSAV) were collected for each sample by using a home-made python script.

## 3. Results

### 3.1. NGS Analysis of Sequence Reads 

NGS analyses of the HCV NS5B region were performed on 13 samples from six patients (Pt1–Pt6) collected at baseline (before treatment, T0) and at the first available plasma sample with HCV RNA recurrence. Pt3 experienced a second rebound in HCV RNA, and thus the corresponding sample (T2) was included in the analysis. High-throughput sequence data were deposited in the NCBI’s Sequence Read Archive (SRA) and are accessible under the Project Number [PRJNA720653].

The mean number of raw reads obtained for each sample evaluated by NGS was 220,106 (SD = 146,610). Table 1 summarizes the number of raw reads and the number of reads obtained after quality filtering and clustering. The mean number of clusters, represented by at least five high-quality reads, was 117 (SD = ±91).

### 3.2. NGS Phylogenetic Analysis 

**Figure 2:**
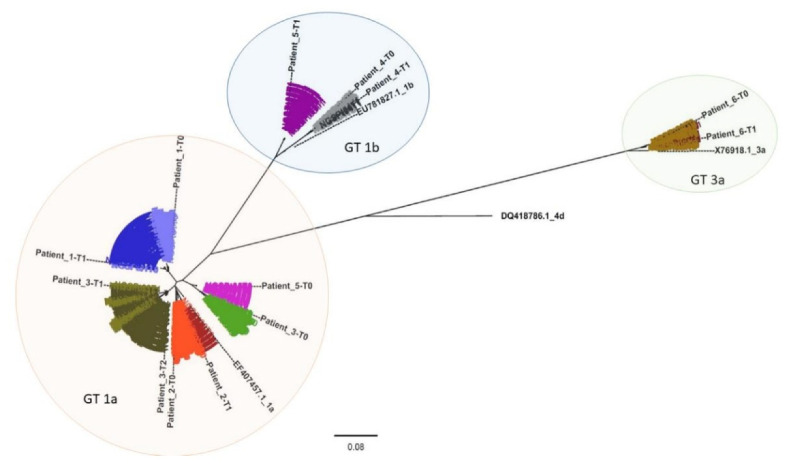
HCV NS5B phylogenetic tree. The maximum-likelihood method with the transition-free rate model and empirical codon frequencies was employed to build the NS5B phylogenetic tree. The HCV GT4d sequence (DQ418786.1) was selected as an out-group. Reference sequences (GT1a: EF407457.1; GT1b: EU781827.1; GT3a: X76918.1) were retrieved from GenBank. Sanger sequences for all patient samples were included and indicated as Patient_N-T0, Patient_N-T1, and Patient_N-T2. The bar represents the genetic distance (substitution per nucleotide position). Bootstrap analysis with 1000 replicates was performed. Each patient is represented by a different color: Pt1_T0 and Pt1_T1 are depicted in light and dark blue, respectively; Pt2_T0 and Pt2_T1 are depicted in light and dark red, respectively; Pt3_T0, Pt3_T1, and Pt3_T2 are depicted in light green, olive green, and dark olive green, respectively; Pt4_T0 and Pt4_T1 are depicted in light and dark grey, respectively; Pt5_T0 and Pt5_T1 are depicted in light and dark violet, respectively; Pt6_T0 and Pt6_T1 are depicted in light and dark brown, respectively. The orange, blue, and green circled areas represent sequences clustering with GT1a, GT1b, and GT3a, respectively.

A phylogenetic analysis was performed to compare all clustered sequencing reads representing each patient sample (Figure 2 and Figures S1–S6). Patient-derived HCV NS5B Sanger sequences (indicated in Figure 2 as Patient_N-T0, Patient_N-T1, and Patient_N-T2) were also included, and, as expected, they grouped with the corresponding clustered sequences. From the phylogenetic tree, three clusters corresponding to three distinct genotypes resulted: GT1a, GT1b, and GT3a (orange, blue, and green areas, respectively, in Figure 2). 

Each analyzed time-point for Pt1 (light and dark blue sequences) and Pt2 (light and dark red), respectively, displayed a similar sequence distribution in GT1a. This was also apparent for Pt4 (light and dark grey) and Pt6 (light and dark brown) in GT1b and GT3a, respectively. However, Pt6 appeared to have two distinct variant species in both Pt6_T0 (represented by 328 and 25 reads, respectively) and in Pt6_T1 (represented by 721 and 5 reads, respectively).

All observed Pt3 variants (green) clustered with GT1a. Interestingly, while Pt3 T0 sequences (light green) formed a cluster unrelated to sequences detected in T1 and T2 (olive green and dark olive green, respectively), T1 and T2 variants appeared to be phylogenetically related and associated with the same independent cluster. This was exemplified by three variants detected in Pt3_T1 (represented by 51, 24, and 13 reads, respectively) that were also detected in Pt3_T2 (represented by 11, 5752, and 14 reads, respectively).

Based on Sanger sequencing results, Pt5 (violet) appeared to experience reinfection with HCV as sequences from T0 (light violet) and T1 (dark violet) clustered with GT1a and GT1b, respectively. NGS phylogenetic analysis supported the Sanger sequencing results since no GT1b variants were detected in the T0 sequence reads. This hypothesis was also supported by the patient’s declared risky lifestyle. 

Moreover, phylogenetic analysis based on Sanger sequences confirmed NGS results related to Pt3 and Pt5 (Figure S7).

### 3.3. NGS Diversity Analysis 

By using phylogenetic analysis, it was confirmed that Pt5 was reinfected with a different genotype while Pt3 was reinfected with a distantly related GT1a strain (between T0 and T1). In contrast, the same variant was detected in T1 and T2 samples for Pt3, and T0 and T1 samples for Pt6, supporting the hypothesis that these patients experienced a late viral relapse.

To define a distance threshold able to discriminate between late relapse and reinfection events, the genetic distances of clustered sequences between and within each patient sample were calculated. The mean genetic distance within each sample is reported in Table 2 as diversity (π). To verify if variants present in T0 and T1 belonged to the same distribution, we compared the nucleotide distances between T0 and T1 (and for Pt3, nucleotide distances between T1 and T2 were also included) with genetic distances within each sample. The distributions, based on these distances, were significantly different with a *p*-value <0.001 for all samples (Table 2). Thus, no conclusions could be derived when using this approach.

**Figure 3:**
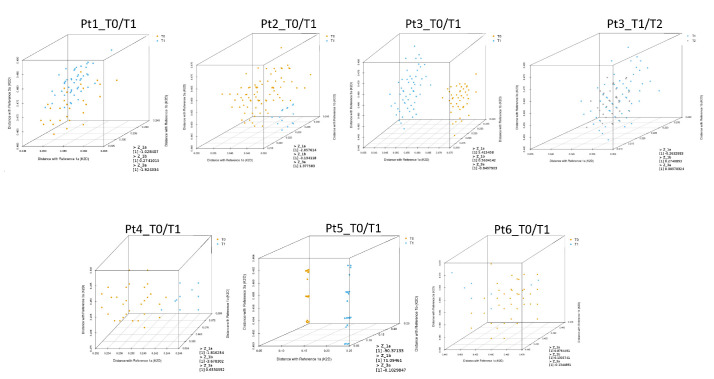
Three-dimensional plots illustrating genetic distances of quasispecies sequences with reference sequences representing GT1a (EF407457.1), GT1b (EU781827.1), and GT3a (X76918.1). Genetic distances between quasispecies sequences at T0, T1, and T2 are shown in orange, blue, and grey, respectively.

The next step was to calculate the genetic distances of every representative sequence detected in the quasispecies in each sample, compared with the reference sequences representing GT1a (EF407457.1), GT1b (EU781827.1), and GT3a (X76918.1). These distances were mapped and displayed using a three-dimensional plot to better visualize the distribution of quasispecies at different time points (Figure 3).

Z-scores were also calculated for each patient sample with respect to reference sequences representing GT1a (EF407457.1), GT1b (EU781827.1), and GT3a (X76918.1). Z-scores were calculated to test the hypothesis that HCV sequences at different time points belonged to the same quasispecies population, as indicated in the Materials and Methods section. Z_GT1a_, Z_GT1b,_ and Z_GT3a_ scores are reported in Table 3.

The T0 and T1 quasispecies detected in Pt3 and Pt5 samples were clearly separated in the three-dimensional plots (Figure 3). As described above, T0 and T1 sequences for Pt5 clustered with GT1a and GT1b, respectively, suggesting probable reinfection. In this patient, Z_GT1a_ and Z_GT1b_ scores were −90.37 and 71.09, respectively (Table 3). Phylogenetic analysis of T0 and T1 sequences for Pt3 were also indicative of reinfection. The Z_GT1a_ score for this patient was 5.42.

For Pt6 and Pt3, sequence analyses between T0 and T1 and between T1 and T2, respectively, indicated viral rebound. In both cases, Z_GT1a_, Z_GT1b,_ and Z_GT3a_ scores were less than 1, and genetic distances were interspersed in the three-dimensional plots without any clear separation between viral populations detected at the reported time-points.

For Pt1, Pt2, and Pt4, it was ambiguous whether reinfection or late relapse had occurred when evaluating samples by phylogenetic analysis. However, low Z-score values (Z_GT1a_, Z_GT1b,_ and Z_GT3a_ scores < 2.58) suggested viral quasispecies detected at T0 and T1 were closely related for both Pt1 and Pt2. Genetic distances of the quasispecies detected in Pt1 samples appeared interspersed in the three-dimensional plot, while genetic distances of quasispecies detected in Pt2 samples were only partially separated (Figure 3). In contrast, the Z_GT1b_ value for Pt4 was suggestive of reinfection.

### 3.4. Nucleotide and Amino Acid Frequency Analysis in Quasispecies

**Figure 4:**
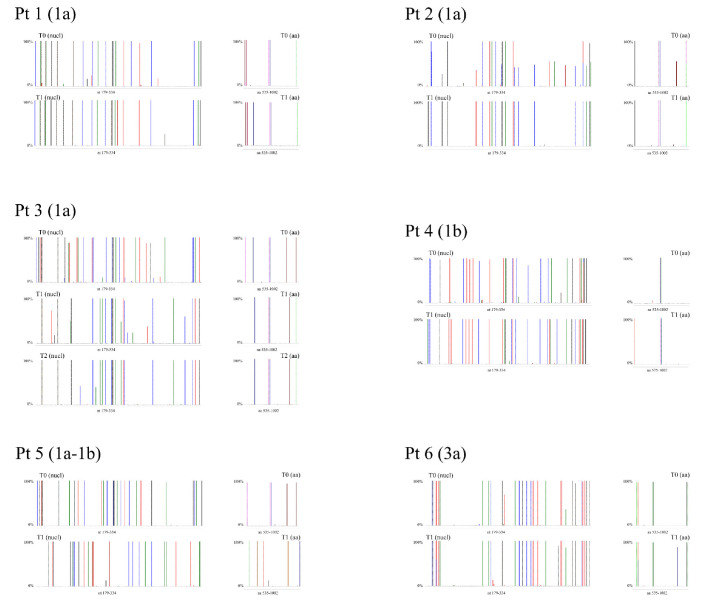
Graphic representing the frequency of nucleotide and amino acid substitutions detected at each tested time point for the six patients (Pt1–Pt6).

Figure 4 depicts the frequency of HCV NS5B substitutions and emergent DAA RASs detected in patient samples compared with respective genotype reference sequences. The details of these substitutions and emergent DAA RASs and their frequencies are reported in Tables S1–S6.

In Pt1, sequence analysis of samples collected at time-points T0 and T1 revealed a difference in substitutions at one amino acid (aa) position: a glutamine (Q) at NS5B aa 206 was detected in all (100%) quasispecies sequences at T0, which was replaced with either an arginine (R) (CGA; representing 99.90% of quasiespecies sequences) or a stop codon (TGA; 0.10% of sequences) (Table S1) at T1. It is feasible that NS5B-Q206R may be linked to the selection of the NS3-R155K RAS, although further sequence analyses of sofosbuvir/simeprevir failures would be required for confirmation.

In Pt2, NS5B sequence analysis revealed substitution enrichment at two aa positions when comparing quasispecies detected at T0 and T1: at aa 300, Q300 (CAA; 55.01%) or R300 (CGA; 44.99%) was detected in T0 sequences, while only R300 (CGA) was detected at T1. At aa 327, either alanine (A) (GCG; 50.12%) or tyrosine (Y) (GTG; 48.70%) was detected in T0 sequences, while only A327 was detected at T1 (Table S2). However, the linkage of A327 with emergent RAS could not be confirmed.

In Pt3, differences in NS5B sequences were detected at five aa positions between T0 and T1, while no substantial differences were detected between the T1 and T2 time-points (Table S3). In particular, valine (V)184 (99.78% GTG) and A184 (0.22% GCG) were detected at T0, whereas only leucine (L) (CTG) was detected at T1 and T2. At aa position 212, lysine (K) (99.78% AAG) and threonine (T) (0.22% ACG) were detected at T0, while only R212 (AGG) was detected at T1 and T2. At aa position 300, Q (CAA) was detected at T0, while R300 was detected at the T1 and T2 time-points (100% CGA at T1; 99.96% CGA and 0.04% CGG at T2). At aa position 309, only R (CGG) was detected at T0, Q300 (CAG) was detected at T1, while Q300 (99.84% CAG) and R300 (0.16% CGG) were both detected at T2. At aa position 327, only Q (CAG) was detected at T0, A327 (GCG) was detected at T1, while A327 (99.93% GCG), T327 (0.03% ACG), and V327 (0.04% GTG) were detected at T2. In this patient, aa substitutions detected between the T1 and T2 sequences were suggestive of a high similarity in viral quasispecies, and when compared with the T0 quasispecies sequences, these differences were not related to the appearance of DAA RASs. As hypothesized from the phylogenetic analysis, differences in the detected aa substitutions between T0 and T1 were indicative of reinfection after the patient achieved the first SVR and viral rebound at T2 after an apparent spontaneous clearance (Table S3). 

In Pt4, emergence of a single NS5B substitution was detected at T1 when compared with NS5B sequences at T0: T181 (ACC) was detected at T0, while an asparagine (N) (AAC) was detected at T1 (Supplementary Data Table S4).

For Pt5, NS5B sequence analysis revealed twenty aa differences between the T0 and T1 time-points. This was expected, since GT1a sequences were detected at T0 and only GT1b sequences were detected at T1 (Supplementary Data Table S5). 

In Pt6, K304 (AGC) was the predominant species (99.71%) at T0, while R304 (AAG) was a minor variant (0.29%). This was reversed at T1: R304 was enriched (89.24% of quasispecies sequences) compared with K304 (10.76%) (Table S6).

## 4. Discussion

In this study, virological characterization of HCV-positive patient-derived samples by NGS was performed to discriminate between late relapse and reinfection in six DAA-treated patients experiencing late HCV RNA recurrence after achieving SVR24. Phylogenetic analysis of Sanger sequences supported the hypothesis that the observed viral rebounds in Pt3 (between T0 and T1) and Pt5 (between T0 and T1) were reinfection events (Figure S7). The behavioral risks of these patients also supported the idea of reinfection events: Pt3 was coinfected with HIV, while Pt5 admitted to a risky sexual encounter and both patients were PWID. These findings are in agreement with a study showing that individuals at risk of coinfection or PWID were susceptible to reinfection [34]. For the other patients in our study, late relapse and reinfection could not be resolved by phylogenetic analyses of Sanger sequences (Figure S1). 

The NGS and phylogenetic analyses confirmed that Pt5 and Pt3 (between T0 and T1) experienced reinfection events and that Pt3 (between T1 and T2) subsequently experienced a late relapse. Pt6 relapsed; however, HCV RNA, although low, never became undetectable during treatment. Additionally, variants detected in Pt3_T1 and in Pt6_T0 were also detected in Pt3_T2 and Pt6_T1 sequences, respectively.

The recurrent HCV RNA observed post-SVR was harder to define in sample sequences from Pt1, Pt2, and Pt4. The frequency analysis of substitutions in each patient sample did not distinguish between late relapse and reinfection. Pt2 exhibited a 100% enrichment at T1 of an aa detected at 50% in T0 (R300 and A327). Pt1 and Pt4 each had only one emergent substitution detected, which was related to a nucleotide change (100% change by NGS) in the second position of the codon. These substitutions may be linked to the selection of variants associated with DAA RAS (Pt1) or due to infection with a new viral strain contracted post-SVR (Pt4).

In an effort to find a genetic distance threshold able to discriminate between late relapse and reinfection events, genetic distances between and within each sample were calculated for each patient. Since the mean genetic distances between T0 and T1 (or T1 and T2 for Pt3) were statistically significant (*p*-value <0.001) in patients assessed for potential late relapse and reinfection, differentiation by this approach was inadequate. Utilization of the Z-score, however, appeared to provide a method to discriminate between a reinfection (Pt5 and Pt3 (between the T0/T1 time-points)) and viral rebound (Pt2, Pt4, Pt6, and Pt3 (between the T1/T2 time-points)). In particular, the Z-score for the viral rebound group, except for Pt4, was <2.58, suggesting that this cut-off may be used to discriminate between reinfection and late relapse. Interestingly, Pt4 had a Z-score of 3.68, suggestive of a possible intra-familial reinfection.

How can HCV RNA recurrence be explained so long after a patient achieved an SVR? A conceivable explanation could be the presence of HCV RNA in extrahepatic sites after EOT. Although HCV is essentially hepatotropic, several studies have suggested that the virus can also infect other cells, such as peripheral blood mononuclear cells (PBMCs) and gastrointestinal mucosa (GIM) cells. In particular, stromal and neuroendocrine cells have exhibited HCV antigen positivity [35]. The existence of HCV infection and replication in PBMCs in the absence of detectable serum HCV RNA has been described in anti-HCV positive patients several years after spontaneous or antiviral-induced clearance of serum HCV RNA and normalization of transaminases [36]. Among patients treated with interferon (IFN) who subsequently relapsed, HCV RNA was often detectable in PBMCs. Moreover, DAA-treated patients who achieved SVR had an occult HCV infection with the detection of negative-strand viral genome, indicating viral replication in PBMCs despite responding to antiviral treatment [37]. GIM may also be considered as an extra-hepatic reservoir of HCV and could contribute to viral recurrence. HCV-positive gastrointestinal epithelial cells that have poor drug response could produce and release HCV particles into the blood, subsequently infecting the liver via enterohepatic circulation. Additionally, GIM cells have demonstrated positivity for the HCV-negative strand. Furthermore, quasispecies analysis indicated compartmentalization between GIM and plasma as well as GIM and liver. This is suggestive of an independent and distinct evolution of a viral population in different cellular compartments and tissues [35]. Thus, HCV particles present in the GIM may be involved in viral persistence and relapse in patients who previously achieved SVR. However, greater evidence is required to support the hypothesis that extrahepatic reservoirs, such as GIM and PBMCs, play a role in late viral rebound in HCV-infected patients who achieved SVR.

Late RNA recurrence in this case may comprise quasispecies less similar between T0 and T1, such as in Pt4. 

Our NGS data analysis, although preliminary and of limited sample size, indicates that employment of viral genetic distance to discriminate between a reinfection and late relapse [18] may be misleading. Utilization of a calculated Z-score, if confirmed in a wider patient sample size, may be a valid parameter to distinguish these two classes of viral rebound in patients. Moreover, we think that analyses from these preliminary results suggest that HCV patients who achieve SVR after DAA treatment should be monitored for longer than six months.

## 5. Conclusions

NGS analysis plays an essential role in confirming a late relapse in treated HCV-positive patients who achieved SVR. However, analysis of a larger number of cases is required to determine with accuracy an appropriate parameter suitable for distinguishing a late relapse from reinfection. Furthermore, measuring HCV RNA in PBMCs from DAA-treated patients who were HCV RNA negative at EOT may provide insight into whether the persistence of HCV RNA in extrahepatic reservoirs elicits a late relapse. Interestingly, patients retreated with certain three-DAA regimens did not experience viral recurrence (data not shown), suggesting that these regimens may have greater efficacy on extrahepatic sites as well.

## Figures and Tables

**Table 1:** NGS analysis of the number of sequence reads and clusters per patient sample.

Patient	Sampling Time	HCV GT	N. ^1^ of Raw Reads	N. of High Quality Reads (468 nt)	N. of Clustered Reads	N. of Clusters
**Pt 1**	T0	1a	34,428	9124	6942	94
	T1	1a	567,540	51,602	43,827	251
**Pt 2**	T0	1a	44,449	8147	1618	100
	T1	1a	82,626	5247	4334	59
**Pt 3**	T0	1a	164,630	6112	2734	107
	T1	1a	92,081	6295	1866	81
	T2	1a	380,424	20,958	16,882	336
**Pt 4**	T0	1b	318,426	7004	714	41
	T1	1b	199,524	5430	2426	18
**Pt 5**	T0	1a	334,655	22,187	17,184	85
	T1	1b	153,662	12,285	10,464	156
**Pt 6**	T0	3a	228,645	5657	2456	79
	T1	3a	260,292	3378	1041	19

^1^ N.: number.

**Table 2:** NGS intra-sample diversity and intra-patient nucleotide distances.

				Mean Nucleotide Distance		*p*-Value	
	Sampling	GT ^1^	Diversity (π)	T0 vs. T1	T1 vs. T2	T0 vs. T1	T1 vs. T2
**Pt 1**	T0	1a	3.84 × 10^−3^	1.13 × 10^−2^		*p* < 0.001	
	T1	1a	2.76 × 10^−3^				
**Pt 2**	T0	1a	1.64 × 10^−2^	2.02 × 10^−2^		*p* < 0.001	
	T1	1a	2.39 × 10^−3^				
**Pt 3**	T0	1a	5.98 × 10^−3^	7.05 × 10^−2^		*p* < 0.001	
	T1	1a	8.03 × 10^−3^		9.36 × 10^−3^		*p* < 0.001
	T2	1a	3.79 × 10^−3^				
**Pt 4**	T0	1b	5.32 × 10^−3^	1.37 × 10^−2^		*p* < 0.001	
	T1	1b	2.28 × 10^−3^				
**Pt 5**	T0	1a	3.40 × 10^−3^	1.98 × 10^−1^		*p* < 0.001	
	T1	1b	2.65 × 10^−3^				
**Pt 6**	T0	3a	3.88 × 10^−3^	7.14 × 10^−3^		*p* < 0.001	
	T1	3a	4.06 × 10^−3^				

^1^ GT: genotype.

**Table 3:** Z-scores absolute values; ZGT1a, ZGT1b, and ZGT3a are calculated for the same genotype of each sample, using distances between the quasispecies representative sequences and the corresponding reference sequences of the GT1a (EF407457.1), GT1b (EU781827.1), and GT3a (X76918.1).

	Pt1 (GT1a)	Pt2 (GT1a)	Pt3 (T0–T1) (GT1a)	Pt3 (T1–T2) (GT1a)	Pt4 (GT1b)	Pt5 (GT1a/1b)	Pt6 (GT3a)
Z_GT1a_	1.03	2.06	5.42	0.26	--	90.37	--
Z_GT1b_	--	--	--	--	3.68	71.09	--
Z_GT3a_	--	--	--	--	--	--	0.13

## Data Availability

The data presented in this study are available on request from the corresponding author.

## Supplementary Materials

The following are available online at https://www.mdpi.com/article/10.3390/v13061151/s1, Table S1: Frequency of substitutions in Pt1with respect to a GT1a reference sequence (EF407457.1), Table S2: Frequency of substitutions in Pt2 with respect to a GT1a reference sequence (EF407457.1), Table S3: Frequency of substitutions in Pt3 with respect to a GT1a reference sequence (EF407457.1), Table S4: Frequency of substitutions in Pt4 with respect to GT1b reference sequence (EU781827.1), Table S5: Frequency of substitutions in Pt5 with respect to GT1a (EF407457.1) or GT1b reference sequence (EU781827.1), Table S6: Frequency of substitutions in Pt6 with respect to a GT3a reference sequence (X76918.1), Figure S1: Maximum-likelihood phylogenetic tree of patient 1 sequences, Figure S2: Maximum-likelihood phylogenetic tree of patient 2 sequences, Figure S3: Maximum-likelihood phylogenetic tree of patient 3 sequences, Figure S4: Maximum-likelihood phylogenetic tree of patient 4 sequences, Figure S5: Maximum-likelihood phylogenetic tree of patient 5 sequences, Figure S6: Maximum-likelihood phylogenetic tree of patient 6 sequences, Figure S7: Maximum-likelihood phylogenetic tree of patient-derived Sanger sequences.
