# Supplementary material for: Late Relapse and Reinfection in HCV Patients Treated with Direct-Acting Antiviral (DAA) Drugs
Source: Viruses. 2021 Jun 16;13(6):1151. doi: 10.3390/v13061151 (PMC8235384; doi:10.3390/v13061151)
Supplement: Supplementary file 1 [file viruses-13-01151-s001.zip › Minosse et al_Legends suppl figures.pdf]

Figure S1. Maximum likelihood phylogenetic tree of patient 1 sequences. A substitution rate model was selected according to the Bayesian information criterion in order to build the NS5B phylogenetic tree. The HCV GT1a sequence (EF407457.1) was selected as an out-group. Sanger sequences for patient 1 samples were included and indicated as Patient_1-T0 and Patient_1-T1. The bar represents the genetic distance (substitution per nucleotide position). A bootstrap analysis with 5000 replicates was performed. Nodes with a bootstrap value of at least 80% are marked with an orange dot. Each sample from patient 1 is represented by a different color: Pt1_T0 and Pt1_T1 are depicted in light and dark blue, respectively.

Figure S2. Maximum likelihood phylogenetic tree of patient 2 sequences. A substitution rate model was selected according to the Bayesian information criterion in order to build the NS5B phylogenetic tree. The HCV GT1a sequence (EF407457.1) was selected as an out-group. Sanger sequences for patient 2 samples were included and indicated as Patient_2-T0 and Patient_2-T1. The bar represents the genetic distance (substitution per nucleotide position). A bootstrap analysis with 5000 replicates was performed. Nodes with a bootstrap value of at least 80% are marked with a violet dot. Each sample from patient 2 is represented by a different color: Pt2_T0 and Pt2_T1 are depicted in light and dark red, respectively.

Figure S3 Maximum likelihood phylogenetic tree of patient 3 sequences. A substitution rate model was selected according to the Bayesian information criterion in order to build the NS5B phylogenetic tree. The HCV GT1a sequence (EF407457.1) was selected as an out-group. Sanger sequences for patient 3 samples were included and indicated as Patient_3-T0, Patient_3-T1 and Patient_3-T2. The bar represents the genetic distance (substitution per nucleotide position). A bootstrap analysis with 5000 replicates was performed. Nodes with a bootstrap value of at least 80% are marked with an orange dot. Each sample from patient 3 is represented by a different color: Pt3_T0, Pt3_T1 and Pt3_T2 are depicted in light green, olive green and dark olive green, respectively.

Figure S4. Maximum likelihood phylogenetic tree of patient 4 sequences. A substitution rate model was selected according to the Bayesian information criterion in order to build the NS5B phylogenetic tree. The HCV GT1b sequence (EU781827.1) was selected as an out-group. Sanger sequences for patient 4 samples were included and indicated as Patient_4-T0 and Patient_4-T1. The bar represents the genetic distance (substitution per nucleotide position). A bootstrap analysis with 5000 replicates was performed. Nodes with a bootstrap value of at least 80% are marked with an orange dot. Each sample from patient 4 is represented by a different color: Pt4_T0 and Pt4_T1 are depicted in light and dark grey, respectively.

Figure S5. Maximum likelihood phylogenetic tree of patient 5 sequences. A substitution rate model was selected according to the Bayesian information criterion in order to build the NS5B phylogenetic tree. The HCV GT4d sequence (DQ418786.1) was selected as an out-group. Reference sequences (GT1a: EF407457.1; GT1b: EU781827.1) were retrieved from GenBank. Sanger sequences for patient 5 samples were included and indicated as Patient_5-T0 and Patient_5-T1. The bar represents the genetic distance (substitution per nucleotide position). A bootstrap analysis with 5000 replicates was performed. Nodes with a bootstrap value of at least 80% are marked with an orange dot. Each sample from patient 5 is represented by a different color: Pt5_T0 and Pt5_T1 are depicted in light and dark violet, respectively.

Figure S6. Maximum likelihood phylogenetic tree of patient 6 sequences. A substitution rate model was selected according to the Bayesian information criterion in order to build the NS5B phylogenetic tree. The HCV GT3a sequence (X76918.1) was selected as an out-group. Sanger sequences for patient 6 samples were included and indicated as Patient_6-T0 and Patient_6-T1. The bar represents the genetic distance (substitution per nucleotide position). A bootstrap analysis with 5000 replicates was performed. Nodes with a bootstrap value of at least 80% are marked with an orange dot. Each sample from patient

6 is represented by a different color: Pt6_T0 and Pt6_T1 are depicted in light and dark brown, respectively.

Figure S7. Maximum likelihood phylogenetic tree of our six HCV-infected patient-derived Sanger sequences. The phylogenetic tree, constructed using the maximum likelihood method (Kimura 2-parameter model+G), is based on HCV NS5B Sanger sequences. Bootstrap values were based on 500 replicates. Bootstrap values >70% are indicated on respective branches. The HCV GT4d reference sequence (DQ418786.1) was selected as an out-group. HCV reference sequences (GT1a: EF407457.1; GT1b: EU781827.1; GT3a: X76918.1) were retrieved from GenBank. Sanger sequences of each sample from each patient were indicated as Patient_N-T0, Patient_N-T1 and Patient_N-T2. The bar represents the genetic distance (substitution per nucleotide position). Each patient is represented by a different color: Pt1_T0 and Pt1_T1 are light and dark blue, respectively; Pt2_T0 and Pt2_T1 are light and dark red; Pt3_T0, Pt3_T1 and Pt3_T2 are light green, olive green and dark olive green, respectively; Pt4_T0 and Pt4_T1 are light and dark grey, respectively; Pt5_T0 and Pt5_T1 are light and dark violet, respectively; Pt6_T0 and Pt6_T1 are light and dark brown, respectively.
