# Supplementary material for: Late Relapse and Reinfection in HCV Patients Treated with Direct-Acting Antiviral (DAA) Drugs
Source: Viruses. 2021 Jun 16;13(6):1151. doi: 10.3390/v13061151 (PMC8235384; doi:10.3390/v13061151)
Supplement: Supplementary file 1 [file viruses-13-01151-s001.zip › Minosse et al_Table S1.pdf]

Table S1. Frequency of substitutions in Pt1 with respect to a GT1a reference sequence (EF407457.1). The relevant amino acid and nucleotide changes between T0 and T1 are written in red.

Pt1			Pt1										
NS5B aa position	T0 (1a)	T1 (1a)	NS5B nt position	T0 (1a) A	T0 (1a) C	T0 (1a) G	T0 (1a) T	T0 (1a) -	T1 (1a) A	T1 (1a) C	T1 (1a) G	T1 (1a) T	T1 (1a) -
179	A(0,09) V(99,91)	A(0,12) I(0,04) V(99,84)	535			100,00			0,04		99,96		
			536		0,09		99,91			0,12		99,88	
			537		100,00					100,00			
180	S(100,00)	N(0,26) S(99,74)	538	100,00					100,00				
			539			100,00			0,26		99,74		
			540		99,88		0,12			100,00			
181	R(0,10) E(0,09) K(99,81)	R(0,19) I(0,02) K(99,79)	541	99,91		0,09			100,00				
			542	99,90		0,10			99,79		0,19	0,02	
			543	100,00					100,00				
182	L(100,00)	L(100,00)	544		100,00					100,00			
			545				100,00					100,00	
			546		100,00					100,00			
183	P(100,00)	P(100,00)	547		100,00					100,00			
			548		100,00					100,00			
			549		100,00					100,00			
184	Q(100,00)	Q(100,00)	550		100,00					100,00			
			551	100,00					100,00				
			552			100,00					100,00		
185	A(93,34) V(6,66)	A(99,87) T(0,09) V(0,04)	553			100,00			0,09		99,91		
			554		93,34		6,66			99,96		0,04	
			555		100,00					99,90		0,10	
186	V(100,00)	M(0,08) V(99,92)	556			100,00			0,08		99,92		
			557				100,00					100,00	
			558			100,00					100,00		
187	M(100,00)	M(99,92) V0,08)	559	100,00					99,92		0,08		
			560				100,00					100,00	
			561			100,00					100,00		
188	G(100,00)	G(100,00)	562			100,00					100,00		
			563			100,00					100,00		
			564	100,00					100,00				
189	D(99,74) G(0,26)	D(99,83) G(0,17)	565			100,00					100,00		
			566	99,74		0,26			99,83		0,17		
			567		100,00					100,00			
190	S(100,00)	S(100,00)	568				100,00					100,00	
			569		100,00					100,00			
			570		100,00					100,00			
191	C(0,09) Y(99,91)	C(0,16) Y(99,84)	571				100,00					100,00	
			572	99,91		0,09			99,84		0,16		
			573		99,93		0,07			99,96		0,04	
192	G(100,00)	R(0,02) G(99,98)	574			100,00			0,02		99,98		
			575			100,00					100,00		
			576	100,00					100,00				
193	L(0,29) F(99,71)	F(100,00)	577		0,29		99,71					100,00	
			578				100,00					100,00	
			579		100,00					100,00			
194	Q(100,00)	Q(100,00)	580		100,00					100,00			
			581	100,00					100,00				
			582			100,00					100,00		
195	Y(100,00)	C(0,02) H(0,10) Y(99,88)	583				100,00			0,10		99,90	
			584	100,00					99,98		0,02		
			585		100,00					100,00			
196	P(0,20) S(99,80)		586		0,20		99,80		0,02	0,47		99,51	
			587		100,00					100,00			

		P(0,47) S(99,51) T(0,02)	**588**	100,00				99,86		0,14	
**197**	P(100,00)	P(100,00)	**589**		100,00				100,00		
			**590**		100,00				100,00		
			**591**	100,00				100,00			
**198**	G(100,00)	G(100,00)	**592**			100,00				100,00	
			**593**			100,00				100,00	
			**594**	100,00				100,00			
**199**	R(0,48) Q(99,52)	Q(100,00)	**595**		100,00				100,00		
			**596**	99,52		0,48		100,00			
			**597**			100,00		0,04		99,96	
**200**	R(99,47) W(0,53)	R(100,00)	**598**		99,47		0,53		100,00		
			**599**			100,00				100,00	
			**600**			100,00		0,04		99,96	
**201**	A(0,09) V(99,91)	V(100,00)	**601**			100,00				100,00	
			**602**		0,09		99,91				100,00
			**603**				100,00		0,12		99,88
**202**	E(100,00)	E(100,00)	**604**			100,00				100,00	
			**605**	100,00				100,00			
			**606**	100,00				100,00			
**203**	L(0,23) F(99,77)	L(0,49) F(99,51)	**607**		0,23		99,77		0,49		99,51
			**608**				100,00				100,00
			**609**		100,00				100,00		
**204**	L(100,00)	L(100,00)	**610**		100,00				100,00		
			**611**				100,00				100,00
			**612**		100,00			0,02	99,90		0,09
**205**	V(100,00)	A(0,20) M(0,09) V(99,71)	**613**			100,00		0,09		99,91	
			**614**				100,00		0,20		99,80
			**615**	5,27		94,73				100,00	
**206**	Q(100,00)	R(99,90) *(0,10)	**616**		100,00				99,90		0,10
			**617**	100,00						100,00	
			**618**	100,00				100,00			
**207**	A(100,00)	A(99,92) V(0,08)	**619**			100,00				100,00	
			**620**		100,00				99,92		0,08
			**621**			100,00				100,00	
**208**	W(100,00)	R(0,21) W(99,79)	**622**				100,00		0,21		99,79
			**623**			100,00				100,00	
			**624**			100,00				100,00	
**209**	K(100,00)	K(100,00)	**625**	100,00				100,00			
			**626**	100,00				100,00			
			**627**			100,00				100,00	
**210**	S(100,00)	S(100,00)	**628**				100,00				100,00
			**629**		100,00				100,00		
			**630**		100,00				100,00		
**211**	E(0,09) K(99,91)	K(100,00)	**631**	99,91		0,09		100,00			
			**632**	100,00				100,00			
			**633**			100,00				100,00	
**212**	K(100,00)	K(100,00)	**634**	100,00				100,00			
			**635**	100,00				100,00			
			**636**			100,00				100,00	
**213**	T(100,00)	T(100,00)	**637**	100,00				100,00			
			**638**		100,00				100,00		
			**639**		100,00				100,00		
**214**	P(100,00)	P(100,00)	**640**		100,00				100,00		
			**641**		100,00				100,00		
			**642**			100,00				100,00	
**215**	M(100,00)	M(99,87) T(0,10) V(0,03)	**643**	100,00				99,97		0,03	
			**644**				100,00		0,10		99,90
			**645**			100,00				100,00	
**216**	G(100,00)	G(100,00)	**646**			100,00				100,00	
			**647**			100,00				100,00	

			**648**			100,00				100,00	
**217**	L(0,20) F(99,80)	L(0,50) F(99,50)	**649**		0,20		99,80		0,50		99,50
			**650**				100,00				100,00
			**651**		100,00				100,00		
**218**	S(100,00)	P(0,02) S(99,98)	**652**				100,00		0,02		99,98
			**653**		100,00				100,00		
			**654**	0,07		99,93		0,06		99,94	
**219**	Y(100,00)	C(0,06) H(0,10) Y(99,84)	**655**				100,00		0,10		99,90
			**656**	100,00				99,94		0,06	
			**657**				100,00		0,11		99,89
**220**	D(99,93 G(0,07)	N(0,03) D(99,97)	**658**			100,00		0,03		99,97	
			**659**	99,93		0,07		100,00			
			**660**				100,00				100,00
**221**	A(0,13) T(99,87)	A(0,03) T(99,97)	**661**	99,87		0,13		99,97		0,03	
			**662**		100,00				100,00		
			**663**		100,00				100,00		
**222**	R(100,00)	R(99,88) H(0,12)	**664**		100,00				100,00		
			**665**			100,00		0,12		99,88	
			**666**		100,00				100,00		
**223**	R(0,07) C(99,93)	C(99,99) S(0,01)	**667**		0,07		99,93	0,01			99,99
			**668**			100,00				100,00	
			**669**		100,00				100,00		
**224**	F(100,00)	F(100,00)	**670**				100,00				100,00
			**671**				100,00				100,00
			**672**				100,00				100,00
**225**	D(99,84) G(0,16)	D(99,82) G(0,16) V(0,02)	**673**			100,00				100,00	
			**674**	99,84		0,16		99,82		0,16	0,02
			**675**		100,00				100,00		
**226**	S(100,00)	S(100,00)	**676**				100,00				100,00
			**677**		100,00				100,00		
			**678**		100,00				100,00		
**227**	A(0,07) T(99,93)	A(0,16) T(99,84)	**679**	99,93		0,07		99,84		0,16	
			**680**		100,00				100,00		
			**681**	83,95		16,05		99,97			0,03
**228**	A(0,12) V(99,88)	A(0,62) V(99,38)	**682**			100,00				100,00	
			**683**		0,12		99,88		0,62		99,38
			**684**		100,00				100,00		
**229**	T(100,00)	A(0,15) T(99,85)	**685**	100,00				99,85		0,15	
			**686**		100,00				100,00		
			**687**		0,09		99,91		0,04		99,96
**230**	E(100,00)	E(99,50) V(0,04) X(0,46)	**688**			100,00				99,53	
			**689**	100,00				99,50			0,04
			**690**			100,00				100,00	
**231**	N(0,17) S(99,54) X(0,29)	S(100,00)	**691**	99,71				100,00			
			**692**	0,17		99,54				100,00	
			**693**		76,22		23,78		99,98		0,02
**232**	D(100,00)	D(100,00)	**694**			100,00				100,00	
			**695**	100,00				100,00			
			**696**		100,00				100,00		
**233**	I(100,00)	I(99,88) V(0,12)	**697**	100,00				99,88		0,12	
			**698**				100,00				100,00
			**699**		100,00				100,00		
**234**	R(99,93) H(0,07)	R(99,97) H(0,03)	**700**		100,00				100,00		
			**701**	0,07		99,93		0,03		99,97	
			**702**				100,00		0,07		99,93
**235**	T(100,00)	A(0,15) T(99,85)	**703**	100,00				99,85		0,15	
			**704**		100,00				100,00		
			**705**			100,00				100,00	
**236**	E(100,00)	E(100,00)	**706**			100,00				100,00	
			**707**	100,00				100,00			
			**708**			100,00				100,00	
**237**	E(100,00)	E(100,00)	**709**			100,00				100,00	

			**710**	100,00				100,00			
			**711**	100,00				100,00			
**238**	A(100,00)	A(99,95) T(0,05)	**712**			100,00		0,05		99,95	
			**713**		100,00				100,00		
			**714**	99,91		0,09		100,00			
**239**	I(99,91) T(0,09)	I(99,97) V(0,03)	**715**	100,00				99,97		0,03	
			**716**		0,09		99,91				100,00
			**717**		100,00				100,00		
**240**	Y(100,00)	H(0,15) Y(99,85)	**718**				100,00		0,15		99,85
			**719**	100,00				100,00			
			**720**		100,00				99,95		0,05
**241**	Q(100,00)	R(0,14) Q(99,81) L(0,02) *(0,03)	**721**		100,00				99,97		0,03
			**722**	100,00				99,84		0,13	0,02
			**723**			100,00				100,00	
**242**	C(100,00)	R(0,06) C(99,90) Y(0,04)	**724**				100,00		0,05		99,95
			**725**			100,00		0,04		99,96	
			**726**		0,12		99,88		0,20		99,80
**243**	R(0,12) C(99,88)	C(99,93) Y(0,05) *(0,02)	**727**		0,12		99,88				100,00
			**728**			100,00		0,05		99,95	
			**729**		0,07		99,93	0,02	0,19		99,79
**244**	D(99,80) G(0,20)	D(100,00)	**730**			100,00				100,00	
			**731**	99,80		0,20		100,00			
			**732**		99,90		0,10		99,97		0,03
**245**	L(100,00)	L(100,00)	**733**		100,00				100,00		
			**734**				100,00				100,00
			**735**			100,00				100,00	
**246**	D(100,00)	D(100,00)	**736**			100,00				100,00	
			**737**	100,00				100,00			
			**738**		100,00				100,00		
**247**	P(100,00)	P(100,00)	**739**		100,00				100,00		
			**740**		100,00				100,00		
			**741**		100,00				100,00		
**248**	Q(100,00)	R(0,12) Q(99,88)	**742**		100,00				100,00		
			**743**	100,00				99,88		0,12	
			**744**	100,00				100,00			
**249**	A(100,00)	A(100,00)	**745**			100,00				100,00	
			**746**		100,00				100,00		
			**747**		100,00				99,94		0,06
**250**	R(100,00)	R(99,98) H(0,02)	**748**		100,00				100,00		
			**749**			100,00		0,02		99,98	
			**750**		0,07		99,93		0,12		99,88
**251**	V(100,00)	A(0,17) M(0,04) V(99,79)	**751**			100,00		0,04		99,96	
			**752**				100,00		0,17		99,83
			**753**			100,00				100,00	
**252**	A(100,00)	A(100,00)	**754**			100,00				100,00	
			**755**		100,00				100,00		
			**756**		100,00				100,00		
**253**	I(100,00)	I(99,71) T(0,13) V(0,16)	**757**	100,00				99,84		0,16	
			**758**				100,00		0,13		99,87
			**759**		100,00				100,00		
**254**	K(100,00)	R(0,03) K(99,97)	**760**	100,00				100,00			
			**761**	100,00				99,97		0,03	
			**762**			100,00				100,00	
**255**	S(100,00)	S(100,00)	**763**				100,00				100,00
			**764**		100,00				100,00		
			**765**		100,00				100,00		
**256**	L(100,00)	L(100,00)	**766**		100,00				100,00		
			**767**				100,00				100,00
			**768**		100,00						100,00
**257**	T(100,00)	A(0,07) T(99,93)	**769**	100,00				99,93		0,07	
			**770**		100,00				100,00		

			**771**				100,00				100,00
**258**	E(100,00)	E(100,00)	**772**			100,00				100,00	
			**773**	100,00				100,00			
			**774**			100,00				100,00	
**259**	R(100,00)	R(99,94) G(0,06)	**775**	100,00				99,94		0,06	
			**776**			100,00				100,00	
			**777**			100,00				100,00	
**260**	L(100,00)	L(100,00)	**778**		100,00				100,00		
			**779**				100,00				100,00
			**780**				100,00				100,00
**261**	Y(100,00)	Y(100,00)	**781**				100,00				100,00
			**782**	100,00				100,00			
			**783**				100,00		0,16		99,84
**262**	A(0,09) V(99,91)	V(100,00)	**784**			100,00				100,00	
			**785**		0,09		99,91				100,00
			**786**				100,00				100,00
**263**	G(100,00)	G(100,00)	**787**			100,00				100,00	
			**788**			100,00				100,00	
			**789**			100,00				100,00	
**264**	G(100,00)	G(100,00)	**790**			100,00				100,00	
			**791**			100,00				100,00	
			**792**		100,00				100,00		
**265**	P(100,00)	P(100,00)	**793**		100,00				100,00		
			**794**		100,00				100,00		
			**795**				100,00				100,00
**266**	L(100,00)	R(0,01) L(99,99)	**796**		100,00				100,00		
			**797**				100,00			0,01	99,99
			**798**				100,00		0,06		99,94
**267**	T(100,00)	T(100,00)	**799**	100,00				100,00			
			**800**		100,00				100,00		
			**801**		100,00				100,00		
**268**	N(100,00)	N(100,00)	**802**	100,00				100,00			
			**803**	100,00				100,00			
			**804**		100,00						100,00
**269**	P(0,09) S(99,91)	S(100,00)	**805**		0,09		99,91				100,00
			**806**		100,00				100,00		
			**807**	100,00				99,92		0,08	
**270**	R(100,00)	R(100,00)	**808**	100,00				100,00			
			**809**			100,00				100,00	
			**810**			100,00				100,00	
**271**	G(100,00)	G(100,00)	**811**			100,00				100,00	
			**812**			100,00				100,00	
			**813**			100,00				100,00	
**272**	E(100,00)	E(100,00)	**814**			100,00				100,00	
			**815**	100,00				100,00			
			**816**			100,00				100,00	
**273**	N(100,00)	N(100,00)	**817**	100,00				100,00			
			**818**	100,00				100,00			
			**819**		100,00				100,00		
**274**	C(100,00)	C(99,93) Y(0,07)	**820**				100,00				100,00
			**821**			100,00		0,07		99,93	
			**822**		100,00				99,99		0,01
**275**	G(100,00)	D(0,05) G(99,95)	**823**			100,00				100,00	
			**824**			100,00		0,05		99,95	
			**825**		100,00				100,00		
**276**	Y(100,00)	C(0,10) H(0,03) Y(99,87)	**826**				100,00		0,03		99,97
			**827**	100,00				99,90		0,10	
			**828**		4,88		95,12				100,00
**277**	R(100,00)	R(99,86) C(0,06) H(0,08)	**829**		100,00				99,94		0,06
			**830**			100,00		0,08		99,92	
			**831**		97,18		2,82		99,97		0,03
**278**	R(100,00)	R(100,00)	**832**	100,00				100,00			

			**833**			100,00				100,00	
			**834**			100,00				100,00	
**279**	C(100,00)	C(100,00)	**835**				100,00				100,00
			**836**			100,00				100,00	
			**837**		100,00				100,00		
**280**	R(100,00)	R(99,91) H(0,09)	**838**		100,00				100,00		
			**839**			100,00		0,09		99,91	
			**840**		100,00				99,92		0,08
**281**	A(100)	A(99,89) T(0,03) V(0,08)	**841**			100,00		0,03		99,97	
			**842**		100,00				99,92		0,08
			**843**	0,07		99,93				100,00	
**282**	S(100,00)	G(0,07) S(99,93)	**844**	100,00				99,93		0,07	
			**845**			100,00				100,00	
			**846**		100,00				99,94		0,06
**283**	G(100,00)	G(100,00)	**847**			100,00				100,00	
			**848**			100,00				100,00	
			**849**		100,00				100,00		
**284**	V(100,00)	A(0,10) I(0,07) V(99,83)	**850**			100,00		0,07		99,93	
			**851**				100,00		0,10		99,90
			**852**	100,00				100,00			
**285**	L(100,00)	L(100,00)	**853**		100,00				100,00		
			**854**				100,00				100,00
			**855**			100,00				100,00	
**286**	A(0,13) T(99,87)	T(100,00)	**856**	99,87		0,13		100,00			
			**857**		100,00				100,00		
			**858**	100,00				99,83		0,17	
**287**	T(100,00)	T(100,00)	**859**	100,00				100,00			
			**860**		100,00				100,00		
			**861**		100,00				100,00		
**288**	S(100,00)	C(0,02) G(0,08) S(99,90)	**862**	100,00				99,90		0,08	0,02
			**863**			100,00				100,00	
			**864**		100,00				99,97		0,03
**289**	C(100,00)	R(0,25) C(99,68) S(0,03) Y(0,04)	**865**				100,00	0,03	0,25		99,71
			**866**			100,00		0,04		99,96	
			**867**				100,00		0,13		99,87
**290**	G(100,00)	D(0,14) G(99,86)	**868**			100,00				100,00	
			**869**			100,00		0,14		99,86	
			**870**				100,00		0,01		99,99
**291**	N(100,00)	N(99,83) D(0,17)	**871**	100,00				99,83		0,17	
			**872**	100,00				100,00			
			**873**		100,00				99,93		0,07
**292**	A(0,07) T(99,93)	A(0,25) T(99,75)	**874**	99,93		0,07		99,75		0,25	
			**875**		100,00				100,00		
			**876**		100,00				100,00		
**293**	L(100,00)	L(100,00)	**877**		100,00				100,00		
			**878**				100,00				100,00
			**879**		83,17		16,83		99,96		0,04
**294**	T(100,00)	A(0,11) T(99,89)	**880**	100,00				99,89		0,11	
			**881**		100,00				100,00		
			**882**				100,00				100,00
**295**	C(100,00)	C(99,95) Y(0,05)	**883**				100,00				100,00
			**884**			100,00		0,05		99,95	
			**885**		100,00				100,00		
**296**	Y(100,00)	C(0,18) H(0,09) Y(99,74)	**886**				100,00		0,09		99,91
			**887**	100,00				99,82		0,18	
			**888**		100,00				100,00		
**297**	I(99,76) V(0,13)	I(99,95) V(0,05)	**889**	99,87		0,13		99,95		0,05	
			**890**		0,12		99,88				100,00
			**891**		100,00				100,00		
**298**	E(0,09) K(99,91)	K(100,00)	**892**	99,91		0,09		100,00			
			**893**	100,00				100,00			

			**894**			100,00				100,00	
**299**	A(100,00)	A(100,00)	**895**			100,00				100,00	
			**896**		100,00				100,00		
			**897**		100,00				99,97		0,03
**300**	R(100,00)	R(100,00)	**898**		100,00				100,00		
			**899**			100,00				100,00	
			**900**	100,00				75,12		24,85	0,03
**301**	A(100,00)	A(99,96) V(0,04)	**901**			100,00				100,00	
			**902**		100,00				99,96		0,04
			**903**	100,00				99,88		0,12	
**302**	A(100,00)	A(100,00)	**904**			100,00				100,00	
			**905**		100,00				100,00		
			**906**		100,00				100,00		
**303**	C(100,00)	C(99,99) S(0,01)	**907**				100,00	0,01			99,99
			**908**			100,00				100,00	
			**909**				100,00		0,39		99,61
**304**	R(100,00)	R(99,77) Q(0,14) *(0,09)	**910**		100,00				99,91		0,09
			**911**			100,00		0,14		99,86	
			**912**	100,00				100,00			
**305**	A(99,91) V(0,09)	A(99,99) V(0,01)	**913**			100,00				100,00	
			**914**		99,91		0,09		99,99		0,01
			**915**		100,00				99,93		0,07
**306**	A(99,83) T(0,17)	A(99,94) T(0,05) V(0,01)	**916**	0,17		99,83		0,05		99,95	
			**917**		100,00				99,99		0,01
			**918**	100,00				100,00			
**307**	G(100,00)	G(100,00)	**919**			100,00				100,00	
			**920**			100,00				100,00	
			**921**			100,00				100,00	
**308**	L(100,00)	L(100,00)	**922**		100,00				100,00		
			**923**				100,00				100,00
			**924**		100,00				100,00		
**309**	R(100,00)	R(99,95) W(0,05)	**925**		100,00				99,95		0,05
			**926**			100,00				100,00	
			**927**			100,00				100,00	
**310**	D(99,93)G(0,07)	D(100,00)	**928**			100,00				100,00	
			**929**	99,93		0,07		100,00			
			**930**		100,00				99,94		0,06
**311**	C(100,00)	R(0,23) C(99,74) S(0,03)	**931**				100,00	0,03	0,23		99,74
			**932**			100,00				100,00	
			**933**		100,00				99,99		0,01
**312**	A(0,09) T(99,91)	A(0,20) I(0,06) T(99,74)	**934**	99,91		0,09		99,80		0,20	
			**935**		100,00				99,94		0,06
			**936**		100,00				100,00		
**313**	M(99,93) T(0,07)	M(99,74) T(0,17) V(0,09)	**937**	100,00				99,91		0,09	
			**938**		0,07		99,93		0,17		99,83
			**939**			100,00				100,00	
**314**	L(100,00)	L(99,51) F(0,07) P(0,41)	**940**		100,00				99,93		0,07
			**941**				100,00		0,41		99,59
			**942**		99,91		0,09		99,91		0,09
**315**	V(100,00)	A(0,20) V(99,80)	**943**			100,00				100,00	
			**944**				100,00		0,20		99,80
			**945**			100,00		0,02		99,98	
**316**	R(0,07) C(99,93)	R(0,05) C(99,92) *(0,03)	**946**		0,07		99,93		0,05		99,95
			**947**			100,00				100,00	
			**948**				100,00	0,03			99,97
**317**	G(100,00)	D(0,07) G(99,93)	**949**			100,00				100,00	
			**950**			100,00		0,07		99,93	
			**951**		100,00				99,90		0,10
**318**	D(100,00)	D(99,86) G(0,14)	**952**			100,00				100,00	
			**953**	100,00				99,86		0,14	
			**954**		100,00				100,00		
**319**	D(100,00)		**955**			100,00		0,14		99,86	

		N(0,14) D(99,86)	**956**	100,00				100,00			
			**957**		100,00				100,00		
**320**	L(100,00)	L(100,00)	**958**				100,00				100,00
			**959**				100,00				100,00
			**960**	100,00				99,82		0,18	
**321**	V(100,00)	I(0,03) V(99,97)	**961**			100,00		0,03		99,97	
			**962**				100,00				100,00
			**963**		100,00				99,93		0,07
**322**	A(0,13) V(99,87)	A(0,13) I(0,05) V(99,82)	**964**			100,00		0,05		99,95	
			**965**		0,13		99,87		0,13		99,87
			**966**				100,00				100,00
**323**	I(99,81) T(0,09) V(0,10)	I(99,91) V(0,09)	**967**	99,90		0,10		99,91		0,09	
			**968**		0,09		99,91				100,00
			**969**		100,00				99,96		0,04
**324**	R(0,09) C(99,91)	C(99,97) Y(0,03)	**970**		0,09		99,91				100,00
			**971**			100,00		0,03		99,97	
			**972**				100,00		0,20		99,80
**325**	E(100,00)	E(100,00)	**973**			100,00				100,00	
			**974**	100,00				100,00			
			**975**	100,00				100,00			
**326**	S(100,00)	S(100,00)	**976**	100,00				100,00			
			**977**			100,00				100,00	
			**978**				100,00		0,12		99,88
**327**	A(100,00)	A(99,92) T(0,01) V(0,07)	**979**			100,00		0,01		99,99	
			**980**		100,00				99,93		0,07
			**981**			100,00				100,00	
**328**	G(100,00)	G(100,00)	**982**			100,00				100,00	
			**983**			100,00				100,00	
			**984**			100,00		0,01		99,99	
**329**	V(100,00)	L(0,01) V(99,99)	**985**			100,00			0,01	99,99	
			**986**				100,00				100,00
			**987**		100,00				99,99	0,01	
**330**	Q(100,00)	Q(99,81) L(0,01) *(0,18)	**988**		100,00				99,82		0,18
			**989**	100,00				99,99			0,01
			**990**			100,00				100,00	
**331**	E(100,00)	E(100,00)	**991**			100,00				100,00	
			**992**	100,00				100,00			
			**993**	100,00				100,00			
**332**	D(100,00)	D(99,65) G(0,35)	**994**			100,00				100,00	
			**995**	100,00				99,65		0,35	
			**996**		100,00				97,94		2,06
**333**	A(100,00)	A(99,92) V(0,08)	**997**			100,00				100,00	
			**998**		100,00				99,92		0,08
			**999**			100,00		0,04		99,96	
**334**	A(100,00)	A(99,96) T(0,01) V(0,03)	**1000**			100,00		0,01		99,99	
			**1001**		100,00				99,97		0,03
			**1002**			100,00		0,03		99,97	
