# Supplementary material for: Late Relapse and Reinfection in HCV Patients Treated with Direct-Acting Antiviral (DAA) Drugs
Source: Viruses. 2021 Jun 16;13(6):1151. doi: 10.3390/v13061151 (PMC8235384; doi:10.3390/v13061151)
Supplement: Supplementary file 1 [file viruses-13-01151-s001.zip › Minosse et al_Table S2.pdf]

Table S2. Frequency of substitutions in Pt2 with respect to a GT1a reference sequence (EF407457.1). The relevant amino acid and nucleotide changes between T0 and T1 are written in red.

Pt2				Pt2									
NS5B aa position	T0 (1a)	T1 (1a)	NS5B nt position	T0 (1a)					T1 (1a)				
				A	C	G	T	-	A	C	G	T	-
			535			100,00					100,00		
179	V(100,00)	V(100,00)	536				100,00					100,00	
			537				100,00					100,00	
			538	100,00					100,00				
180	S(100,00)	S(100,00)	539			100,00					100,00		
			540		98,33		1,67			100,00			
			541	100,00					100,00				
181	K(100,00)	K(100,00)	542	100,00					100,00				
			543	100,00							100,00		
			544		100,00					100,00			
182	L(100,00)	L(100,00)	545				100,00					100,00	
			546		100,00					100,00			
			547		100,00					100,00			
183	P(100,00)	P(100,00)	548		100,00					100,00			
			549		100,00					100,00			
			550		100,00					100,00			
184	P(100,00)	P(100,00)	551		100,00					100,00			
			552	23,49	76,51				100,00				
			553			100,00					100,00		
185	A(100,00)	A(100,00)	554		100,00					100,00			
			555		100,00					99,79		0,21	
			556			100,00					100,00		
186	V(100,00)	V(100,00)	557				100,00					100,00	
			558			100,00					100,00		
			559	100,00					99,72		0,28		
187	M(100,00)	M(99,72) V(0,28)	560				100,00					100,00	
			561			100,00					100,00		
			562			100,00					100,00		
188	G(100,00)	G(100,00)	563			100,00					100,00		
			564	100,00					100,00				
			565	100,00					100,00				
189	S(100,00)	S(100,00)	566			100,00					100,00		
			567		100,00					100,00			
			568				100,00					100,00	
190	S(100,00)	S(100,00)	569		100,00					100,00			
			570		100,00					100,00			
			571				100,00					100,00	
191	Y(100,00)	C(0,12) Y(99,88)	572	100,00					99,88		0,12		
			573		100,00					100,00			
			574			100,00					100,00		
192	G(100,00)	G(100,00)	575			100,00					100,00		
			576	100,00					100,00				
			577				100,00			0,25		99,75	
193	F(100,00)	L(0,25) F(99,75)	578				100,00					100,00	
			579		100,00					100,00			
			580		100,00					100,00			
194	Q(100,00)	Q(100,00)	581	100,00					100,00				
			582	73,18		26,82					100,00		
			583				100,00					100,00	
195	Y(100,00)	Y(100,00)	584	100,00					100,00				
			585		100,00					100,00			
			586				100,00			0,28		99,72	
196	S(100,00)	P(0,28) S(99,72)	587		100,00					100,00			
			588	100,00					99,77		0,23		

			589		100,00				100,00		
197	P(100,00)	P(100,00)	590		100,00				100,00		
			591	100,00				100,00			
			592			100,00				100,00	
198	G(100,00)	G(100,00)	593			100,00				100,00	
			594	100,00				100,00			
			595		100,00				100,00		
199	Q(100,00)	R(0,14) Q(99,86)	596	100,00				99,86		0,14	
			597			100,00				100,00	
			598		100,00				100,00		
200	R(100,00)	R(100,00)	599			100,00				100,00	
			600			100,00				100,00	
			601			100,00				100,00	
201	V(100,00)	A(0,18) V(99,82)	602				100,00		0,18		99,82
			603				100,00				100,00
			604			100,00				100,00	
202	E(100,00)	E(100,00)	605	100,00				100,00			
			606	99,32		0,68		100,00			
			607				100,00				100,00
203	F(100,00)	F(100,00)	608				100,00				100,00
			609		100,00				100,00		
			610		100,00				100,00		
204	L(100,00)	L(100,00)	611				100,00				100,00
			612		100,00				100,00		
			613			100,00				100,00	
205	V(100,00)	A(0,16) V(99,84)	614				100,00		0,16		99,84
			615			100,00				100,00	
			616		100,00				100,00		
206	Q(100,00)	Q(100,00)	617	100,00				100,00			
			618	100,00				100,00			
			619			100,00				100,00	
207	A(100,00)	A(100,00)	620		100,00				100,00		
			621			100,00				100,00	
			622				100,00				100,00
208	W(100,00)	W(100,00)	623			100,00				100,00	
			624			100,00				100,00	
			625	100,00				100,00			
209	R(0,68) K(99,32)	K(100,00)	626	99,32		0,68		100,00			
			627			100,00				100,00	
			628				100,00				100,00
210	S(100,00)	S(100,00)	629		100,00				100,00		
			630		99,13		0,87		100,00		
			631	100,00				100,00			
211	K(100,00)	K(100,00)	632	100,00				100,00			
			633			100,00				100,00	
			634	100,00				100,00			
212	K(100,00)	K(100,00)	635	100,00				100,00			
			636			100,00				100,00	
			637	100,00				99,82		0,18	
213	T(100,00)	A(0,18) T(99,82)	638		100,00				100,00		
			639		100,00				100,00		
			640		100,00				100,00		
214	P(100,00)	P(100,00)	641		100,00				100,00		
			642	92,89		7,11		100,00			
			643	100,00				100,00			
215	M(100,00)	M(100,00)	644				100,00				100,00
			645			100,00				100,00	
			646			100,00				100,00	
216	G(100,00)	G(100,00)	647			100,00				100,00	
			648			100,00				100,00	
217	F(100,00)	F(100,00)	649				100,00				100,00
			650				100,00				100,00

			**651**		100,00				100,00		
			**652**				100,00				100,00
**218**	S(100,00)	S(100,00)	**653**		100,00				100,00		
			**654**			100,00				100,00	
			**655**				100,00		0,12		99,88
**219**	Y(100,00)	H(0,12)<br>Y(99,88)	**656**	100,00				100,00			
			**657**				100,00				100,00
			**658**			100,00				100,00	
**220**	D(100,00)	D(100,00)	**659**	100,00				100,00			
			**660**				100,00				100,00
			**661**	100,00				100,00			
**221**	T(100,00)	T(100,00)	**662**		100,00				100,00		
			**663**		100,00				100,00		
			**664**		100,00				100,00		
**222**	R(100,00)	R(100,00)	**665**			100,00				100,00	
			**666**		100,00				100,00		
			**667**				100,00				100,00
**223**	C(100,00)	C(100,00)	**668**			100,00				100,00	
			**669**				100,00				100,00
			**670**				100,00				100,00
**224**	F(100,00)	F(100,00)	**671**				100,00				100,00
			**672**				100,00				100,00
			**673**			100,00				100,00	
**225**	D(100,00)	D(100,00)	**674**	100,00				100,00			
			**675**		100,00				100,00		
			**676**				100,00	0,14			99,86
**226**	S(100,00)	S(99,86)<br>T(0,14)	**677**		100,00				100,00		
			**678**		63,66		36,34		0,23		99,77
			**679**	100,00				100,00			
**227**	T(100,00)	T(100,00)	**680**		100,00				100,00		
			**681**	100,00				99,63		0,37	
			**682**			100,00				100,00	
**228**	V(100,00)	V(100,00)	**683**				100,00				100,00
			**684**		100,00				100,00		
			**685**	100,00				99,84		0,16	
**229**	T(100,00)	A(0,16)<br>T(99,84)	**686**		100,00				100,00		
			**687**				100,00		0,12		99,88
			**688**			100,00				99,45	
**230**	E(100,00)	E(99,45)<br>X(0,55)	**689**	100,00				99,45			
			**690**			100,00				100,00	
			**691**	100,00				100,00			
**231**	S(100,00)	S(100,00)	**692**			100,00				100,00	
			**693**		97,59		2,41		99,68		0,32
			**694**			100,00				100,00	
**232**	D(100,00)	D(99,68)<br>G(0,32)	**695**	100,00				99,68		0,32	
			**696**		100,00				100,00		
			**697**	100,00				100,00			
**233**	I(100,00)	I(100,00)	**698**				100,00				100,00
			**699**		100,00				100,00		
			**700**		100,00				100,00		
**234**	R(100,00)	R(100,00)	**701**			100,00				100,00	
			**702**				100,00				100,00
			**703**	100,00				100,00			
**235**	T(100,00)	T(100,00)	**704**		100,00				100,00		
			**705**			100,00		0,14		99,86	
			**706**			100,00				100,00	
**236**	E(100,00)	E(100,00)	**707**	100,00				100,00			
			**708**			100,00				100,00	
			**709**			100,00				100,00	
**237**	E(100,00)	E(100,00)	**710**	100,00				100,00			
			**711**			100,00				100,00	
**238**	A(100,00)		**712**			100,00		0,14		99,86	

		A(99,86) T(0,14)	**713**		100,00				100,00		
			**714**	100,00				100,00			
			**715**	100,00				99,82		0,18	
**239**	I(100,00)	I(99,82) V(0,18)	**716**				100,00				100,00
			**717**				100,00				100,00
			**718**				100,00				100,00
**240**	Y(100,00)	Y(100,00)	**719**	100,00				100,00			
			**720**		100,00				100,00		
			**721**		100,00				100,00		
**241**	Q(100,00)	R(0,14) Q(99,86)	**722**	100,00				99,86		0,14	
			**723**	100,00				100,00			
			**724**				100,00				100,00
**242**	C(100,00)	C(100,00)	**725**			100,00				100,00	
			**726**				100,00				100,00
			**727**				100,00		0,28		99,72
**243**	C(100,00)	R(0,28) C(99,72)	**728**			100,00				100,00	
			**729**				100,00		0,21		99,79
			**730**			100,00				100,00	
**244**	D(100,00)	D(99,84) G(0,16)	**731**	100,00				99,84		0,16	
			**732**		100,00				100,00		
			**733**		100,00				100,00		
**245**	L(100,00)	L(100,00)	**734**				100,00				100,00
			**735**			100,00				100,00	
			**736**			100,00				100,00	
**246**	D(100,00)	D(100,00)	**737**	100,00				100,00			
			**738**		100,00				100,00		
			**739**		100,00				100,00		
**247**	P(100,00)	P(100,00)	**740**		100,00				100,00		
			**741**		100,00				100,00		
			**742**		100,00				100,00		
**248**	Q(100,00)	Q(100,00)	**743**	100,00				100,00			
			**744**	100,00				100,00			
			**745**			100,00				100,00	
**249**	A(100,00)	A(100,00)	**746**		100,00				100,00		
			**747**		96,97		3,03		100,00		
			**748**		100,00				100,00		
**250**	R(100,00)	R(100,00)	**749**			100,00				100,00	
			**750**		48,52		51,48				100,00
			**751**	0,74		99,26				100,00	
**251**	M(0,74) V(99,26)	V(100,00)	**752**				100,00				100,00
			**753**			100,00				100,00	
			**754**			100,00				100,00	
**252**	A(100,00)	A(100,00)	**755**		100,00				100,00		
			**756**		100,00				100,00		
			**757**	100,00				100,00			
**253**	I(100,00)	I(99,84) T(0,16)	**758**				100,00		0,16		99,84
			**759**		98,89		1,11		100,00		
			**760**	100,00				100,00			
**254**	K(100,00)	K(100,00)	**761**	100,00				100,00			
			**762**			100,00				100,00	
			**763**				100,00				100,00
**255**	S(100,00)	S(100,00)	**764**		100,00				100,00		
			**765**		100,00				100,00		
			**766**		100,00				100,00		
**256**	L(100,00)	L(100,00)	**767**				100,00				100,00
			**768**		100,00				100,00		
			**769**	100,00				99,86		0,14	
**257**	T(100,00)	A(0,14) T(99,86)	**770**		100,00				100,00		
			**771**				100,00				100,00
			**772**			100,00				100,00	
**258**	E(100,00)	E(100,00)	**773**	100,00				100,00			
			**774**			100,00				100,00	

			**775**	100,00				100,00			
**259**	R(100,00)	R(100,00)	**776**			100,00				100,00	
			**777**			100,00				100,00	
			**778**		100,00				100,00		
**260**	L(100,00)	L(100,00)	**779**				100,00				100,00
			**780**				100,00				100,00
			**781**				100,00				100,00
**261**	Y(100,00)	Y(100,00)	**782**	100,00				100,00			
			**783**				100,00				100,00
			**784**			100,00				100,00	
**262**	V(100,00)	V(100,00)	**785**				100,00				100,00
			**786**		41,66		58,34		100,00		
			**787**			100,00				100,00	
**263**	G(100,00)	G(100,00)	**788**			100,00				100,00	
			**789**			100,00				100,00	
			**790**			100,00				100,00	
**264**	G(100,00)	G(100,00)	**791**			100,00				100,00	
			**792**		100,00				100,00		
			**793**		100,00				100,00		
**265**	P(100,00)	P(100,00)	**794**		100,00				100,00		
			**795**				100,00				100,00
			**796**		100,00				100,00		
**266**	L(100,00)	L(100,00)	**797**				100,00				100,00
			**798**		41,66		58,34		100,00		
			**799**	100,00				100,00			
**267**	T(100,00)	T(100,00)	**800**		100,00				100,00		
			**801**		100,00				100,00		
			**802**	100,00				100,00			
**268**	N(100,00)	N(100,00)	**803**	100,00				100,00			
			**804**				100,00		0,21		99,79
			**805**				100,00				100,00
**269**	S(100,00)	S(100,00)	**806**		100,00				100,00		
			**807**	100,00				100,00			
			**808**	100,00				100,00			
**270**	R(100,00)	R(100,00)	**809**			100,00				100,00	
			**810**			100,00				100,00	
			**811**			100,00				100,00	
**271**	G(100,00)	G(100,00)	**812**			100,00				100,00	
			**813**			100,00				100,00	
			**814**			100,00				100,00	
**272**	E(100,00)	E(100,00)	**815**	100,00				100,00			
			**816**			100,00				100,00	
			**817**	100,00				99,77	0,23		
**273**	N(100,00)	N(99,77) H(0,23)	**818**	100,00				100,00			
			**819**		100,00				100,00		
			**820**				100,00				100,00
**274**	C(100,00)	C(100,00)	**821**			100,00				100,00	
			**822**		100,00				100,00		
			**823**			100,00				100,00	
**275**	G(100,00)	G(100,00)	**824**			100,00				100,00	
			**825**		100,00				100,00		
			**826**				100,00				100,00
**276**	Y(100,00)	Y(100,00)	**827**	100,00				100,00			
			**828**		100,00				99,88		0,12
			**829**		100,00				99,88		0,12
**277**	R(100,00)	R(99,88) C(0,12)	**830**			100,00				100,00	
			**831**		100,00				100,00		
			**832**	100,00				100,00			
**278**	R(100,00)	R(100,00)	**833**			100,00				100,00	
			**834**			100,00				100,00	
**279**	C(100,00)	C(100,00)	**835**				100,00				100,00
			**836**			100,00				100,00	

			**837**		100,00				100,00		
			**838**		100,00				100,00		
**280**	R(100,00)	R(99,70) H(0,30)	**839**			100,00		0,30		99,70	
			**840**		100,00				100,00		
			**841**			100,00				100,00	
**281**	A(100,00)	A(100,00)	**842**		100,00				100,00		
			**843**	0,37	47,84	51,79			100,00		
			**844**	100,00				100,00			
**282**	S(100,00)	S(100,00)	**845**			100,00				100,00	
			**846**		100,00				100,00		
			**847**			100,00				100,00	
**283**	G(100,00)	G(100,00)	**848**			100,00				100,00	
			**849**		100,00				100,00		
			**850**			100,00				100,00	
**284**	V(100,00)	V(100,00)	**851**				100,00				100,00
			**852**	100,00				100,00			
			**853**		100,00				100,00		
**285**	L(100,00)	L(100,00)	**854**				100,00				100,00
			**855**			100,00				100,00	
			**856**	100,00				99,88		0,12	
**286**	T(100,00)	A(0,12) T(99,88)	**857**		100,00				100,00		
			**858**	99,63		0,37		99,75		0,25	
			**859**	100,00				99,45		0,55	
**287**	T(100,00)	A(0,55) T(99,45)	**860**		100,00				100,00		
			**861**		1,30		98,70				100,00
			**862**	100,00				100,00			
**288**	S(100,00)	S(100,00)	**863**			100,00				100,00	
			**864**		100,00				100,00		
			**865**				100,00		0,16		99,84
**289**	C(100,00)	R(0,16) C(99,84)	**866**			100,00				100,00	
			**867**				100,00				100,00
			**868**			100,00				100,00	
**290**	G(100,00)	G(100,00)	**869**			100,00				100,00	
			**870**				100,00				100,00
			**871**	100,00				96,29		3,71	
**291**	N(100,00)	N(95,96) D(3,72) S(0,32)	**872**	100,00				99,68		0,32	
			**873**		98,70		1,30		100,00		
			**874**	100,00				100,00			
**292**	T(100,00)	T(100,00)	**875**		100,00				100,00		
			**876**		100,00				100,00		
			**877**		100,00				100,00		
**293**	L(100,00)	L(100,00)	**878**				100,00				100,00
			**879**		100,00				100,00		
			**880**	100,00				100,00			
**294**	T(100,00)	W(100,00)	**881**		100,00				100,00		
			**882**				100,00				100,00
			**883**				100,00		0,23		99,77
**295**	C(100,00)	R(0,23) C(99,77)	**884**			100,00				100,00	
			**885**		44,68		55,32		100,00		
			**886**				100,00				100,00
**296**	Y(100,00)	C(0,12) Y(99,88)	**887**	100,00				99,88		0,12	
			**888**		100,00				100,00		
			**889**	100,00				99,70		0,30	
**297**	I(100,00)	I(99,70) V(0,30)	**890**				100,00				100,00
			**891**		100,00				100,00		
			**892**	100,00				99,88		0,12	
**298**	K(100,00)	E(0,12) K(99,88)	**893**	100,00				100,00			
			**894**			100,00				100,00	
			**895**			100,00				100,00	
**299**	A(100,00)	A(100,00)	**896**		100,00				100,00		
			**897**		100,00				100,00		
**300**		R(100,00)	**898**		100,00				100,00		

	R(44,99)		899	55,01		44,99				100,00	
	Q(55,01)		900	99,63		0,37		99,86		0,14	
			901			100,00				100,00	
301	A(100,00)	A(99,88) V(0,12)	902		100,00				99,88		0,12
			903	100,00				100,00			
			904			100,00				100,00	
302	A(100,00)	A(100,00)	905		100,00				100,00		
			906		100,00				100,00		
			907				100,00				100,00
303	C(100,00)	C(100,00)	908			100,00				100,00	
			909				100,00		0,16		99,84
			910		100,00				100,00		
304	R(100,00)	R(100,00)	911			100,00				100,00	
			912	100,00				100,00			
			913			100,00				100,00	
305	A(100,00)	A(100,00)	914		100,00				100,00		
			915		100,00				100,00		
			916			100,00				100,00	
306	A(100,00)	A(100,00)	917		100,00				100,00		
			918	100,00				100,00			
			919			100,00				100,00	
307	G(100,00)	G(100,00)	920			100,00				100,00	
			921			100,00				100,00	
			922		100,00				100,00		
308	L(100,00)	L(100,00)	923				100,00				100,00
			924		100,00				100,00		
			925		100,00				100,00		
309	R(100,00)	R(100,00)	926			100,00				100,00	
			927			100,00				100,00	
			928			100,00				100,00	
310	D(100,00)	D(100,00)	929	100,00				100,00			
			930		53,28		46,72		100,00		
			931				100,00		0,18		99,82
311	C(100,00)	R(0,18) C(99,82)	932			100,00				100,00	
			933		100,00				100,00		
			934	100,00				100,00			
312	T(100,00)	I(0,12) T(99,88)	935		100,00				99,88		0,12
			936		100,00				100,00		
			937	100,00				100,00			
313	M(100,00)	M(100,00)	938				100,00				100,00
			939			100,00				100,00	
			940		100,00				100,00		
314	L(100,00)	L(100,00)	941				100,00				100,00
			942		100,00				100,00		
			943			100,00				100,00	
315	V(100,00)	A(0,21) V(99,79)	944				100,00		0,21		99,79
			945			100,00				100,00	
			946				100,00		0,42		99,58
316	C(100,00)	R(0,42) C(99,58)	947			100,00				100,00	
			948				100,00		0,12		99,88
			949			100,00				100,00	
317	G(100,00)	D(0,14) G(99,86)	950			100,00		0,14		99,86	
			951		100,00				100,00		
			952			100,00				100,00	
318	D(100,00)	D(100,00)	953	100,00				100,00			
			954		100,00				99,88		0,12
			955			100,00				100,00	
319	D(100,00)	D(100,00)	956	100,00				100,00			
			957		100,00				100,00		
			958		45,49		54,51		100,00		
320	L(100,00)	L(100,00)	959				100,00				100,00
			960	100,00				100,00			

			**961**			100,00				100,00	
**321**	V(100,00)	V(100,00)	**962**				100,00				100,00
			**963**		100,00				100,00		
			**964**			100,00				100,00	
**322**	V(100,00)	A(0,14)<br>V(99,86)	**965**				100,00		0,14		99,86
			**966**				100,00				100,00
			**967**	100,00				100,00			
**323**	I(100,00)	I(99,82)<br>T(0,18)	**968**				100,00		0,18		99,82
			**969**		100,00				100,00		
			**970**				100,00				100,00
**324**	C(100,00)	C(100,00)	**971**			100,00				100,00	
			**972**		3,89		96,11				100,00
			**973**			100,00				100,00	
**325**	E(100,00)	E(100,00)	**974**	100,00				100,00			
			**975**	100,00				100,00			
			**976**	100,00				100,00			
**326**	S(100,00)	S(100,00)	**977**			100,00				100,00	
			**978**				100,00		0,12		99,88
			**979**			98,83				100,00	
**327**	A(50,12)<br>Y(48,70)<br>X(1,18)	A(100,00)	**980**		50,12		48,70		100,00		
			**981**			98,83				100,00	
			**982**			98,83				100,00	
**328**	G(98,83)<br>X(1,17)	G(100,00)	**983**			98,83				100,00	
			**984**			98,83				100,00	
			**985**			100,00				100,00	
**329**	V(100,00)	D(0,12)<br>V(99,88)	**986**				100,00	0,12			99,88
			**987**		99,26		0,74		100,00		
			**988**		100,00				100,00		
**330**	Q(100,00)	Q(100,00)	**989**	100,00				100,00			
			**990**	45,86		54,14		100,00			
			**991**			100,00				100,00	
**331**	E(100,00)	E(100,00)	**992**	100,00				100,00			
			**993**			100,00				100,00	
			**994**			100,00				100,00	
**332**	D(100,00)	D(100,00)	**995**	100,00				100,00			
			**996**		100,00				100,00		
			**997**			100,00				100,00	
**333**	A(100,00)	A(100,00)	**998**		100,00				100,00		
			**999**	3,89		96,11		0,18		99,82	
			**1000**			100,00				100,00	
**334**	A(100,00)	A(100,00)	**1001**		100,00				100,00		
			**1002**	53,77		46,23		100,00			
