# Supplementary material for: Late Relapse and Reinfection in HCV Patients Treated with Direct-Acting Antiviral (DAA) Drugs
Source: Viruses. 2021 Jun 16;13(6):1151. doi: 10.3390/v13061151 (PMC8235384; doi:10.3390/v13061151)
Supplement: Supplementary file 1 [file viruses-13-01151-s001.zip › Minosse et al_Table S3.pdf]

Table S3. Frequency of substitutions in Pt3 with respect to a GT1a reference sequence (EF407457.1). The relevant amino acid and nucleotide changes between T0 and T1 are written in red.

NS5B aa pos	Pt3 T0 (1a)	Pt3 T1 (1a)	Pt3 T2 (1a)	NS5B nt pos	Pt3 T0 (1a) A	Pt3 T0 (1a) C	Pt3 T0 (1a) G	Pt3 T0 (1a) T	Pt3 T0 (1a) -	Pt3 T1 (1a) A	Pt3 T1 (1a) C	Pt3 T1 (1a) G	Pt3 T1 (1a) T	Pt3 T1 (1a) -	Pt3 T2 (1a) A	Pt3 T2 (1a) C	Pt3 T2 (1a) G	Pt3 T2 (1a) T	Pt3 T2 (1a) -
179	V(100,00)	V(100,00)	A(0,17) V(99,83)	535			100,00					100,00					100,00		
				536				100,00					100,00			0,17		99,83	
				537		100,00							100,00					100,00	
180	S(100,00)	S(100,00)	S(100,00)	538	100,00					100,00					100,00				
				539			100,00					100,00					100,00		
				540		100,00					100,00					100,00			
181	K(100,00)	K(100,00)	R(0,09) E(0,08) K(99,83)	541	100,00					100,00					99,92		0,08		
				542	100,00					100,00					99,91		0,09		
				543			100,00			100,00					100,00				
182	L(100,00)	L(100,00)	L(100,00)	544		100,00					100,00					100,00			
				545				100,00					100,00					100,00	
				546		100,00					100,00					100,00			
183	P(100,00)	P(100,00)	P(100,00)	547		100,00					100,00					100,00			
				548		100,00					100,00					100,00			
				549				100,00			100,00					100,00			
184	A(0,22) V(99,78)	L(100,00)	L(100,00)	550			100,00				100,00					100,00			
				551		0,22		99,78					100,00					100,00	
				552			100,00					100,00					100,00		
185	A(100,00)	A(100,00)	A(100,00)	553			100,00					100,00					100,00		
				554		100,00					100,00					100,00			
				555		100,00					100,00					100,00			
186	V(100,00)	V(100,00)	A(0,13) M(0,03) V(99,84)	556			100,00					100,00			0,03		99,97		
				557				100,00					100,00			0,13		99,87	
				558			100,00					100,00					100,00		
187	M(100,00)	M(100,00)	M(99,97) T(0,07)	559	100,00					100,00					100,00				
				560				100,00					100,00			0,07		99,93	
				561			100,00					100,00					100,00		
188	G(100,00)	G(100,00)	G(100,00)	562			100,00					100,00					100,00		
				563			100,00					100,00					100,00		
				564	100,00					100,00					100,00				
189	S(100,00)	S(100,00)	S(100,00)	565	100,00					100,00					100,00				
				566			100,00					100,00					100,00		
				567		100,00					100,00					100,00			
190	S(100,00)	S(100,00)	S(99,97) T(0,03)	568				100,00					100,00		0,03			99,97	

				569		100,00				100,00				100,00		
				570		100,00				100,00				100,00		
				571				100,00				100,00				100,00
191	Y(100,00)	Y(100,00)	Y(100,00)	572	100,00				100,00				100,00			
				573		99,78		0,22		100,00				99,96		0,04
				574			100,00				100,00				100,00	
192	G(100,00)	G(100,00)	G(100,00)	575			100,00				100,00				100,00	
				576	100,00				100,00				100,00			
				577				100,00				100,00				100,00
193	F(100,00)	F(100,00)	F(100,00)	578				100,00				100,00				100,00
				579		100,00				26,96		73,04		100,00		
				580		100,00				100,00				100,00		
194	Q(100,00)	Q(100,00)	Q(100,00)	581	100,00				100,00				100,00			
				582	100,00				97,59		2,41		100,00			
				583				100,00				100,00				100,00
195	Y(100,00)	Y(100,00)	C(0,03) Y(99,97)	584	100,00				100,00				99,97		0,03	
				585		99,82		0,18		100,00				99,96		0,04
				586				100,00				100,00		0,24	0,34	99,42
196	S(100,00)	S(100,00)	A(0,34) P(0,24) S(99,42)	587		100,00				100,00				100,00		
				588	100,00				81,51		18,49		99,89		0,11	
				589		100,00				100,00				100,00		
197	P(100,00)	P(100,00)	L(0,03) P(99,97)	590		100,00				100,00				99,97		0,03
				591	100,00				100,00				100,00			
				592			100,00				100,00				100,00	
198	G(100,00)	G(100,00)	G(100,00)	593			100,00				100,00				100,00	
				594	100,00				100,00				100,00			
				595		100,00				100,00				100,00		
199	Q(100,00)	Q(100,00)	R(0,09) Q(99,91)	596	100,00				100,00				99,91		0,09	
				597			100,00		0,59		99,41				100,00	
				598		100,00				100,00				99,89		0,11
200	R(100,00)	R(100,00)	R(99,89) W(0,11)	599			100,00				100,00				100,00	
				600			100,00				100,00		0,04		99,96	
				601			100,00				100,00				100,00	
201	V(100,00)	V(100,00)	A(0,14) V(99,86)	602				100,00				100,00		0,14		99,86
				603				100,00				100,00		0,10		99,90
				604			100,00				100,00				100,00	
202	E(100,00)	E(100,00)	E(100,00)	605	100,00				100,00				100,00			
				606	100,00				100,00				99,40		0,60	
				607				100,00				100,00		0,12		99,88
203	F(100,00)	F(100,00)	L(0,12) F(99,88)	608				100,00				100,00				100,00
				609		100,00				100,00				100,00		
204	L(100,00)	L(100,00)	L(100,00)	610		100,00				100,00				100,00		

				**611**				100,00				100,00				100,00
				**612**		100,00				100,00				100,00		
				**613**			100,00				100,00		0,06		99,94	
**205**	V(100,00)	V(100,00)	A(0,05) M(0,06) V(99,89)	**614**				100,00				100,00		0,05		99,95
				**615**		10,17	89,83				100,00				100,00	
				**616**		100,00				100,00				100,00		
**206**	R(100,00)	Q(100,00)	Q(100,00)	**617**			100,00		100,00				100,00			
				**618**	99,63		0,37		100,00				100,00			
				**619**			100,00				100,00				100,00	
**207**	A(99,82) V(0,18)	A(100,00)	A(100,00)	**620**		99,82		0,18		100,00				100,00		
				**621**			100,00				100,00		0,08		99,92	
				**622**				100,00				100,00		0,05		99,95
**208**	W(100,00)	W(100,00)	R(0,05) W(99,92) *(0,03)	**623**			100,00				100,00		0,03		99,97	
				**624**			100,00				100,00				100,00	
				**625**	100,00				100,00				100,00			
**209**	K(100,00)	K(100,00)	K(100,00)	**626**	100,00				100,00				100,00			
				**627**	88,08		11,92				100,00				100,00	
				**628**				100,00				100,00				100,00
**210**	S(100,00)	S(100,00)	S(100,00)	**629**		100,00				100,00				100,00		
				**630**		11,92		88,08		100,00				100,00		
				**631**	100,00				100,00				100,00			
**211**	K(100,00)	K(100,00)	K(100,00)	**632**	100,00				100,00				100,00			
				**633**			100,00		49,68		50,32				100,00	
				**634**	100,00				100,00				100,00			
**212**	K(99,78) T(0,22)	R(100,00)	R(100,00)	**635**	99,78	0,22					100,00				100,00	
				**636**			100,00				100,00				100,00	
				**637**	99,78		0,22		100,00				100,00			
**213**	A(0,22) T(99,78)	T(100,00)	T(100,00)	**638**		100,00				100,00				100,00		
				**639**		100,00				100,00				100,00		
				**640**		100,00				100,00				100,00		
**214**	P(100,00)	P(100,00)	P(100,00)	**641**		100,00				100,00				100,00		
				**642**	98,68		1,32		99,73		0,27		100,00			
				**643**	100,00				100,00				99,62		0,38	
**215**	M(100,00)	M(100,00)	M(99,62) V(0,38)	**644**				100,00				100,00				100,00
				**645**			100,00				100,00				100,00	
				**646**			100,00				100,00				100,00	
**216**	G(100,00)	G(100,00)	G(100,00)	**647**			100,00				100,00				100,00	
				**648**			100,00				100,00				100,00	
				**649**				100,00				100,00		0,08		99,92
**217**	F(100,00)	F(100,00)	L(0,08) F(99,92)	**650**				100,00				100,00				100,00
				**651**				100,00		100,00				100,00		
**218**	S(100,00)	S(100,00)	S(100,00)	**652**				100,00				100,00				100,00

				**653**		100,00				100,00				100,00		
				**654**	99,49		0,51				100,00		0,29		99,71	
				**655**				100,00				100,00		0,07		99,93
**219**	Y(100,00)	Y(100,00)	H(0,07)<br>Y(99,93)	**656**	100,00				100,00				100,00			
				**657**		0,22		99,78				100,00		0,07		99,93
				**658**			100,00				100,00		0,03		99,97	
**220**	D(100,00)	D(100,00)	N(0,03)<br>D(99,88)<br>G(0,09)	**659**	100,00				100,00				99,91		0,09	
				**660**		0,80		99,20				100,00		41,62		58,38
				**661**	99,78		0,22		100,00				99,86		0,14	
**221**	A(0,22)<br>T(99,78)	T(100,00)	A(0,14)<br>T(99,86)	**662**		100,00				100,00				100,00		
				**663**		99,41		0,59		100,00				100,00		
				**664**		100,00				100,00				100,00		
**222**	R(100,00)	R(100,00)	R(99,95)<br>H(0,05)	**665**			100,00				100,00		0,05		99,95	
				**666**		99,78		0,22		100,00				100,00		
				**667**				100,00				100,00				100,00
**223**	C(99,82)<br>*(0,18)	C(100,00)	C(100,00)	**668**			100,00				100,00				100,00	
				**669**	0,18	99,82						100,00				100,00
				**670**				100,00				100,00				100,00
**224**	F(100,00)	F(100,00)	F(100,00)	**671**				100,00				100,00				100,00
				**672**				100,00				100,00				100,00
				**673**			100,00				100,00				100,00	
**225**	D(100,00)	D(100,00)	D(99,83)<br>G(0,17)	**674**	100,00				100,00				99,83		0,17	
				**675**		99,49		0,51		100,00				100,00		
				**676**				100,00				100,00				100,00
**226**	S(100,00)	S(100,00)	S(100,00)	**677**		100,00				100,00				100,00		
				**678**		100,00				100,00				100,00		
				**679**	100,00				100,00				99,86		0,14	
**227**	T(100,00)	T(100,00)	A(0,14)<br>T(99,86)	**680**		100,00				100,00				100,00		
				**681**	99,74		0,26		100,00				99,38		0,07	0,55
				**682**			100,00				100,00				100,00	
**228**	A(0,18)<br>V(99,82)	V(100,00)	A(0,15)<br>V(99,85)	**683**		0,18		99,82				100,00		0,15		99,85
				**684**		100,00				100,00				100,00		
				**685**	100,00				100,00				99,87		0,13	
**229**	T(100,00)	T(100,00)	A(0,13)<br>T(99,87)	**686**		100,00				100,00				100,00		
				**687**		0,33		99,67				100,00	0,03	0,07		99,91
				**688**			99,16				100,00		0,04		99,75	
**230**	E(99,16)<br>X(0,84)	E(100,00)	E(99,70)<br>K(0,03) V(0,05)<br>X(0,22)	**689**	99,16				100,00				99,73			0,05
				**690**			100,00				100,00				100,00	
				**691**	100,00				100,00				100,00			
**231**	S(100,00)	P(100,00)	S(100,00)	**692**			100,00				100,00				100,00	
				**693**		1,68		98,32		100,00				99,96		0,04
**232**		D(100,00)	D(99,83)G(0,17)	**694**			100,00				100,00				100,00	

	D(99,78) G(0,22)			**695**	99,78		0,22		100,00				99,83		0,17	
				**696**		99,52		0,48		100,00				99,58		0,42
				**697**	100,00				100,00				99,93		0,07	
**233**	I(100,00)	I(100,00)	I(99,93) V(0,07)	**698**				100,00				100,00				100,00
				**699**		100,00				100,00				100,00		
				**700**		100,00				100,00				99,95		0,05
**234**	R(100,00)	R(100,00)	R(99,92) C(0,05) H(0,03)	**701**			100,00				100,00		0,03		99,97	
				**702**				100,00				100,00				100,00
				**703**	100,00				100,00				100,00			
**235**	T(100,00)	T(100,00)	T(100,00)	**704**		100,00				100,00				100,00		
				**705**			100,00				100,00		39,78		60,22	
				**706**			100,00				100,00				100,00	
**236**	E(100,00)	E(100,00)	E(100,00)	**707**	100,00				100,00				100,00			
				**708**			100,00				100,00				100,00	
				**709**			100,00				100,00				100,00	
**237**	E(100,00)	E(100,00)	E(100,00)	**710**	100,00				100,00				100,00			
				**711**			100,00				100,00				100,00	
				**712**			100,00				100,00				99,95	0,05
**238**	A(100,00)	A(100,00)	A(99,95) S(0,05)	**713**		100,00				100,00				100,00		
				**714**	100,00				100,00				99,76		0,24	
				**715**	100,00				100,00				99,86		0,14	
**239**	I(100,00)	I(100,00)	I(99,79) M(0,04) T(0,03) V(0,14)	**716**				100,00				100,00		0,03		99,97
				**717**		100,00			100,00				99,96		0,04	
				**718**		0,40		99,60				100,00		0,14		99,86
**240**	H(0,40) Y(99,60)	Y(100,00)	C(0,09) H(0,14) Y(99,77)	**719**	100,00				100,00				99,91		0,09	
				**720**		100,00				100,00				99,42		0,58
				**721**		100,00				100,00				100,00		
**241**	Q(100,00)	Q(100,00)	Q(100,00)	**722**	100,00				100,00				100,00			
				**723**	10,57		89,43		100,00				100,00			
				**724**				100,00				100,00		0,31		99,69
**242**	C(100,00)	C(100,00)	R(0,31) C(99,69)	**725**			100,00				100,00				100,00	
				**726**				100,00				100,00				100,00
				**727**				100,00				100,00		0,07		99,93
**243**	C(100,00)	C(100,00)	R(0,07)C(99,93)	**728**			100,00				100,00				100,00	
				**729**				100,00				100,00		0,11		99,89
				**730**			100,00				100,00				100,00	
**244**	D(99,78) G(0,22)	D(100,00)	D(99,85) G(0,15)	**731**	99,78		0,22		100,00				99,85		0,15	
				**732**		100,00				100,00				100,00		
				**733**		100,00				100,00				100,00		
**245**	L(100,00)	L(100,00)	L(100,00)	**734**				100,00				100,00				100,00
				**735**			100,00				100,00				100,00	
**246**	D(100,00)	D(100,00)	D(100,00)	**736**			100,00				100,00				100,00	

				**737**	100,00				100,00				100,00			
				**738**		100,00				100,00				100,00		
				**739**		100,00				100,00				100,00		
**247**	P(100,00)	P(100,00)	P(100,00)	**740**		100,00				100,00				100,00		
				**741**		100,00				100,00				100,00		
				**742**		100,00				100,00				100,00		
**248**	R(0,26) Q(99,74)	Q(100,00)	R(0,07) Q(99,93)	**743**	99,74		0,26		100,00				99,93		0,07	
				**744**	100,00				100,00				100,00			
				**745**			100,00				100,00				100,00	
**249**	A(100,00)	A(100,00)	A(100,00)	**746**		100,00				100,00				100,00		
				**747**		100,00				100,00				100,00		
				**748**		100,00				100,00				100,00		
**250**	R(100,00)	R(100,00)	R(99,97) H(0,03)	**749**			100,00				100,00		0,03		99,97	
				**750**		100,00				99,68		0,32		99,97		0,03
				**751**			100,00				100,00		0,38		99,62	
**251**	V(100,00)	V(100,00)	A(0,05) M(0,38) V(99,57)	**752**				100,00				100,00		0,05		99,95
				**753**	0,26		99,74				100,00		0,04		99,96	
				**754**			100,00				100,00				100,00	
**252**	A(99,78) V(0,22)	A(100,00)	A(100,00)	**755**		99,78		0,22		100,00				100,00		
				**756**		100,00				100,00				100,00		
				**757**	100,00				100,00				99,91		0,09	
**253**	I(100,00)	I(100,00)	I(99,57) T(0,33) V(0,10)	**758**				100,00				100,00		0,33		99,67
				**759**		100,00				100,00				100,00		
				**760**	100,00				100,00				99,97		0,03	
**254**	K(100,00)	K(100,00)	E(0,03) K(99,45) T(0,52)	**761**	100,00				100,00				99,48	0,52		
				**762**			100,00				100,00				100,00	
				**763**				100,00				100,00				100,00
**255**	S(100,00)	S(100,00)	S(100,00)	**764**		100,00				100,00				100,00		
				**765**		99,63		0,37		100,00				100,00		
				**766**		100,00				100,00				100,00		
**256**	L(100,00)	L(100,00)	L(100,00)	**767**				100,00				100,00				100,00
				**768**		100,00				100,00				99,97		0,03
				**769**	100,00				100,00				100,00			
**257**	T(100,00)	T(100,00)	I(0,05) T(99,95)	**770**		100,00				100,00				99,95		0,05
				**771**		99,63		0,37				100,00		0,03		99,97
				**772**			100,00				100,00				99,54	
**258**	E(100,00)	E(100,00)	E(99,54) X(0,46)	**773**	100,00				100,00				99,54			
				**774**			100,00				100,00				100,00	
				**775**	100,00				100,00				100,00			
**259**	R(100,00)	R(100,00)	R(100,00)	**776**			100,00				100,00				100,00	
				**777**			100,00		48,23		51,77		99,86		0,14	
**260**	L(100,00)	L(100,00)	L(100,00)	**778**		100,00				100,00				100,00		

				**779**				100,00				100,00				100,00
				**780**			0,22	99,78		0,32		99,68				100,00
				**781**				100,00				100,00				100,00
**261**	Y(100,00)	Y(100,00)	Y(100,00)	**782**	100,00				100,00				100,00			
				**783**				100,00				100,00		0,07		99,93
				**784**			100,00				100,00				100,00	
**262**	V(100,00)	V(100,00)	A(0,09)<br>V(99,91)	**785**				100,00				100,00		0,09		99,91
				**786**		10,75		89,25		95,44		4,56		100,00		
				**787**			100,00				100,00				100,00	
**263**	G(100,00)	G(100,00)	G(100,00)	**788**			100,00				100,00				100,00	
				**789**			100,00				100,00				100,00	
				**790**			100,00				100,00				100,00	
**264**	G(100,00)	G(100,00)	G(100,00)	**791**			100,00				100,00				100,00	
				**792**		100,00				99,68		0,32		100,00		
				**793**		100,00				100,00				100,00		
**265**	P(100,00)	P(100,00)	P(100,00)	**794**		100,00				100,00				100,00		
				**795**				100,00		24,28		75,72				100,00
				**796**		100,00				100,00				100,00		
**266**	L(100,00)	L(100,00)	L(100,00)	**797**				100,00				100,00				100,00
				**798**				100,00				100,00		0,03		99,97
				**799**	100,00				100,00				99,77		0,23	
**267**	T(100,00)	T(100,00)	A(0,23)<br>T(99,77)	**800**		100,00				100,00				100,00		
				**801**		100,00				100,00				100,00		
				**802**	100,00				100,00				100,00			
**268**	N(100,00)	N(100,00)	N(99,94)<br>S(0,06)	**803**	100,00				100,00				99,94		0,06	
				**804**		2,71		97,29				100,00		0,07		99,93
				**805**				100,00				100,00				100,00
**269**	S(100,00)	S(100,00)	S(100,00)	**806**		100,00				100,00				100,00		
				**807**	99,56		0,44		100,00				100,00			
				**808**	100,00				100,00				100,00			
**270**	R(100,00)	R(100,00)	R(100,00)	**809**			100,00				100,00				100,00	
				**810**			100,00		24,28		75,72				100,00	
				**811**			100,00				100,00				100,00	
**271**	G(100,00)	G(100,00)	G(100,00)	**812**			100,00				100,00				100,00	
				**813**			100,00				100,00				100,00	
				**814**			100,00				100,00				100,00	
**272**	E(100,00)	E(100,00)	E(100,00)	**815**	100,00				100,00				100,00			
				**816**	100,00				0,32		99,68				100,00	
				**817**	100,00				100,00				100,00			
**273**	N(100,00)	N(99,68)<br>S(0,32)	N(99,81)<br>S(0,19)	**818**	100,00				99,68		0,32		99,81		0,19	
				**819**		100,00				100,00				100,00		
**274**		C(100,00)		**820**				100,00				100,00		0,06		99,94

	C(99,63) Y(0,37)		R(0,06) C(99,94)	**821**	0,37		99,63				100,00				100,00	
				**822**		100,00				100,00				99,34		0,66
**275**	G(100,00)	G(100,00)	D(0,52) G(99,48)	**823**			100,00				100,00				100,00	
				**824**			100,00				100,00		0,52		99,48	
				**825**		100,00				100,00				100,00		
**276**	Y(100,00)	Y(100,00)	Y(100,00)	**826**				100,00				100,00				100,00
				**827**	100,00				100,00				100,00			
				**828**		0,37		99,63		99,73		0,27		100,00		
**277**	R(100,00)	R(100,00)	R(99,88) C(0,08) H(0,04)	**829**		100,00				100,00				99,92		0,08
				**830**			100,00				100,00		0,04		99,96	
				**831**		100,00				100,00				100,00		
**278**	R(100,00)	R(100,00)	R(100,00)	**832**	100,00				100,00				100,00			
				**833**			100,00				100,00				100,00	
				**834**	0,48		99,52				100,00		0,03		99,97	
**279**	C(100,00)	C(100,00)	R(0,09) C(99,88) Y(0,03)	**835**				100,00				100,00		0,09		99,91
				**836**			100,00				100,00		0,04		99,96	
				**837**		100,00				100,00				100,00		
**280**	R(99,82) C(0,18)	R(100,00)	R(99,91) C(0,04) H(0,05)	**838**		99,82		0,18		100,00				99,96		0,04
				**839**			100,00				100,00		0,05		99,95	
				**840**		100,00				100,00				100,00		
**281**	A(100,00)	A(100,00)	A(100,00)	**841**			100,00				100,00				100,00	
				**842**		100,00				100,00				100,00		
				**843**	0,29		99,71				100,00		0,04		99,96	
**282**	S(100,00)	S(100,00)	G(0,04) S(99,96)	**844**	100,00				100,00				99,96		0,04	
				**845**			100,00				100,00				100,00	
				**846**		12,69		87,31		100,00				99,95		0,05
**283**	D(0,22) G(99,78)	G(100,00)	G(100,00)	**847**			100,00				100,00				100,00	
				**848**	0,22		99,78				100,00				100,00	
				**849**		100,00				100,00				99,95		0,05
**284**	V(100,00)	V(100,00)	V(100,00)	**850**			100,00				100,00				100,00	
				**851**				100,00				100,00				100,00
				**852**	99,82		0,18		62,65			37,35	99,97		0,03	
**285**	L(100,00)	L(100,00)	L(99,95) P(0,05)	**853**		100,00				100,00				99,71		0,29
				**854**				100,00				100,00		0,05		99,95
				**855**	0,69		99,31				100,00		0,04		99,96	
**286**	T(100,00)	T(100,00)	A(0,11) T(99,89)	**856**	100,00				100,00				99,89		0,11	
				**857**		100,00				100,00				100,00		
				**858**	12,44		87,56		100,00				100,00			
**287**	T(100,00)	T(100,00)	A(0,09) T(99,91)	**859**	100,00				100,00				99,91		0,09	
				**860**		100,00				100,00				100,00		
				**861**		0,44		99,56				100,00				100,00
**288**	S(100,00)	S(100,00)		**862**	100,00				100,00				99,93		0,07	

			G(0,07) S(99,93)	**863**			100,00				100,00				100,00	
				**864**		100,00				100,00				100,00		
				**865**				100,00				100,00		0,14		99,86
**289**	C(100,00)	C(100,00)	R(0,14) C(99,86)	**866**			100,00				100,00				100,00	
				**867**		10,39		89,61		100,00				99,91		0,09
				**868**			100,00				100,00				100,00	
**290**	G(100,00)	G(100,00)	G(100,00)	**869**			100,00				100,00				100,00	
				**870**		0,40		99,60		3,48		96,52		0,04		99,96
				**871**	100,00				100,00				99,90		0,10	
**291**	N(100,00)	N(100,00)	N(99,68) D(0,10) S(0,22)	**872**	100,00				100,00				99,78		0,22	
				**873**		99,67		0,33		100,00				99,96		0,04
				**874**	100,00				100,00				100,00			
**292**	T(100,00)	T(100,00)	T(100,00)	**875**		100,00				100,00				100,00		
				**876**		100,00				100,00				100,00		
				**877**		100,00				100,00				100,00		
**293**	L(100,00)	L(100,00)	L(100,00)	**878**				100,00				100,00				100,00
				**879**		100,00				100,00				100,00		
				**880**	100,00				100,00				99,92		0,08	
**294**	T(100,00)	T(100,00)	A(0,08) T(99,92)	**881**		100,00				100,00				100,00		
				**882**				100,00				100,00				100,00
				**883**				100,00				100,00		0,15		99,85
**295**	C(100,00)	C(100,00)	R(0,15) C(99,81) Y(0,04)	**884**			100,00				100,00		0,04		99,96	
				**885**		87,82		12,18		100,00				99,69		0,31
				**886**				100,00				100,00		0,15		99,85
**296**	Y(100,00)	Y(100,00)	C(0,11) H(0,15) Y(99,74)	**887**	100,00				100,00				99,89		0,11	
				**888**		99,82		0,18		100,00				99,74		0,26
				**889**	99,82		0,18		100,00				99,90		0,10	
**297**	I(99,82) V(0,18)	I(100,00)	I(99,75) T(0,15) V(0,10)	**890**				100,00				100,00		0,15		99,85
				**891**		100,00				100,00				100,00		
				**892**	100,00				100,00				99,88		0,12	
**298**	K(100,00)	K(100,00)	E(0,12) K(99,88)	**893**	100,00				100,00				100,00			
				**894**			100,00				100,00				100,00	
				**895**			100,00				100,00				100,00	
**299**	A(100,00)	A(100,00)	A(100,00)	**896**		100,00				100,00				100,00		
				**897**		100,00				100,00				99,93		0,07
				**898**		100,00				100,00				100,00		
**300**	Q(100,00)	R(100,00)	R(100,00)	**899**	100,00						100,00				100,00	
				**900**	100,00				100,00				99,96		0,04	
				**901**			100,00				100,00				100,00	
**301**	A(100,00)	A(100,00)	A(100,00)	**902**		100,00				100,00				100,00		
				**903**	100,00				100,00				99,91		0,09	
**302**	A(100,00)	A(100,00)	A(100,00)	**904**			100,00				100,00				100,00	

				**905**		100,00				100,00				100,00		
				**906**		100,00				100,00				100,00		
				**907**				100,00				100,00				100,00
**303**	C(100,00)	C(100,00)	C(100,00)	**908**			100,00				100,00				100,00	
				**909**		0,26		99,74				100,00		0,05		99,95
				**910**		100,00				100,00				99,96		0,04
**304**	R(100,00)	R(100,00)	R(99,96) *(0,04)	**911**			100,00				100,00				100,00	
				**912**	100,00				100,00				100,00			
				**913**			100,00				100,00				100,00	
**305**	A(100,00)	A(100,00)	A(99,96) V(0,04)	**914**		100,00				100,00				99,96		0,04
				**915**		100,00				100,00				99,10		0,90
				**916**			100,00				100,00		0,05		99,95	
**306**	A(99,82) V(0,18)	A(100,00)	A(99,91) T(0,05) V(0,04)	**917**		99,82		0,18		100,00				99,96		0,04
				**918**	100,00				100,00				100,00			
				**919**			100,00				100,00				100,00	
**307**	G(100,00)	G(100,00)	G(100,00)	**920**			100,00				100,00				100,00	
				**921**			100,00				100,00				100,00	
				**922**		99,78		0,22		100,00				100,00		
**308**	L(99,78) F(0,22)	L(100,00)	L(100,00)	**923**				100,00				100,00				100,00
				**924**		100,00				100,00				100,00		
				**925**		100,00				100,00				100,00		
**309**	R(100,00)	Q(100,00)	R(0,16) Q(99,84)	**926**			100,00		100,00				99,84		0,16	
				**927**			100,00				100,00				100,00	
				**928**			100,00				100,00				100,00	
**310**	D(100,00)	D(100,00)	D(100,00)	**929**	100,00				100,00				100,00			
				**930**		100,00				100,00				100,00		
				**931**				100,00				100,00		0,09		99,91
**311**	C(100,00)	C(100,00)	R(0,09) C(99,91)	**932**			100,00				100,00				100,00	
				**933**		100,00				100,00				99,61		0,39
				**934**	99,82		0,18		100,00				99,90		0,10	
**312**	A(0,18) T(99,82)	T(100,00)	A(0,10) I(0,07) T(99,83)	**935**		100,00				100,00				99,93		0,07
				**936**		100,00				100,00				99,96		0,04
				**937**	100,00				100,00				100,00			
**313**	I(0,18) M(99,82)	M(100,00)	I(0,03) M(99,91) T(0,06)	**938**				100,00				100,00		0,06		99,94
				**939**	0,18		99,82				100,00		0,03		99,97	
				**940**		100,00				100,00				99,61		0,39
**314**	L(100,00)	L(100,00)	L(99,51) F(0,39) P(0,10)	**941**				100,00				100,00		0,10		99,90
				**942**		99,60		0,40		100,00				100,00		
				**943**			100,00				100,00				100,00	
**315**	V(100,00)	V(100,00)	A(0,20) V(99,80)	**944**				100,00				100,00		0,20		99,80
				**945**			100,00				100,00		0,55		99,45	
**316**	C(100,00)	C(100,00)		**946**				100,00				100,00		0,21		99,79

			R(0,21) C(99,79)	**947**			100,00				100,00				100,00	
				**948**				100,00				100,00		0,07		99,93
				**949**			100,00				100,00		0,70		99,30	
**317**	G(100,00)	G(100,00)	G(99,30) S(0,70)	**950**			100,00				100,00				100,00	
				**951**		99,82		0,18		100,00				99,96		0,04
				**952**			100,00				100,00				100,00	
**318**	D(100,00)	D(100,00)	D(99,92) G(0,08)	**953**	100,00				100,00				99,92		0,08	
				**954**		100,00				100,00				99,93		0,07
				**955**			100,00				100,00				100,00	
**319**	D(100,00)	D(100,00)	D(100,00)	**956**	100,00				100,00				100,00			
				**957**		100,00				100,00				99,97		0,03
				**958**				100,00		59,81		40,19		100,00		
**320**	L(100,00)	L(100,00)	L(100,00)	**959**				100,00				100,00				100,00
				**960**	100,00				100,00				99,95		0,05	
				**961**			100,00				100,00		0,37		99,63	
**321**	V(100,00)	V(100,00)	I(0,37) V(99,63)	**962**				100,00				100,00				100,00
				**963**		100,00				100,00				100,00		
				**964**			100,00				100,00				100,00	
**322**	V(100,00)	V(100,00)	A(0,09) V(99,91)	**965**				100,00				100,00		0,09		99,91
				**966**				100,00				100,00		0,05		99,95
				**967**	100,00				100,00				99,93		0,07	
**323**	I(100,00)	I(100,00)	I(99,80) T(0,14) V(0,06)	**968**				100,00				100,00		0,14		99,86
				**969**		100,00				100,00				100,00		
				**970**		0,18		99,82				100,00		0,21		99,79
**324**	R(0,18) C(99,82)	C(100,00)	R(0,21) C(99,75) Y(0,04)	**971**			100,00				100,00		0,04		99,96	
				**972**				100,00		0,70		99,30		0,21		99,79
				**973**			100,00				100,00				100,00	
**325**	E(100,00)	E(100,00)	E(100,00)	**974**	100,00				100,00				100,00			
				**975**	99,23		0,77		100,00				100,00			
				**976**	100,00				100,00				100,00			
**326**	N(0,29) S(99,71)	S(100,00)	S(100,00)	**977**	0,29		99,71				100,00				100,00	
				**978**		99,45		0,55				100,00		0,15		99,85
				**979**		100,00					100,00		0,03		99,97	
**327**	Q(100,00)	A(100,00)	A(99,93) T(0,03) V(0,04)	**980**	100,00					100,00				99,96		0,04
				**981**			100,00				100,00				100,00	
				**982**			100,00				100,00				100,00	
**328**	G(100,00)	G(100,00)	G(100,00)	**983**			100,00				100,00				100,00	
				**984**	100,00						100,00				100,00	
				**985**			100,00				100,00				100,00	
**329**	V(100,00)	V(100,00)	A(0,29) V(99,71)	**986**				100,00				100,00		0,29		99,71
				**987**		100,00						100,00				100,00
**330**	Q(100,00)	Q(100,00)	Q(100,00)	**988**		100,00				100,00				100,00		

				**989**	100,00				100,00				100,00			
				**990**			100,00				100,00				100,00	
				**991**			100,00				100,00				100,00	
**331**	E(100,00)	E(100,00)	E(100,00)	**992**	100,00				100,00				100,00			
				**993**			100,00				100,00				100,00	
				**994**			100,00				100,00				100,00	
**332**	D(100,00)	D(100,00)	D(100,00)	**995**	100,00				100,00				100,00			
				**996**				100,00		100,00				99,76		0,24
				**997**			100,00				100,00		0,05		99,95	
**333**	A(100,00)	A(100,00)	A(99,88) T(0,05) V(0,07)	**998**		100,00				100,00				99,93		0,07
				**999**	99,27		0,73				100,00		0,03		99,97	
				**1000**			100,00				100,00				100,00	
**334**	A(100,00)	A(100,00)	A(99,64) G(0,31) V(0,04)	**1001**		100,00				100,00				99,64	0,31	0,04
				**1002**	0,37		99,63				100,00		0,21		99,79	
