# Supplementary material for: Late Relapse and Reinfection in HCV Patients Treated with Direct-Acting Antiviral (DAA) Drugs
Source: Viruses. 2021 Jun 16;13(6):1151. doi: 10.3390/v13061151 (PMC8235384; doi:10.3390/v13061151)
Supplement: Supplementary file 1 [file viruses-13-01151-s001.zip › Minosse et al_Table S4.pdf]

Table S4. Frequency of substitutions in Pt4 with respect to a GT1b reference sequence (EU781827.1). The relevant amino acid and nucleotide changes between T0 and T1 are written in red.

NS5B aa position	Pt4 T0 (1b)	Pt4 T1 (1b)	NS5B nt position	Pt4 T0 (1b) A	C	G	T	-	Pt4 T1 (1b) A	C	G	T	-
			**535**			100,00					100,00		
**179**	V(100,00)	V(100,00)	**536**				100,00					100,00	
			**537**		100,00					100,00			
			**538**				100,00					100,00	
**180**	S(100,00)	S(100,00)	**539**		100,00					100,00			
			**540**		100,00					100,00			
			**541**	100,00					100,00				
**181**	T(100,00)	N(100,00)	**542**		100,00				100,00				
			**543**		100,00					100,00			
			**544**		100,00					100,00			
**182**	L(100,00)	L(100,00)	**545**				100,00					100,00	
			**546**		100,00					100,00			
			**547**		100,00					100,00			
**183**	P(100,00)	P(100,00)	**548**		100,00					100,00			
			**549**		97,62		2,38			100,00			
			**550**		100,00					100,00			
**184**	Q(100,00)	Q(100,00)	**551**	100,00					100,00				
			**552**			100,00					100,00		
			**553**			100,00					100,00		
**185**	A(100,00)	A(100,00)	**554**		100,00					100,00			
			**555**		100,00					100,00			
			**556**			100,00					100,00		
**186**	V(100,00)	V(100,00)	**557**				100,00					100,00	
			**558**			100,00					100,00		
			**559**	100,00					100,00				
**187**	M(100,00)	M(100,00)	**560**				100,00					100,00	
			**561**			100,00					100,00		
			**562**			100,00					100,00		
**188**	G(100,00)	G(100,00)	**563**			100,00					100,00		
			**564**		100,00					100,00			
			**565**				100,00					100,00	
**189**	S(100,00)	S(100,00)	**566**		100,00					100,00			
			**567**		100,00					100,00			
			**568**				100,00					100,00	
**190**	S(100,00)	S(100,00)	**569**		100,00					100,00			
			**570**	100,00					100,00				
			**571**				100,00					100,00	
**191**	Y(100,00)	Y(100,00)	**572**	100,00					100,00				
			**573**		100,00					100,00			
			**574**			100,00					100,00		
**192**	G(100,00)	G(100,00)	**575**			100,00					100,00		
			**576**	3,65		96,35					100,00		
			**577**				100,00					100,00	
**193**	F(100,00)	F(100,00)	**578**				100,00					100,00	
			**579**		100,00					100,00			
			**580**		100,00					100,00			
**194**	Q(100,00)	Q(100,00)	**581**	100,00					100,00				
			**582**	100,00					100,00				
			**583**				100,00					100,00	
**195**	Y(100,00)	Y(100,00)	**584**	100,00					100,00				
			**585**		100,00					100,00			
			**586**				100,00					100,00	
**196**	S(100,00)	S(100,00)	**587**		100,00					100,00			
			**588**				100,00					100,00	
**197**	P(100,00)	P(100,00)	**589**		100,00					100,00			

			**590**		100,00				100,00		
			**591**				100,00				100,00
			**592**			100,00				100,00	
**198**	G(100,00)	G(100,00)	**593**			100,00				100,00	
			**594**	100,00				100,00			
			**595**		100,00				100,00		
**199**	Q(100,00)	Q(100,00)	**596**	100,00				100,00			
			**597**			100,00				100,00	
			**598**		100,00				100,00		
**200**	R(100,00)	R(100,00)	**599**			100,00				100,00	
			**600**	0,98		99,02				100,00	
			**601**			100,00				100,00	
**201**	V(100,00)	V(100,00)	**602**				100,00				100,00
			**603**				100,00				100,00
			**604**			100,00				100,00	
**202**	E(100,00)	E(100,00)	**605**	100,00				100,00			
			**606**	1,68		98,32				100,00	
			**607**				100,00				100,00
**203**	F(100,00)	F(100,00)	**608**				100,00				100,00
			**609**		100,00						100,00
			**610**		100,00				100,00		
**204**	L(100,00)	L(100,00)	**611**				100,00				100,00
			**612**			100,00		1,40		98,60	
			**613**			100,00				100,00	
**205**	V(100,00)	V(100,00)	**614**				100,00				100,00
			**615**			100,00				100,00	
			**616**	100,00				100,00			
**206**	N(100,00)	N(100,00)	**617**	100,00				100,00			
			**618**		2,81		97,19				100,00
			**619**			100,00				100,00	
**207**	A(100,00)	A(100,00)	**620**		100,00				100,00		
			**621**		100,00				100,00		
			**622**				100,00				100,00
**208**	W(100,00)	W(100,00)	**623**			100,00				100,00	
			**624**			100,00				100,00	
			**625**	100,00				100,00			
**209**	K(100,00)	K(100,00)	**626**	100,00				100,00			
			**627**	100,00				100,00			
			**628**				100,00				100,00
**210**	S(100,00)	S(100,00)	**629**		100,00				100,00		
			**630**	100,00				100,00			
			**631**	100,00				100,00			
**211**	K(100,00)	K(100,00)	**632**	100,00				100,00			
			**633**			100,00				100,00	
			**634**	100,00				100,00			
**212**	K(100,00)	K(100,00)	**635**	100,00				100,00			
			**636**	100,00				100,00			
			**637**				100,00				100,00
**213**	C(100,00)	C(100,00)	**638**			100,00				100,00	
			**639**		100,00				100,00		
			**640**		100,00				100,00		
**214**	P(100,00)	P(100,00)	**641**		100,00				100,00		
			**642**		99,02		0,98		100,00		
			**643**	100,00				100,00			
**215**	M(100,00)	M(100,00)	**644**				100,00				100,00
			**645**			100,00				100,00	
			**646**			100,00				100,00	
**216**	G(100,00)	G(100,00)	**647**			100,00				100,00	
			**648**		100,00				100,00		
			**649**				100,00				100,00
**217**	F(100,00)	F(100,00)	**650**				100,00				100,00
			**651**				100,00				100,00

			**652**			100,00				100,00	
**218**	A(100,00)	A(100,00)	**653**		100,00				100,00		
			**654**	100,00				99,79		0,21	
			**655**				100,00				100,00
**219**	Y(100,00)	Y(100,00)	**656**	100,00				100,00			
			**657**				100,00				100,00
			**658**			100,00				100,00	
**220**	D(100,00)	D(100,00)	**659**	100,00				100,00			
			**660**		2,24		97,76				100,00
			**661**	100,00				100,00			
**221**	T(100,00)	T(100,00)	**662**		100,00				100,00		
			**663**		100,00				100,00		
			**664**		100,00				100,00		
**222**	R(100,00)	R(100,00)	**665**			100,00				100,00	
			**666**		100,00				100,00		
			**667**				100,00				100,00
**223**	C(100,00)	C(100,00)	**668**			100,00				100,00	
			**669**				100,00				100,00
			**670**				100,00				100,00
**224**	F(100,00)	F(100,00)	**671**				100,00				100,00
			**672**				100,00				100,00
			**673**			100,00				100,00	
**225**	D(100,00)	D(100,00)	**674**	100,00				100,00			
			**675**		100,00				100,00		
			**676**				100,00		0,29		99,71
**226**	S(100,00)	P(0,29) S(99,71)	**677**		100,00				100,00		
			**678**	100,00				100,00			
			**679**	100,00				100,00			
**227**	T(100,00)	T(100,00)	**680**		100,00				100,00		
			**681**			100,00				100,00	
			**682**			100,00				100,00	
**228**	V(100,00)	V(100,00)	**683**				100,00				100,00
			**684**		100,00				100,00		
			**685**	100,00				100,00			
**229**	T(100,00)	T(100,00)	**686**		100,00				100,00		
			**687**		93,97		6,03		100,00		
			**688**			100,00				100,00	
**230**	E(100,00)	E(100,00)	**689**	100,00				100,00			
			**690**			100,00				100,00	
			**691**	100,00				100,00			
**231**	N(6,03)<br>S(93,97)	S(100,00)	**692**	6,03		93,97				100,00	
			**693**		94,95		5,05		100,00		
			**694**			100,00				100,00	
**232**	D(100,00)	D(100,00)	**695**	100,00				100,00			
			**696**		92,85		7,15		100,00		
			**697**	100,00				100,00			
**233**	I(100,00)	I(100,00)	**698**				100,00				100,00
			**699**		100,00				100,00		
			**700**		100,00				100,00		
**234**	R(100,00)	R(100,00)	**701**			100,00				100,00	
			**702**				100,00				100,00
			**703**			100,00				100,00	
**235**	V(100,00)	V(100,00)	**704**				100,00				100,00
			**705**				100,00				100,00
			**706**			100,00				100,00	
**236**	E(100,00)	E(100,00)	**707**	100,00				100,00			
			**708**			100,00				100,00	
			**709**			100,00				100,00	
**237**	E(100,00)	E(100,00)	**710**	100,00				100,00			
			**711**			100,00				100,00	
**238**	S(100,00)	S(100,00)	**712**				100,00				100,00
			**713**		100,00				100,00		

			**714**	100,00				100,00			
			**715**	100,00				100,00			
**239**	I(100,00)	I(100,00)	**716**				100,00				100,00
			**717**		3,23		96,77				100,00
			**718**				100,00				100,00
**240**	Y(100,00)	C(0,21) Y(99,79)	**719**	100,00				99,79		0,21	
			**720**		100,00				100,00		
			**721**		100,00				100,00		
**241**	Q(100,00)	Q(100,00)	**722**	100,00				100,00			
			**723**	99,02		0,98		100,00			
			**724**				100,00				100,00
**242**	C(100,00)	C(100,00)	**725**			100,00				100,00	
			**726**				100,00				100,00
			**727**				100,00				100,00
**243**	C(100,00)	C(100,00)	**728**			100,00				100,00	
			**729**				100,00				100,00
			**730**			100,00				100,00	
**244**	D(100,00)	D(100,00)	**731**	100,00				100,00			
			**732**		100,00				100,00		
			**733**				100,00				100,00
**245**	L(100,00)	L(100,00)	**734**				100,00				100,00
			**735**			100,00				100,00	
			**736**			100,00				100,00	
**246**	A(100,00)	A(99,79) V(0,21)	**737**		100,00				99,79		0,21
			**738**		100,00				100,00		
			**739**		100,00				100,00		
**247**	P(100,00)	P(100,00)	**740**		100,00				100,00		
			**741**		100,00				100,00		
			**742**			100,00				100,00	
**248**	E(100,00)	E(100,00)	**743**	100,00				100,00			
			**744**	100,00				100,00			
			**745**			100,00				100,00	
**249**	A(100,00)	A(100,00)	**746**		100,00				100,00		
			**747**		100,00				100,00		
			**748**	100,00				100,00			
**250**	R(100,00)	R(100,00)	**749**			100,00				100,00	
			**750**	99,02		0,98		99,51		0,49	
			**751**		100,00				100,00		
**251**	Q(100,00)	Q(100,00)	**752**	100,00				100,00			
			**753**			100,00				100,00	
			**754**			100,00				100,00	
**252**	V(100,00)	V(100,00)	**755**				100,00				100,00
			**756**		98,74		1,26		100,00		
			**757**	100,00				100,00			
**253**	I(100,00)	I(100,00)	**758**				100,00				100,00
			**759**	100,00				100,00			
			**760**	100,00				100,00			
**254**	K(100,00)	K(100,00)	**761**	100,00				100,00			
			**762**			100,00				100,00	
			**763**			100,00				100,00	
**255**	A(100,00)	A(100,00)	**764**		100,00				100,00		
			**765**			100,00				100,00	
			**766**		100,00				100,00		
**256**	L(100,00)	L(100,00)	**767**				100,00				100,00
			**768**		100,00				100,00		
			**769**	100,00				100,00			
**257**	T(100,00)	T(100,00)	**770**		100,00				100,00		
			**771**	100,00				100,00			
			**772**			100,00				100,00	
**258**	E(100,00)	E(100,00)	**773**	100,00				100,00			
			**774**			100,00		6,14		93,86	
**259**	R(100,00)	R(100,00)	**775**		100,00				100,00		

			**776**			100,00				100,00	
			**777**			100,00				100,00	
			**778**		100,00				100,00		
**260**	L(100,00)	L(100,00)	**779**				100,00				100,00
			**780**		100,00				99,34	0,66	
			**781**				100,00				100,00
**261**	Y(100,00)	Y(100,00)	**782**	100,00				100,00			
			**783**				100,00				100,00
			**784**	100,00				100,00			
**262**	I(100,00)	I(100,00)	**785**				100,00				100,00
			**786**		100,00				100,00		
			**787**			100,00				100,00	
**263**	G(100,00)	G(100,00)	**788**			100,00				100,00	
			**789**			100,00				100,00	
			**790**			100,00				100,00	
**264**	G(100,00)	G(100,00)	**791**			100,00				100,00	
			**792**		100,00				100,00		
			**793**		100,00				100,00		
**265**	P(100,00)	P(100,00)	**794**		100,00				100,00		
			**795**		100,00				100,00		
			**796**				100,00				100,00
**266**	L(100,00)	L(100,00)	**797**				100,00				100,00
			**798**	14,45		85,55				100,00	
			**799**	98,74		1,26		99,05		0,95	
**267**	A(1,26) T(98,74)	A(0,95) T(99,5)	**800**		100,00				100,00		
			**801**		100,00				100,00		
			**802**	100,00				100,00			
**268**	N(100,00)	N(100,00)	**803**	100,00				100,00			
			**804**				100,00		0,21		99,79
			**805**				100,00				100,00
**269**	S(100,00)	S(100,00)	**806**		100,00				100,00		
			**807**	100,00				100,00			
			**808**	100,00				100,00			
**270**	K(100,00)	K(100,00)	**809**	100,00				100,00			
			**810**	100,00				100,00			
			**811**			100,00				100,00	
**271**	G(100,00)	G(100,00)	**812**			100,00				100,00	
			**813**			100,00				100,00	
			**814**		100,00				100,00		
**272**	Q(100,00)	Q(100,00)	**815**	100,00				100,00			
			**816**			100,00				100,00	
			**817**	100,00				100,00			
**273**	N(100,00)	N(100,00)	**818**	100,00				100,00			
			**819**		100,00				100,00		
			**820**				100,00				100,00
**274**	C(100,00)	C(100,00)	**821**			100,00				100,00	
			**822**		100,00				100,00		
			**823**			100,00				100,00	
**275**	G(100,00)	G(100,00)	**824**			100,00				100,00	
			**825**		83,87		16,13		100,00		
			**826**				100,00				100,00
**276**	Y(100,00)	Y(100,00)	**827**	100,00				100,00			
			**828**				100,00				100,00
			**829**		100,00				100,00		
**277**	R(100,00)	R(100,00)	**830**			100,00				100,00	
			**831**		100,00				100,00		
			**832**		100,00				100,00		
**278**	R(100,00)	R(100,00)	**833**			100,00				100,00	
			**834**			100,00				100,00	
			**835**				100,00				100,00
**279**	C(100,00)	C(100,00)	**836**			100,00				100,00	
			**837**		100,00				100,00		

			**838**		100,00				100,00		
**280**	R(100,00)	R(100,00)	**839**			100,00				100,00	
			**840**		100,00				98,39		1,61
			**841**			100,00				100,00	
**281**	A(100)	A(100)	**842**		100,00				100,00		
			**843**	1,26		98,74				100,00	
			**844**	100,00				100,00			
**282**	S(100,00)	S(100,00)	**845**			100,00				100,00	
			**846**		100,00				99,75		0,25
			**847**			100,00				100,00	
**283**	G(100,00)	G(100,00)	**848**			100,00				100,00	
			**849**		99,30		0,70		100,00		
			**850**			100,00				100,00	
**284**	V(100,00)	V(100,00)	**851**				100,00				100,00
			**852**			100,00				100,00	
			**853**		100,00				100,00		
**285**	L(100,00)	L(100,00)	**854**				100,00				100,00
			**855**			100,00				100,00	
			**856**	100,00				100,00			
**286**	T(100,00)	T(100,00)	**857**		100,00				100,00		
			**858**			100,00				100,00	
			**859**	100,00				100,00			
**287**	T(100,00)	T(100,00)	**860**		100,00				100,00		
			**861**		99,30		0,70		100,00		
			**862**	100,00				100,00			
**288**	S(100,00)	S(100,00)	**863**			100,00				100,00	
			**864**		100,00				100,00		
			**865**				100,00				100,00
**289**	C(100,00)	C(100,00)	**866**			100,00				100,00	
			**867**		100,00				100,00		
			**868**			100,00				100,00	
**290**	G(100,00)	G(100,00)	**869**			100,00				100,00	
			**870**		3,37		96,63				100,00
			**871**	100,00				100,00			
**291**	N(100,00)	N(100,00)	**872**	100,00				100,00			
			**873**				100,00				100,00
			**874**	100,00				100,00			
**292**	T(100,00)	T(100,00)	**875**		100,00				100,00		
			**876**		100,00				100,00		
			**877**		100,00				100,00		
**293**	L(100,00)	L(100,00)	**878**				100,00				100,00
			**879**		100,00				100,00		
			**880**	100,00				100,00			
**294**	T(100,00)	T(100,00)	**881**		100,00				100,00		
			**882**		100,00				100,00		
			**883**				100,00				100,00
**295**	C(100,00)	C(100,00)	**884**			100,00				100,00	
			**885**		0,98		99,02				100,00
			**886**				100,00				100,00
**296**	Y(100,00)	Y(100,00)	**887**	100,00				100,00			
			**888**		100,00						100,00
			**889**				100,00				100,00
**297**	L(100,00)	L(100,00)	**890**				100,00				100,00
			**891**	100,00				100,00			
			**892**	100,00				100,00			
**298**	K(100,00)	K(100,00)	**893**	100,00				100,00			
			**894**			100,00				100,00	
			**895**			100,00				100,00	
**299**	A(100,00)	A(100,00)	**896**		100,00				100,00		
			**897**		100,00				100,00		
**300**	S(100,00)	S(100,00)	**898**				100,00				100,00
			**899**		100,00				100,00		

			**900**				100,00		0,45		99,55
			**901**			100,00				100,00	
**301**	A(100,00)	A(99,18) V(0,82)	**902**		100,00				99,18		0,82
			**903**	0,84		99,16				100,00	
			**904**			100,00				100,00	
**302**	A(100,00)	A(100,00)	**905**		100,00				100,00		
			**906**		100,00				100,00		
			**907**				100,00				100,00
**303**	C(100,00)	C(100,00)	**908**			100,00				100,00	
			**909**				100,00		100,00		
			**910**		100,00				100,00		
**304**	R(100,00)	R(100,00)	**911**			100,00				100,00	
			**912**	100,00				100,00			
			**913**			100,00				100,00	
**305**	A(100,00)	A(100,00)	**914**		100,00				100,00		
			**915**				100,00		0,37		99,63
			**916**			100,00				100,00	
**306**	A(100,00)	A(100,00)	**917**		100,00				100,00		
			**918**	78,26		21,74		100,00			
			**919**	100,00				100,00			
**307**	K(100,00)	K(100,00)	**920**	100,00				100,00			
			**921**			100,00				100,00	
			**922**		100,00				100,00		
**308**	L(100,00)	L(100,00)	**923**				100,00				100,00
			**924**		100,00				100,00		
			**925**		100,00				100,00		
**309**	Q(100,00)	Q(100,00)	**926**	100,00				100,00			
			**927**			100,00				100,00	
			**928**			100,00				100,00	
**310**	D(100,00)	D(100,00)	**929**	100,00				100,00			
			**930**		100,00				100,00		
			**931**				100,00		0,33		99,67
**311**	C(100,00)	R(0,33) C(99,67)	**932**			100,00				100,00	
			**933**		100,00				100,00		
			**934**	100,00				100,00			
**312**	T(100,00)	T(100,00)	**935**		100,00				100,00		
			**936**	100,00				100,00			
			**937**	100,00				100,00			
**313**	M(100,00)	M(100,00)	**938**				100,00				100,00
			**939**			100,00				100,00	
			**940**		100,00				100,00		
**314**	L(100,00)	L(100,00)	**941**				100,00				100,00
			**942**		100,00				100,00		
			**943**			100,00				100,00	
**315**	V(100,00)	V(100,00)	**944**				100,00				100,00
			**945**			100,00				100,00	
			**946**				100,00				100,00
**316**	C(100,00)	C(100,00)	**947**			100,00				100,00	
			**948**		100,00				100,00		
			**949**			100,00				100,00	
**317**	G(100,00)	G(100,00)	**950**			100,00				100,00	
			**951**			100,00				100,00	
			**952**			100,00				100,00	
**318**	D(100,00)	D(100,00)	**953**	100,00				100,00			
			**954**		100,00				100,00		
			**955**			100,00				100,00	
**319**	D(100,00)	D(100,00)	**956**	100,00				100,00			
			**957**		100,00				100,00		
			**958**		100,00				100,00		
**320**	L(100,00)	L(100,00)	**959**				100,00				100,00
			**960**		0,70		99,30		100,00		
**321**	V(100,00)	V(100,00)	**961**			100,00				100,00	

			**962**				100,00				100,00
			**963**		100,00				100,00		
			**964**			100,00				100,00	
**322**	V(100,00)	V(100,00)	**965**				100,00				100,00
			**966**				100,00				100,00
			**967**	100,00				100,00			
**323**	I(100,00)	I(100,00)	**968**				100,00				100,00
			**969**		100,00				100,00		
			**970**				100,00				100,00
**324**	C(100,00)	C(100,00)	**971**			100,00				100,00	
			**972**				100,00		0,66		99,34
			**973**			100,00				100,00	
**325**	E(100,00)	E(100,00)	**974**	100,00				100,00			
			**975**	100,00				100,00			
			**976**	100,00				100,00			
**326**	S(100,00)	S(100,00)	**977**			100,00				100,00	
			**978**		92,99		7,01		100,00		
			**979**			100,00				100,00	
**327**	A(100,00)	A(100,00)	**980**		100,00				100,00		
			**981**		10,24		89,76				100,00
			**982**			100,00				100,00	
**328**	G(100,00)	G(100,00)	**983**			100,00				100,00	
			**984**			100,00				100,00	
			**985**	100,00				100,00			
**329**	T(100,00)	T(100,00)	**986**		100,00				100,00		
			**987**		100,00				100,00		
			**988**		100,00				100,00		
**330**	Q(100,00)	Q(100,00)	**989**	100,00				100,00			
			**990**			100,00				100,00	
			**991**			100,00				100,00	
**331**	E(100,00)	E(100,00)	**992**	100,00				100,00			
			**993**			100,00				100,00	
			**994**			100,00				100,00	
**332**	D(100,00)	D(100,00)	**995**	100,00				100,00			
			**996**		100,00				100,00		
			**997**			100,00				100,00	
**333**	A(100,00)	A(100,00)	**998**		100,00				100,00		
			**999**			100,00				100,00	
			**1000**			100,00				100,00	
**334**	A(100,00)	A(100,00)	**1001**		100,00				100,00		
			**1002**	1,40		98,60				100,00	
