# Supplementary material for: Late Relapse and Reinfection in HCV Patients Treated with Direct-Acting Antiviral (DAA) Drugs
Source: Viruses. 2021 Jun 16;13(6):1151. doi: 10.3390/v13061151 (PMC8235384; doi:10.3390/v13061151)
Supplement: Supplementary file 1 [file viruses-13-01151-s001.zip › Minosse et al_Table S5.pdf]

Table S5. Frequency of substitutions in Pt5 with respect to HCV GT1a (EF407457.1) or GT1b reference sequences (EU781827.1). The relevant amino acid and nucleotide changes between T0 and T1 are written in red.

Pt5			Pt5										
NS5B aa position	T0 (1a)	T1 (1b)	NS5B nt position	T0 (1a) A	T0 (1a) C	T0 (1a) G	T0 (1a) T	T0 (1a) -	T1 (1b) A	T1 (1b) C	T1 (1b) G	T1 (1b) T	T1 (1b) -
			535			100,00					100,00		
179	A(0,18) V(99,82)	V(100,00)	536		0,18		99,82					100,00	
			537		100,00					100,00			
			538	99,60		0,40						100,00	
180	G(0,40) S(99,60)	S(100,00)	539			100,00				100,00			
			540		100,00					100,00			
			541	99,97		0,03			100,00				
181	E(0,03) K(99,97)	T(100,00)	542	100,00						100,00			
			543			100,00				100,00			
			544		100,00					100,00			
182	L(100,00)	L(100,00)	545				100,00					100,00	
			546		100,00							100,00	
			547		100,00					100,00			
183	P(100,00)	P(100,00)	548		100,00					100,00			
			549				100,00					100,00	
			550			100,00				100,00			
184	V(100,00)	Q(100,00)	551				100,00		100,00				
			552			100,00					100,00		
			553			100,00			0,07		99,93		
185	A(100,00)	A(99,93) T(0,07)	554		100,00					100,00			
			555		100,00					100,00			
			556			100,00					100,00		
186	A(0,20) V(99,80)	A(0,13) V(99,87)	557		0,20		99,80			0,13		99,87	
			558			100,00					100,00		
			559	100,00					99,86		0,14		
187	M(100,00)	M(99,86) V(0,14)	560				100,00					100,00	
			561			100,00					100,00		
			562			100,00			0,05		99,95		
188	G(100,00)	G(99,95) S(0,05)	563			100,00					100,00		
			564	100,00						100,00			
			565	100,00								100,00	
189	S(100,00)	S(100,00)	566			100,00				100,00			
			567		100,00					100,00			
			568				100,00				100,00		
190	S(100,00)	A(100,00)	569		100,00					100,00			
			570		100,00				99,89		0,11		
			571				100,00					100,00	
191	C(0,08) Y(99,92)	C(0,11) Y(99,89)	572	99,92		0,08			99,89		0,11		
			573		100,00					99,95		0,05	
			574			100,00			0,05		99,95		
192	G(100,00)	R(0,05) G(99,95)	575			100,00					100,00		
			576	100,00					100,00				
			577				100,00					100,00	
193	F(100,00)	F(100,00)	578				100,00					100,00	
			579		100,00							100,00	
			580		100,00					100,00			
194	Q(100,00)	R(0,16) Q(99,84)	581	100,00					99,84		0,16		
			582	100,00							100,00		
			583				100,00			0,11		99,89	
195	Y(100,00)	C(0,06) H(0,11) Y(99,83)	584	100,00					99,94		0,06		
			585		100,00					100,00			
196	S(99,97) T(0,03)	S(100,00)	586	0,03			99,97					100,00	
			587		100,00					100,00			

			**588**	100,00							100,00
			**589**		99,97		0,03		100,00		
**197**	P(99,97)<br>S(0,03)	P(100,00)	**590**		100,00				100,00		
			**591**	99,95			0,05				100,00
			**592**			100,00		0,07		99,93	
**198**	G(100,00)	R(0,07)<br>G(99,93)	**593**			100,00				100,00	
			**594**	100,00				99,95		0,05	
			**595**		99,95		0,05		99,95		0,05
**199**	Q(99,95)<br>*(0,05)	R(0,08)<br>Q(99,87)<br>*(0,05)	**596**	100,00				99,91		0,09	
			**597**			100,00				100,00	
			**598**		100,00				99,91		0,09
**200**	R(100,00)	R(99,91)<br>W(0,09)	**599**			100,00				100,00	
			**600**			100,00				100,00	
			**601**			100,00				100,00	
**201**	V(100,00)	A(0,16)<br>V(99,84)	**602**				100,00		0,16		99,84
			**603**		0,15		99,85		100,00		
			**604**			100,00				100,00	
**202**	E(100,00)	E(99,72)<br>G(0,28)	**605**	100,00				99,72		0,28	
			**606**	99,91		0,09				100,00	
			**607**		0,26		99,74		0,24		99,76
**203**	L(0,26)<br>F(99,74)	L(0,24)<br>F(99,76)	**608**				100,00				100,00
			**609**		100,00				100,00		
			**610**		100,00				100,00		
**204**	L(100,00)	L(100,00)	**611**				100,00				100,00
			**612**		100,00			0,07		99,93	
			**613**			100,00				100,00	
**205**	A(0,17)<br>V(99,83)	A(0,11)<br>V(99,89)	**614**		0,17		99,83		0,11		99,89
			**615**			100,00				100,00	
			**616**		99,95		0,05	100,00			
**206**	Q(99,95)<br>*(0,05)	N(99,87)<br>S(0,13)	**617**	100,00				99,87		0,13	
			**618**	100,00					0,08		99,92
			**619**			100,00				100,00	
**207**	A(100,00)	A(100,00)	**620**		100,00				100,00		
			**621**			100,00			100,00		
			**622**		0,13		99,87				100,00
**208**	R(0,13)<br>W(99,87)	W(100,00)	**623**			100,00				100,00	
			**624**			100,00				100,00	
			**625**	100,00				100,00			
**209**	R(0,16)<br>K(99,84)	K(100,00)	**626**	99,84		0,16		100,00			
			**627**	100,00				100,00			
			**628**				100,00				100,00
**210**	S(100,00)	S(100,00)	**629**		100,00				100,00		
			**630**		100,00					100,00	
			**631**	100,00				100,00			
**211**	K(100,00)	K(100,00)	**632**	100,00				100,00			
			**633**			100,00				100,00	
			**634**	100,00				100,00			
**212**	K(100,00)	K(100,00)	**635**	100,00				100,00			
			**636**			100,00		100,00			
			**637**	100,00				100,00			
**213**	T(100,00)	T(100,00)	**638**		100,00				100,00		
			**639**		100,00				100,00		
			**640**		100,00				100,00		
**214**	P(100,00)	P(100,00)	**641**		100,00				100,00		
			**642**			100,00			100,00		
			**643**	100,00				99,94		0,06	
**215**	M(99,87)<br>T(0,13)	M(99,94)<br>V(0,06)	**644**		0,13		99,87				100,00
			**645**			100,00				100,00	
			**646**			100,00				100,00	
**216**	G(100,00)	G(100,00)	**647**			100,00				100,00	
			**648**			100,00			100,00		
**217**	F(100,00)	F(100,00)	**649**				100,00				100,00

			650				100,00				100,00
			651				100,00		100,00		
218	S(100,00)	A(99,94) V(0,06)	652				100,00			100,00	
			653		100,00				99,94		0,06
			654			100,00		0,08		99,92	
219	C(0,09) H(0,09) Y(99,81)	C(0,09) H(0,09)Y(99,82)	655		0,09		99,91		0,09		99,91
			656	99,91		0,09		99,91		0,09	
			657		0,15		99,85		0,16		99,84
220	D(99,86) G(0,14)	D(99,82) G(0,18)	658			100,00				100,00	
			659	99,86		0,14		99,82		0,18	
			660				100,00		100,00		
221	T(100,00)	A(0,10) T(99,90)	661	100,00				99,90		0,10	
			662		100,00				100,00		
			663		100,00				100,00		
222	R(99,91) H(0,09)	R(100,00)	664		100,00				100,00		
			665	0,09		99,91				100,00	
			666		100,00				100,00		
223	C(100,00)	C(99,94) Y(0,06)	667				100,00				100,00
			668			100,00		0,06		99,94	
			669		100,00				0,19		99,81
224	F(100,00)	F(100,00)	670				100,00				100,00
			671				100,00				100,00
			672				100,00				100,00
225	D(99,85) G(0,15)	D(99,83) G(0,17)	673			100,00				100,00	
			674	99,85		0,15		99,83		0,17	
			675		100,00				100,00		
226	S(100,00)	P(0,20) S(99,80)	676				100,00		0,20		99,80
			677		100,00				100,00		
			678		100,00			99,89		0,11	
227	I(0,05) T(99,95)	A(0,11) T(99,89)	679	100,00				99,89		0,11	
			680		99,95		0,05		100,00		
			681	99,88		0,12		100,00			
228	V(100,00)	A(0,19) V(99,81)	682			100,00				100,00	
			683				100,00		0,19		99,81
			684		100,00				100,00		
229	T(100,00)	A(0,16) T(99,84)	685	100,00				99,84		0,16	
			686		100,00				100,00		
			687				100,00		0,11		99,89
230	E(99,63) X(0,37)	E(100,00)	688			99,63				100,00	
			689	99,63				100,00			
			690			100,00				100,00	
231	S(100,00)	N(100,00)	691	100,00				100,00			
			692			100,00		100,00			
			693		100,00				0,20		99,80
232	D(99,83) G(0,17)	D(100,00)	694			100,00				100,00	
			695	99,83		0,17		100,00			
			696		0,04		99,96		0,09		99,91
233	I(100,00)	I(99,88) V(0,12)	697	100,00				99,88		0,12	
			698				100,00				100,00
			699		100,00				100,00		
234	R(99,85) C(0,15)	R(100,00)	700		99,85		0,15		100,00		
			701			100,00				100,00	
			702		0,05		99,95		0,11		99,89
235	M(0,05) T(99,95)	A(0,57) V(99,43)	703	100,00						100,00	
			704		99,95		0,05		0,57		99,43
			705			100,00			0,09		99,91
236	E(100,00)	E(100,00)	706			100,00				100,00	
			707	100,00				100,00			
			708			100,00				100,00	
237	E(100,00)	E(100,00)	709			100,00				100,00	
			710	100,00				100,00			
			711			100,00				100,00	

**238**	A(100,00)	S(100,00)	**712**			100,00					100,00
			**713**		100,00				100,00		
			**714**	100,00				99,95		0,05	
**239**	I(100,00)	I(99,95)<br>T(0,05)	**715**	100,00				100,00			
			**716**				100,00		0,05		99,95
			**717**				100,00		100,00		
**240**	Y(100,00)	N(0,06)<br>C(0,11)<br>H(0,05)<br>Y(99,78)	**718**				100,00	0,06	0,05		99,89
			**719**	100,00				99,89		0,11	
			**720**		100,00				99,94		0,06
**241**	Q(100,00)	Q(100,00)	**721**		100,00				100,00		
			**722**	100,00				100,00			
			**723**	100,00				100,00			
**242**	C(100,00)	R(0,05)<br>C(99,95)	**724**				100,00		0,05		99,95
			**725**			100,00				100,00	
			**726**		0,18		99,82		0,15		99,85
**243**	C(100,00)	R(0,11)<br>C(99,89)	**727**				100,00		0,11		99,89
			**728**			100,00				100,00	
			**729**		0,12		99,88		0,15		99,85
**244**	D(99,71)<br>G(0,29)	D(87,12)<br>G(12,83)<br>V(0,05)	**730**			100,00				100,00	
			**731**	99,71		0,29		87,12		12,83	0,05
			**732**		100,00				100,00		
**245**	L(100,00)	L(99,62)<br>S(0,38)	**733**		100,00						100,00
			**734**				100,00		0,38		99,62
			**735**			100,00		0,08		99,92	
**246**	D(100,00)	A(100,00)	**736**			100,00				100,00	
			**737**	100,00					100,00		
			**738**		100,00				100,00		
**247**	P(100,00)	P(100,00)	**739**		100,00				100,00		
			**740**		100,00				100,00		
			**741**		100,00						100,00
**248**	R(0,14)<br>Q(99,86)	E(100,00)	**742**		100,00					100,00	
			**743**	99,86		0,14		100,00			
			**744**	100,00				100,00			
**249**	A(100,00)	A(99,91)<br>V(0,09)	**745**			100,00				100,00	
			**746**		100,00				99,91		0,09
			**747**		100,00				100,00		
**250**	R(100,00)	R(100,00)	**748**		100,00			100,00			
			**749**			100,00				100,00	
			**750**		100,00			99,93		0,07	
**251**	V(100,00)	Q(100,00)	**751**			100,00			100,00		
			**752**				100,00	100,00			
			**753**	0,06		99,94				100,00	
**252**	A(99,95)<br>V(0,05)	A(99,92)<br>T(0,08)	**754**			100,00		0,08		99,92	
			**755**		99,95		0,05		100,00		
			**756**		100,00				100,00		
**253**	I(99,77)<br>T(0,23)	I(99,75)<br>T(0,11)V(<br>0,14)	**757**	100,00				99,86		0,14	
			**758**		0,23		99,77		0,11		99,89
			**759**		100,00			100,00			
**254**	K(100,00)	R(100,00)	**760**	100,00				100,00			
			**761**	100,00						100,00	
			**762**			100,00		0,06		99,94	
**255**	S(100,00)	P(0,21)<br>S(99,79)	**763**				100,00		0,21		99,79
			**764**		100,00				100,00		
			**765**		100,00			0,46		99,54	
**256**	L(100,00)	H(0,61)<br>L(99,16)<br>X(0,23)	**766**		100,00				99,77		
			**767**				100,00	0,61			99,16
			**768**		100,00				100,00		
**257**	A(0,08)T(99,9<br>2)	A(0,06)<br>I(0,06)<br>T(99,89)	**769**	99,92		0,08		99,94		0,06	
			**770**		100,00				99,94		0,06
			**771**		100,00			99,86			
**258**	E(100,00) K()		**772**			100,00				99,86	

		E(99,86) X(0,14)	**773**	100,00				100,00			
			**774**			100,00				100,00	
**259**	R(100,00)	R(100,00)	**775**	100,00					100,00		
			**776**			100,00				100,00	
			**777**			100,00				100,00	
**260**	L(100,00)	L(100,00)	**778**		100,00				100,00		
			**779**				100,00				100,00
			**780**				100,00				100,00
**261**	C(0,10) Y(99,90)	C(0,08) Y(99,92)	**781**				100,00				100,00
			**782**	99,90		0,10		99,92		0,08	
			**783**				100,00		100,00		
**262**	V(100,00)	I(100,00)	**784**			100,00		100,00			
			**785**				100,00				100,00
			**786**				100,00		100,00		
**263**	G(100,00)	G(100,00)	**787**			100,00				100,00	
			**788**			100,00				100,00	
			**789**			100,00				100,00	
**264**	G(100,00)	G(100,00)	**790**			100,00				100,00	
			**791**			100,00				100,00	
			**792**		95,73						100,00
**265**	L(0,10) P(95,63)	P(100,00)	**793**		95,73				100,00		
			**794**		95,63		0,10		100,00		
			**795**		100,00				100,00		
**266**	L(100,00)	L(100,00)	**796**		100,00				100,00		
			**797**				100,00				100,00
			**798**				100,00			100,00	
**267**	T(100,00)	I(0,06) T(99,94)	**799**	100,00				100,00			
			**800**		100,00				99,94		0,06
			**801**		100,00				100,00		
**268**	N(100,00)	N(99,91) S(0,09)	**802**	100,00				100,00			
			**803**	100,00				99,91		0,09	
			**804**		0,24		99,76		0,15		99,85
**269**	S(100,00)	S(100,00)	**805**				100,00				100,00
			**806**		100,00				100,00		
			**807**	100,00					100,00		
**270**	R(100,00)	K(100,00)	**808**	100,00				100,00			
			**809**			100,00		100,00			
			**810**			100,00		100,00			
**271**	G(100,00)	G(100,00)	**811**			100,00				100,00	
			**812**			100,00				100,00	
			**813**			100,00				100,00	
**272**	E(100,00)	Q(100,00)	**814**			100,00			100,00		
			**815**	100,00				100,00			
			**816**	100,00						100,00	
**273**	N(100,00)	N(99,85) S(0,15)	**817**	100,00				100,00			
			**818**	100,00				99,85		0,15	
			**819**		100,00				100,00		
**274**	C(100,00)	R(0,08) C(99,92)	**820**				100,00		0,08		99,92
			**821**			100,00				100,00	
			**822**		99,93		0,07		99,95		0,05
**275**	G(100,00)	G(100,00)	**823**			100,00				100,00	
			**824**			100,00				100,00	
			**825**		100,00				100,00		
**276**	H(0,09) Y(99,91)	C(0,06) H(0,11) Y(99,84)	**826**		0,09		99,91		0,11		99,89
			**827**	100,00				99,94		0,06	
			**828**				100,00				100,00
**277**	R(100,00)	R(100,00)	**829**		100,00				100,00		
			**830**			100,00				100,00	
			**831**		100,00				100,00		
**278**	R(100,00)	R(99,75) Q(0,10) W(0,15)	**832**	100,00					99,85		0,15
			**833**			100,00		0,10		99,90	
			**834**			100,00				100,00	

			**835**		0,17		99,83		0,05		99,95
**279**	R(0,17) C(99,83)	R(0,05) C(99,95)	**836**			100,00				100,00	
			**837**		100,00				99,95		0,05
			**838**		100,00				100,00		
**280**	R(100,00)	R(100,00)	**839**			100,00				100,00	
			**840**		100,00				100,00		
			**841**	0,14		99,86				100,00	
**281**	A(99,86) T(0,14)	A(100,00)	**842**		100,00				100,00		
			**843**			100,00		100,00			
			**844**	100,00				100,00			
**282**	S(100,00)	S(100,00)	**845**			100,00				100,00	
			**846**		99,95		0,05		99,93		0,07
			**847**			100,00				100,00	
**283**	G(100,00)	G(100,00)	**848**			100,00				100,00	
			**849**		100,00				100,00		
			**850**			100,00				100,00	
**284**	V(100,00)	V(100,00)	**851**				100,00				100,00
			**852**	100,00						100,00	
			**853**		100,00				100,00		
**285**	L(100,00)	L(99,88) P(0,12)	**854**				100,00		0,12		99,88
			**855**			100,00		0,07		99,93	
			**856**	100,00				100,00			
**286**	T(100,00)	M(0,10) T(99,90)	**857**		100,00				99,90		0,10
			**858**			100,00				100,00	
			**859**	100,00				100,00			
**287**	T(100,00)	T(100,00)	**860**		100,00				100,00		
			**861**				100,00		100,00		
			**862**	100,00				99,93		0,07	
**288**	S(100,00)	G(0,07) S(99,93)	**863**			100,00				100,00	
			**864**		99,62		0,38		99,95		0,05
			**865**				100,00		0,11		99,89
**289**	C(100,00)	R(0,11) C(99,89)	**866**			100,00				100,00	
			**867**		0,13		99,87		100,00		
			**868**	0,84		99,16		0,08		99,92	
**290**	G(99,16) S(0,84)	G(99,92) S(0,08)	**869**			100,00				100,00	
			**870**		0,15		99,85		0,07		99,93
			**871**	100,00				99,79		0,21	
**291**	N(100,00)	N(99,71) D(0,21) S(0,08)	**872**	100,00				99,92		0,08	
			**873**		100,00				0,05		99,95
			**874**	99,87		0,13		99,87		0,13	
**292**	A(0,13) T(99,87)	A(0,13) T(99,87)	**875**		100,00				100,00		
			**876**		100,00				100,00		
			**877**		100,00				100,00		
**293**	L(100,00)	L(100,00)	**878**				100,00				100,00
			**879**		100,00				100,00		
			**880**	99,88		0,12		99,86		0,14	
**294**	A(0,12) T(99,88)	A(0,14) T(99,86)	**881**		100,00				100,00		
			**882**				100,00	99,86		0,14	
			**883**		0,12		99,88		0,20		99,80
**295**	R(0,12) C(99,88)	R(0,20) C(99,74) Y(0,06)	**884**			100,00		0,06		99,94	
			**885**		100,00				0,22		99,78
			**886**				100,00		0,09		99,91
**296**	C(0,14) Y(99,86)	C(0,09) H(0,09) Y(99,82)	**887**	99,86		0,14		99,91		0,09	
			**888**		99,95		0,05		100,00		
			**889**	100,00							100,00
**297**	I(100,00)	L(100,00)	**890**				100,00				100,00
			**891**		100,00					100,00	
			**892**	100,00				100,00			
**298**	K(100,00)	K(100,00)	**893**	100,00				100,00			
			**894**			100,00				100,00	
			**895**	0,05		99,95				100,00	
**299**	A(99,95) T(0,05)	A(99,92) V(0,08)	**896**		100,00				99,92		0,08

			**897**		100,00				100,00		
			**898**		100,00			99,82		0,18	
**300**	R(2,62)<br>Q(97,38)	A(0,18)<br>T(99,82)	**899**	97,38		2,62			100,00		
			**900**	100,00					0,20		99,80
			**901**			100,00				100,00	
**301**	A(99,93)<br>V(0,07)	A(99,83)<br>V(0,17)	**902**		99,93		0,07		99,83		0,17
			**903**	100,00				99,71		0,29	
			**904**			100,00				100,00	
**302**	A(99,92)<br>V(0,08)	A(100,00)	**905**		99,92		0,08		100,00		
			**906**		100,00				100,00		
			**907**				100,00				100,00
**303**	C(99,91)<br>Y(0,09)	C(100,00)	**908**	0,09		99,91				100,00	
			**909**				100,00		0,11		99,89
			**910**		100,00				100,00		
**304**	R(99,90)<br>Q(0,10)	R(100,00)	**911**	0,10		99,90				100,00	
			**912**	97,64		2,36		100,00			
			**913**			100,00				100,00	
**305**	A(100,00)	A(100,00)	**914**		100,00				100,00		
			**915**		100,00				0,12		99,88
			**916**			100,00				100,00	
**306**	A(100,00)	A(99,90)<br>V(0,10)	**917**		100,00				99,90		0,10
			**918**	100,00				100,00			
			**919**			100,00		100,00			
**307**	G(100,00)	K(100,00)	**920**			100,00		100,00			
			**921**			100,00				100,00	
			**922**		100,00				100,00		
**308**	L(100,00)	L(100,00)	**923**				100,00				100,00
			**924**		100,00				100,00		
			**925**		100,00				99,95		0,05
**309**	R(100,00)	Q(99,95)<br>*(0,05)	**926**			100,00		100,00			
			**927**			100,00				100,00	
			**928**			100,00				100,00	
**310**	D(100,00)	D(100,00)	**929**	100,00				100,00			
			**930**		98,61		1,39		0,08		99,92
			**931**		0,06		99,94		0,11		99,89
**311**	R(0,06)<br>C(99,94)	R(0,11)<br>C(99,82)<br>Y(0,07)	**932**			100,00		0,07		99,93	
			**933**		100,00				99,95		0,05
			**934**	99,77		0,23		99,91		0,09	
**312**	A(0,23)<br>T(99,77)	A(0,09)<br>T(99,91)	**935**		100,00				100,00		
			**936**		99,96		0,04			100,00	
			**937**	100,00				100,00			
**313**	M(100,00)	I(0,05)<br>M(99,75)<br>T(0,20)	**938**				100,00		0,20		99,80
			**939**			100,00		0,05		99,95	
			**940**		100,00				100,00		
**314**	L(100,00)	L(100,00)	**941**				100,00				100,00
			**942**		99,93		0,07		100,00		
			**943**			100,00				100,00	
**315**	V(100,00)	A(0,10)<br>V(99,90)	**944**				100,00		0,10		99,90
			**945**			100,00		0,05		99,89	
			**946**		0,22		99,78		0,11		99,84
**316**	R(0,22)<br>C(99,72)<br>Y(0,06)	R(0,11)<br>C(99,84)<br>X(0,06)	**947**	0,06		99,94				100,00	
			**948**		0,25		99,75		99,70		0,30
			**949**	0,43		99,57				100,00	
**317**	G(99,57)<br>S(0,43)	G(100,00)	**950**			100,00				100,00	
			**951**		99,91		0,09	100,00			
			**952**			100,00				100,00	
**318**	D(100,00)	D(99,84)<br>G(0,16)	**953**	100,00				99,84		0,16	
			**954**		99,90		0,10		99,93		0,07
			**955**			100,00				100,00	
**319**	D(100,00)	D(100,00)	**956**	100,00				100,00			
			**957**		100,00				100,00		
**320**		L(100,00)	**958**				100,00		100,00		

	L(99,88)S(0,12)		959		0,12		99,88				100,00
			960	100,00					1,49		98,51
321	V(100,00)	A(0,18) V(99,82)	961			100,00				100,00	
			962				100,00		0,18		99,82
			963		100,00				99,95		0,05
322	A(0,12) V(99,88)	A(0,15) V(99,85)	964			100,00				100,00	
			965		0,12		99,88		0,15		99,85
			966				100,00		0,08		99,92
323	I(99,88) V(0,12)	I(99,87) T(0,13)	967	99,88		0,12		100,00			
			968				100,00		0,13		99,87
			969		100,00				99,92		0,08
324	R(0,19) C(99,81) V(0,11)	R(0,18) C(99,77) Y(0,05)	970		0,19		99,81		0,18		99,82
			971			100,00		0,05		99,95	
			972				100,00		0,14		99,86
325	E(99,66) X(0,34)	E(99,44) K(0,11) X(0,46)	973			99,66		0,11		99,44	
			974	99,66				99,54			
			975			100,00				100,00	
326	S(100,00)	S(100,00)	976	100,00				100,00			
			977			100,00				100,00	
			978				100,00		100,00		
327	Q(99,97) L(0,03)	A(97,83) V(2,17)	979		100,00					100,00	
			980	99,97			0,03		97,83		2,17
			981			100,00				100,00	
328	G(100,00)	G(100,00)	982			100,00				100,00	
			983			100,00				100,00	
			984	100,00				100,00			
329	V(100,00)	T(100,00)	985			100,00		100,00			
			986				100,00		100,00		
			987		100,00				99,95		0,05
330	Q(100,00)	R(0,09) Q(99,91)	988		100,00				100,00		
			989	100,00				99,91		0,09	
			990			100,00		100,00			
331	E(100,00)	E(100,00)	991			100,00				100,00	
			992	100,00				100,00			
			993			100,00				100,00	
332	D(100,00)	D(100,00)	994			100,00				100,00	
			995	100,00				100,00			
			996		100,00				100,00		
333	A(99,92) T(0,08)	K(100,00)	997	0,08		99,92		100,00			
			998		100,00			100,00			
			999			100,00				100,00	
334	A(100,00)	A(99,94) T(0,06)	1000			100,00		0,06		99,94	
			1001		100,00				100,00		
			1002			100,00		0,06		99,94	
