# Supplementary material for: Late Relapse and Reinfection in HCV Patients Treated with Direct-Acting Antiviral (DAA) Drugs
Source: Viruses. 2021 Jun 16;13(6):1151. doi: 10.3390/v13061151 (PMC8235384; doi:10.3390/v13061151)
Supplement: Supplementary file 1 [file viruses-13-01151-s001.zip › Minosse et al_Table S6.pdf]

Table S6. Frequency of substitutions in Pt6 with respect to a GT3a reference sequence (X76918.1). The relevant amino acid and nucleotide changes between T0 and T1 are written in red.

NS5B aa position	Pt6 T0 (3a)	Pt6 T1 (3a)	NS5B nt position	Pt6 T0 (3a) A	C	G	T	-	Pt6 T1 (3a) A	C	G	T	-
			535	100,00					100,00				
179	I(100,00)	V(100,00)	536				100,00					100,00	
			537	100,00					100,00				
			538		100,00					100,00			
180	Q(100,00)	Q(100,00)	539	100,00					100,00				
			540			100,00					100,00		
			541	100,00					100,00				
181	K(100,00)	K(100,00)	542	100,00					100,00				
			543			100,00					100,00		
			544				100,00					100,00	
182	L(100,00)	L(100,00)	545				100,00					100,00	
			546			100,00					100,00		
			547				100,00					100,00	
183	S(100,00)	S(100,00)	548		100,00					100,00			
			549	99,80		0,20			100,00				
			550	100,00					100,00				
184	I(99,63) T(0,37)	I(100,00)	551		0,37		99,63					100,00	
			552				100,00					100,00	
			553	0,20		99,80					100,00		
185	A(99,80) T(0,20)	A(100,00)	554		100,00					100,00			
			555			100,00					100,00		
			556			100,00					100,00		
186	A(100,00)	A(100,00)	557		100,00					100,00			
			558			100,00					100,00		
			559	100,00					100,00				
187	M(100,00)	M(100,00)	560				100,00					100,00	
			561			100,00					100,00		
			562			100,00					100,00		
188	G(100,00)	G(100,00)	563			100,00					100,00		
			564				100,00					100,00	
			565				100,00			59,27		40,73	
189	S(100,00)	P(59,27) S(40,73)	566		100,00					100,00			
			567		0,57		99,43					100,00	
			568			100,00					100,00		
190	A(100,00)	A(100,00)	569		100,00					100,00			
			570				100,00					100,00	
			571				100,00					100,00	
191	Y(100,00)	Y(100,00)	572	100,00					100,00				
			573		0,20		99,80					100,00	
			574			100,00					100,00		
192	G(100,00)	G(100,00)	575			100,00					100,00		
			576	100,00					100,00				
			577				100,00					100,00	
193	F(100,00)	F(100,00)	578				100,00					100,00	
			579		100,00					100,00			
			580		100,00					100,00			
194	Q(100,00)	Q(100,00)	581	100,00					100,00				
			582	100,00					100,00				
			583				100,00					100,00	
195	Y(100,00)	Y(100,00)	584	100,00					100,00				
			585		99,63		0,37			100,00			
			586				100,00					100,00	
196	S(100,00)	S(100,00)	587		100,00					100,00			
			588			100,00					100,00		

			**589**		100,00				100,00		
**197**	P(100,00)	P(100,00)	**590**		100,00				100,00		
			**591**				100,00				100,00
			**592**		100,00				100,00		
**198**	Q(100,00)	Q(100,00)	**593**	100,00				100,00			
			**594**	100,00				100,00			
			**595**		100,00				100,00		
**199**	Q(100,00)	Q(100,00)	**596**	100,00				100,00			
			**597**			100,00				100,00	
			**598**		100,00				100,00		
**200**	R(100,00)	R(100,00)	**599**			100,00				100,00	
			**600**			100,00				100,00	
			**601**			100,00				100,00	
**201**	V(100,00)	V(100,00)	**602**				100,00				100,00
			**603**		100,00				100,00		
			**604**			100,00				100,00	
**202**	E(100,00)	E(100,00)	**605**	100,00				100,00			
			**606**	99,67		0,33		100,00			
			**607**		100,00				100,00		
**203**	R(100,00)	R(100,00)	**608**			100,00				100,00	
			**609**				100,00				100,00
			**610**		100,00				100,00		
**204**	L(100,00)	L(100,00)	**611**				100,00				100,00
			**612**	0,29		99,71				100,00	
			**613**		100,00				100,00		
**205**	L(100,00)	L(100,00)	**614**				100,00				100,00
			**615**			100,00		1,34		98,66	
			**616**	98,37	1,63			98,17		1,83	
**206**	Q(1,63) K(98,37)	E(1,83) K(98,17)	**617**	100,00				100,00			
			**618**			100,00				100,00	
			**619**	100,00				100,00			
**207**	M(100,00)	M(100,00)	**620**				100,00				100,00
			**621**			100,00				100,00	
			**622**				100,00				100,00
**208**	W(100,00)	W(100,00)	**623**			100,00				100,00	
			**624**			100,00				100,00	
			**625**	100,00				100,00			
**209**	T(100,00)	T(100,00)	**626**		100,00				100,00		
			**627**		100,00				100,00		
			**628**				100,00				100,00
**210**	S(100,00)	S(100,00)	**629**		100,00				100,00		
			**630**	100,00				100,00			
			**631**	100,00				100,00			
**211**	K(100,00)	K(100,00)	**632**	100,00				100,00			
			**633**			100,00				100,00	
			**634**	100,00				100,00			
**212**	K(100,00)	K(100,00)	**635**	100,00				100,00			
			**636**	100,00				100,00			
			**637**	100,00				100,00			
**213**	T(100,00)	T(100,00)	**638**		100,00				100,00		
			**639**		100,00				100,00		
			**640**		100,00				100,00		
**214**	P(100,00)	P(100,00)	**641**		100,00				100,00		
			**642**		100,00				100,00		
			**643**				100,00				100,00
**215**	L(100,00)	L(100,00)	**644**				100,00				100,00
			**645**			100,00				100,00	
			**646**			100,00				100,00	
**216**	G(100,00)	G(100,00)	**647**			100,00				100,00	
			**648**			100,00				100,00	
**217**	F(100,00)	F(100,00)	**649**				100,00				100,00
			**650**				100,00				100,00

			**651**		100,00				100,00		
			**652**				100,00				100,00
**218**	S(100,00)	S(100,00)	**653**		100,00				100,00		
			**654**	0,24		99,76				100,00	
			**655**				100,00				100,00
**219**	Y(100,00)	Y(100,00)	**656**	100,00				100,00			
			**657**				100,00				100,00
			**658**			100,00				100,00	
**220**	D(100,00)	D(100,00)	**659**	100,00				100,00			
			**660**		100,00				100,00		
			**661**	100,00				100,00			
**221**	T(100,00)	T(100,00)	**662**		100,00				100,00		
			**663**		100,00				100,00		
			**664**		100,00				100,00		
**222**	R(100,00)	R(100,00)	**665**			100,00				100,00	
			**666**		100,00				100,00		
			**667**				100,00				100,00
**223**	C(100,00)	C(100,00)	**668**			100,00				100,00	
			**669**		100,00				100,00		
			**670**				100,00				100,00
**224**	F(100,00)	F(100,00)	**671**				100,00				100,00
			**672**				100,00				100,00
			**673**			100,00				100,00	
**225**	D(100,00)	D(100,00)	**674**	100,00				100,00			
			**675**		100,00				100,00		
			**676**				100,00				100,00
**226**	S(100,00)	S(100,00)	**677**		100,00				100,00		
			**678**	99,43		0,57		100,00			
			**679**	100,00				100,00			
**227**	T(100,00)	T(100,00)	**680**		100,00				100,00		
			**681**		0,20		99,80				100,00
			**682**			100,00				100,00	
**228**	V(100,00)	V(100,00)	**683**				100,00				100,00
			**684**		99,76		0,24		100,00		
			**685**	100,00				100,00			
**229**	T(100,00)	T(100,00)	**686**		100,00				100,00		
			**687**		3,05		96,95				100,00
			**688**			100,00				100,00	
**230**	E(100,00)	E(100,00)	**689**	100,00				100,00			
			**690**	100,00				100,00			
			**691**		100,00				100,00		
**231**	Q(100,00)	Q(100,00)	**692**	100,00				100,00			
			**693**			100,00				100,00	
			**694**			100,00				100,00	
**232**	D(100,00)	D(100,00)	**695**	100,00				100,00			
			**696**		100,00				100,00		
			**697**	100,00				100,00			
**233**	I(100,00)	I(100,00)	**698**				100,00				100,00
			**699**		100,00				100,00		
			**700**	100,00				100,00			
**234**	R(100,00)	R(100,00)	**701**			100,00				100,00	
			**702**			100,00				100,00	
			**703**			100,00				100,00	
**235**	V(100,00)	V(100,00)	**704**				100,00				100,00
			**705**	0,45		99,55				100,00	
			**706**			100,00				100,00	
**236**	E(100,00)	E(100,00)	**707**	100,00				100,00			
			**708**	100,00				100,00			
			**709**			100,00				100,00	
**237**	E(100,00)	E(100,00)	**710**	100,00				100,00			
			**711**			100,00				100,00	
**238**	E(100,00)	E(100,00)	**712**			100,00				100,00	

			**713**	100,00				100,00			
			**714**	99,80		0,20		100,00			
			**715**	100,00				100,00			
**239**	I(100,00)	I(100,00)	**716**				100,00				100,00
			**717**	100,00				100,00			
			**718**				100,00				100,00
**240**	Y(100,00)	Y(100,00)	**719**	100,00				100,00			
			**720**		99,67		0,33		100,00		
			**721**		100,00				100,00		
**241**	Q(100,00)	Q(100,00)	**722**	100,00				100,00			
			**723**	100,00				100,00			
			**724**				100,00				100,00
**242**	C(100,00)	C(100,00)	**725**			100,00				100,00	
			**726**		99,63		0,37		86,94		13,06
			**727**				100,00				100,00
**243**	C(100,00)	C(100,00)	**728**			100,00				100,00	
			**729**		99,55		0,45		96,25		3,75
			**730**	99,59		0,41		100,00			
**244**	N(99,59)<br>D(0,41)	N(100,00)	**731**	100,00				100,00			
			**732**		99,76		0,24		100,00		
			**733**		100,00				100,00		
**245**	L(100,00)	L(100,00)	**734**				100,00				100,00
			**735**		0,20		99,80				100,00
			**736**			100,00				100,00	
**246**	E(100,00)	E(100,00)	**737**	100,00				100,00			
			**738**	99,59		0,41		100,00			
			**739**		100,00				100,00		
**247**	P(100,00)	P(100,00)	**740**		100,00				100,00		
			**741**			100,00				100,00	
			**742**			100,00				100,00	
**248**	E(100,00)	E(100,00)	**743**	100,00				100,00			
			**744**			100,00				100,00	
			**745**			100,00				100,00	
**249**	A(100,00)	A(100,00)	**746**		100,00				100,00		
			**747**		100,00				100,00		
			**748**	100,00				100,00			
**250**	R(100,00)	R(100,00)	**749**			100,00				100,00	
			**750**			100,00				100,00	
			**751**	100,00				100,00			
**251**	K(100,00)	K(100,00)	**752**	100,00				100,00			
			**753**			100,00				100,00	
			**754**			100,00				100,00	
**252**	V(100,00)	V(100,00)	**755**				100,00				100,00
			**756**	3,05		96,95				100,00	
			**757**	100,00				100,00			
**253**	I(100,00)	I(100,00)	**758**				100,00				100,00
			**759**		30,58		69,42		97,98		2,02
			**760**				100,00				100,00
**254**	S(100,00)	S(100,00)	**761**		100,00				100,00		
			**762**		100,00				100,00		
			**763**				100,00				100,00
**255**	S(100,00)	S(100,00)	**764**		100,00				100,00		
			**765**		100,00				100,00		
			**766**		100,00				100,00		
**256**	L(100,00)	L(100,00)	**767**				100,00				100,00
			**768**		100,00				100,00		
			**769**	100,00				100,00			
**257**	T(100,00)	T(100,00)	**770**		100,00				100,00		
			**771**	0,20		99,80				100,00	
			**772**			100,00				100,00	
**258**	E(100,00)	E(100,00)	**773**	100,00				100,00			
			**774**			100,00				100,00	

			**775**		100,00				100,00		
**259**	R(100,00)	R(100,00)	**776**			100,00				100,00	
			**777**			100,00				100,00	
			**778**		100,00				100,00		
**260**	L(100,00)	L(100,00)	**779**				100,00				100,00
			**780**				100,00				100,00
			**781**				100,00				100,00
**261**	Y(100,00)	Y(100,00)	**782**	100,00				100,00			
			**783**		100,00				100,00		
			**784**				100,00				100,00
**262**	C(100,00)	C(100,00)	**785**			100,00				100,00	
			**786**		100,00				100,00		
			**787**			100,00				100,00	
**263**	G(100,00)	G(100,00)	**788**			100,00				100,00	
			**789**	100,00				100,00			
			**790**			100,00				100,00	
**264**	G(100,00)	G(100,00)	**791**			100,00				100,00	
			**792**		100,00				100,00		
			**793**		100,00				100,00		
**265**	P(100,00)	P(100,00)	**794**		100,00				100,00		
			**795**				100,00				100,00
			**796**	100,00				100,00			
**266**	M(100,00)	M(100,00)	**797**				100,00				100,00
			**798**			100,00				100,00	
			**799**				100,00				100,00
**267**	F(100,00)	F(100,00)	**800**				100,00				100,00
			**801**		100,00				100,00		
			**802**	100,00				100,00			
**268**	N(100,00)	N(100,00)	**803**	100,00				100,00			
			**804**		99,47		0,53		100,00		
			**805**	100,00				100,00			
**269**	S(100,00)	S(100,00)	**806**			100,00				100,00	
			**807**		100,00				100,00		
			**808**	100,00				100,00			
**270**	K(100,00)	K(100,00)	**809**	100,00				100,00			
			**810**			100,00				100,00	
			**811**			100,00				100,00	
**271**	G(100,00)	G(100,00)	**812**			100,00				100,00	
			**813**			100,00				100,00	
			**814**			100,00				100,00	
**272**	A(100,00)	A(100,00)	**815**		100,00				100,00		
			**816**		99,71		0,29		100,00		
			**817**		100,00				100,00		
**273**	Q(100,00)	Q(100,00)	**818**	100,00				100,00			
			**819**			100,00				100,00	
			**820**				100,00				100,00
**274**	C(100,00)	C(100,00)	**821**			100,00				100,00	
			**822**		100,00				100,00		
			**823**			100,00				100,00	
**275**	G(100,00)	G(100,00)	**824**			100,00				100,00	
			**825**				100,00				100,00
			**826**				100,00				100,00
**276**	Y(100,00)	Y(100,00)	**827**	100,00				100,00			
			**828**		0,20		99,80				100,00
			**829**		100,00				100,00		
**277**	R(100,00)	R(100,00)	**830**			100,00				100,00	
			**831**		100,00				100,00		
			**832**		100,00				100,00		
**278**	R(100,00)	R(100,00)	**833**			100,00				100,00	
			**834**		100,00				100,00		
**279**	C(100,00)	C(100,00)	**835**				100,00				100,00
			**836**			100,00				100,00	

			**837**		100,00				100,00		
			**838**		100,00				100,00		
**280**	R(100,00)	R(100,00)	**839**			100,00				100,00	
			**840**				100,00				100,00
			**841**			100,00				100,00	
**281**	A(100,00)	A(100,00)	**842**		100,00				100,00		
			**843**		98,90		1,10		100,00		
			**844**	100,00				100,00			
**282**	S(100,00)	S(100,00)	**845**			100,00				100,00	
			**846**				100,00				100,00
			**847**			100,00				100,00	
**283**	G(100,00)	G(100,00)	**848**			100,00				100,00	
			**849**	99,47			0,53	100,00			
			**850**			100,00				100,00	
**284**	V(100,00)	V(100,00)	**851**				100,00				100,00
			**852**				100,00				100,00
			**853**				100,00				100,00
**285**	L(100,00)	L(100,00)	**854**				100,00				100,00
			**855**			100,00				100,00	
			**856**		100,00				100,00		
**286**	P(100,00)	P(100,00)	**857**		100,00				100,00		
			**858**				100,00				100,00
			**859**	100,00				100,00			
**287**	T(100,00)	T(100,00)	**860**		100,00				100,00		
			**861**		99,27		0,73		97,98		2,02
			**862**	100,00				100,00			
**288**	S(100,00)	S(100,00)	**863**			100,00				100,00	
			**864**		100,00				100,00		
			**865**				100,00				100,00
**289**	F(100,00)	F(100,00)	**866**				100,00				100,00
			**867**		100,00				100,00		
			**868**			100,00				100,00	
**290**	G(100,00)	G(100,00)	**869**			100,00				100,00	
			**870**		99,76		0,24		100,00		
			**871**	100,00				100,00			
**291**	N(100,00)	N(100,00)	**872**	100,00				100,00			
			**873**		99,71		0,29		100,00		
			**874**	100,00				100,00			
**292**	T(100,00)	T(100,00)	**875**		100,00				100,00		
			**876**	99,55		0,45		100,00			
			**877**	100,00				100,00			
**293**	I(100,00)	I(100,00)	**878**				100,00				100,00
			**879**		100,00				100,00		
			**880**	100,00				100,00			
**294**	T(100,00)	T(100,00)	**881**		100,00				100,00		
			**882**				100,00				100,00
			**883**				100,00				100,00
**295**	C(100,00)	C(100,00)	**884**			100,00				100,00	
			**885**		0,29		99,71				100,00
			**886**				100,00				100,00
**296**	Y(100,00)	Y(100,00)	**887**	100,00				100,00			
			**888**		100,00				100,00		
			**889**	100,00				100,00			
**297**	I(100,00)	I(100,00)	**890**				100,00				100,00
			**891**		100,00				100,00		
			**892**	100,00				100,00			
**298**	K(100,00)	K(100,00)	**893**	100,00				100,00			
			**894**			100,00				100,00	
			**895**			100,00				100,00	
**299**	A(100,00)	A(100,00)	**896**		100,00				100,00		
			**897**		100,00				100,00		
**300**	T(100,00)	T(100,00)	**898**	100,00				100,00			

			**899**		100,00				100,00		
			**900**	99,67		0,33		100,00			
			**901**			100,00				100,00	
**301**	A(100,00)	A(100,00)	**902**		100,00				100,00		
			**903**			99,76	0,24			100,00	
			**904**			100,00				100,00	
**302**	A(100,00)	A(100,00)	**905**		100,00				100,00		
			**906**				100,00				100,00
			**907**			100,00				100,00	
**303**	A(100,00)	A(100,00)	**908**		100,00				100,00		
			**909**	0,49		99,51				100,00	
			**910**	100,00				100,00			
**304**	R(0,29) K(99,71)	R(89,24) K(10,76)	**911**	99,71		0,29		10,76		89,24	
			**912**			100,00				100,00	
			**913**			100,00				100,00	
**305**	A(100,00)	A(100,00)	**914**		100,00				100,00		
			**915**		99,55		0,45		100,00		
			**916**			100,00				100,00	
**306**	A(100,00)	A(100,00)	**917**		100,00				100,00		
			**918**				100,00				100,00
			**919**			100,00				100,00	
**307**	G(100,00)	G(100,00)	**920**			100,00				100,00	
			**921**				100,00				100,00
			**922**		100,00				100,00		
**308**	L(100,00)	L(100,00)	**923**				100,00				100,00
			**924**		100,00				100,00		
			**925**		100,00				100,00		
**309**	R(100,00)	R(100,00)	**926**			100,00				100,00	
			**927**			100,00				100,00	
			**928**	100,00				100,00			
**310**	N(100,00)	N(100,00)	**929**	100,00				100,00			
			**930**		100,00				100,00		
			**931**		100,00				100,00		
**311**	P(100,00)	P(100,00)	**932**		100,00				100,00		
			**933**	35,87		64,13		85,30		14,70	
			**934**			100,00				100,00	
**312**	D(100,00)	D(100,00)	**935**	100,00				100,00			
			**936**		100,00				100,00		
			**937**				100,00				100,00
**313**	F(100,00)	F(100,00)	**938**				100,00				100,00
			**939**				100,00				100,00
			**940**		100,00				100,00		
**314**	L(100,00)	L(100,00)	**941**				100,00				100,00
			**942**				100,00		1,25		98,75
			**943**			100,00				100,00	
**315**	V(100,00)	V(100,00)	**944**				100,00				100,00
			**945**		100,00				100,00		
			**946**				100,00				100,00
**316**	C(100,00)	C(100,00)	**947**			100,00				100,00	
			**948**		100,00				100,00		
			**949**			100,00				100,00	
**317**	G(100,00)	G(100,00)	**950**			100,00				100,00	
			**951**			100,00				100,00	
			**952**			100,00				100,00	
**318**	D(100,00)	D(100,00)	**953**	100,00				100,00			
			**954**				100,00				100,00
			**955**			100,00				100,00	
**319**	D(100,00)	D(100,00)	**956**	100,00				100,00			
			**957**				100,00				100,00
			**958**		100,00				100,00		
**320**	L(100,00)	L(100,00)	**959**				100,00				100,00
			**960**	6,19		93,81				100,00	

**321**	V(100,00)	V(100,00)	**961**			100,00				100,00	
			**962**				100,00				100,00
			**963**		99,39		0,61		100,00		
**322**	V(100,00)	V(100,00)	**964**			100,00				100,00	
			**965**				100,00				100,00
			**966**			100,00				100,00	
**323**	V(100,00)	V(100,00)	**967**			100,00				100,00	
			**968**				100,00				100,00
			**969**			100,00				100,00	
**324**	A(100,00)	A(100,00)	**970**			100,00				100,00	
			**971**		100,00				100,00		
			**972**				100,00				100,00
**325**	E(99,67)<br>X(0,33)	E(100,00)	**973**			99,67				100,00	
			**974**	99,67				100,00			
			**975**			100,00				100,00	
**326**	S(100,00)	S(100,00)	**976**	100,00				100,00			
			**977**			100,00				100,00	
			**978**		99,76		0,24		100,00		
**327**	D(100,00)	D(100,00)	**979**			100,00				100,00	
			**980**	100,00				100,00			
			**981**		2,04		97,96				100,00
**328**	G(100,00)	G(100,00)	**982**			100,00				100,00	
			**983**			100,00				100,00	
			**984**		100,00				100,00		
**329**	V(100,00)	V(100,00)	**985**			100,00				100,00	
			**986**				100,00				100,00
			**987**				100,00				100,00
**330**	D(100,00)	D(100,00)	**988**			100,00				100,00	
			**989**	100,00				100,00			
			**990**		99,31		0,69		100,00		
**331**	G(100,00)	G(100,00)	**991**			100,00				100,00	
			**992**			100,00				100,00	
			**993**			100,00				100,00	
**332**	D(100,00)	D(100,00)	**994**			100,00				100,00	
			**995**	100,00				100,00			
			**996**				100,00				100,00
**333**	R(100,00)	R(100,00)	**997**	100,00				100,00			
			**998**			100,00				100,00	
			**999**	100,00				100,00			
**334**	A(100,00)	A(100,00)	**1000**			100,00				100,00	
			**1001**		100,00				100,00		
			**1002**	97,11		2,89		100,00			
