# Supplementary figures and images for: Late Relapse and Reinfection in HCV Patients Treated with Direct-Acting Antiviral (DAA) Drugs
Source: Viruses. 2021 Jun 16;13(6):1151. doi: 10.3390/v13061151 (PMC8235384; doi:10.3390/v13061151)

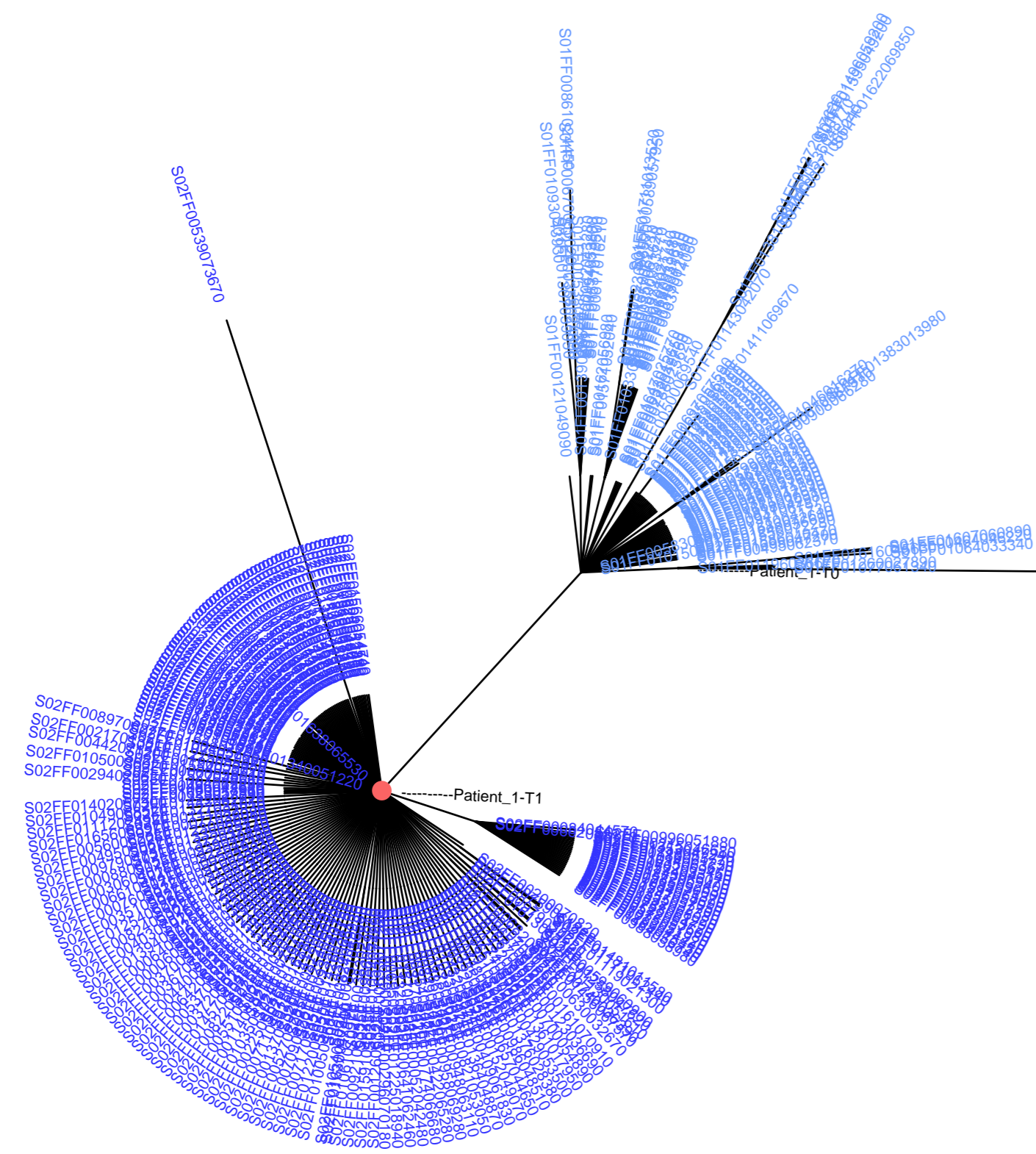

EF407457

Supplement: Supplementary file 1 [file viruses-13-01151-s001.zip › Minosse et al_Figure S1.pdf]

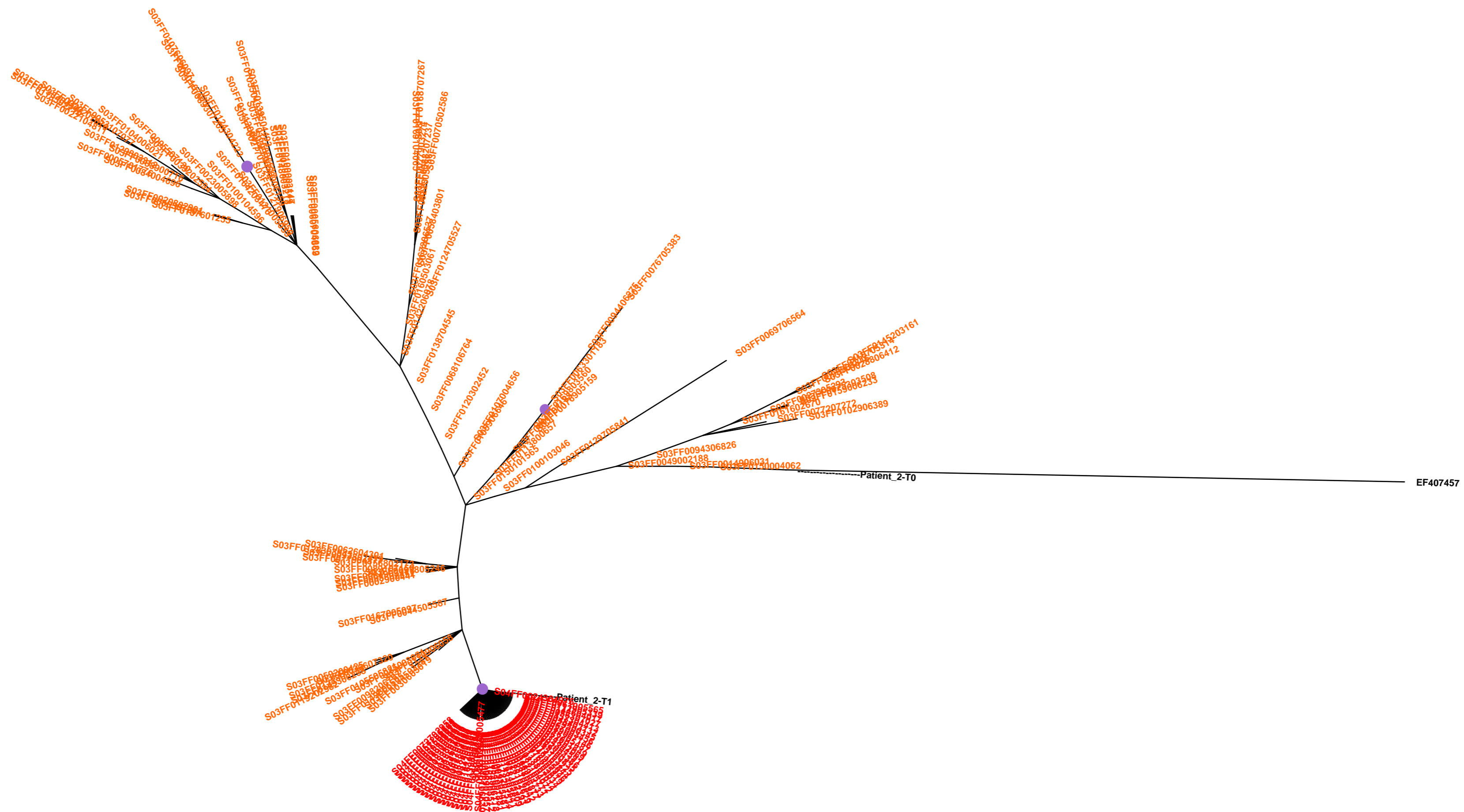

0.007

Supplement: Supplementary file 1 [file viruses-13-01151-s001.zip › Minosse et al_Figure S2.pdf]

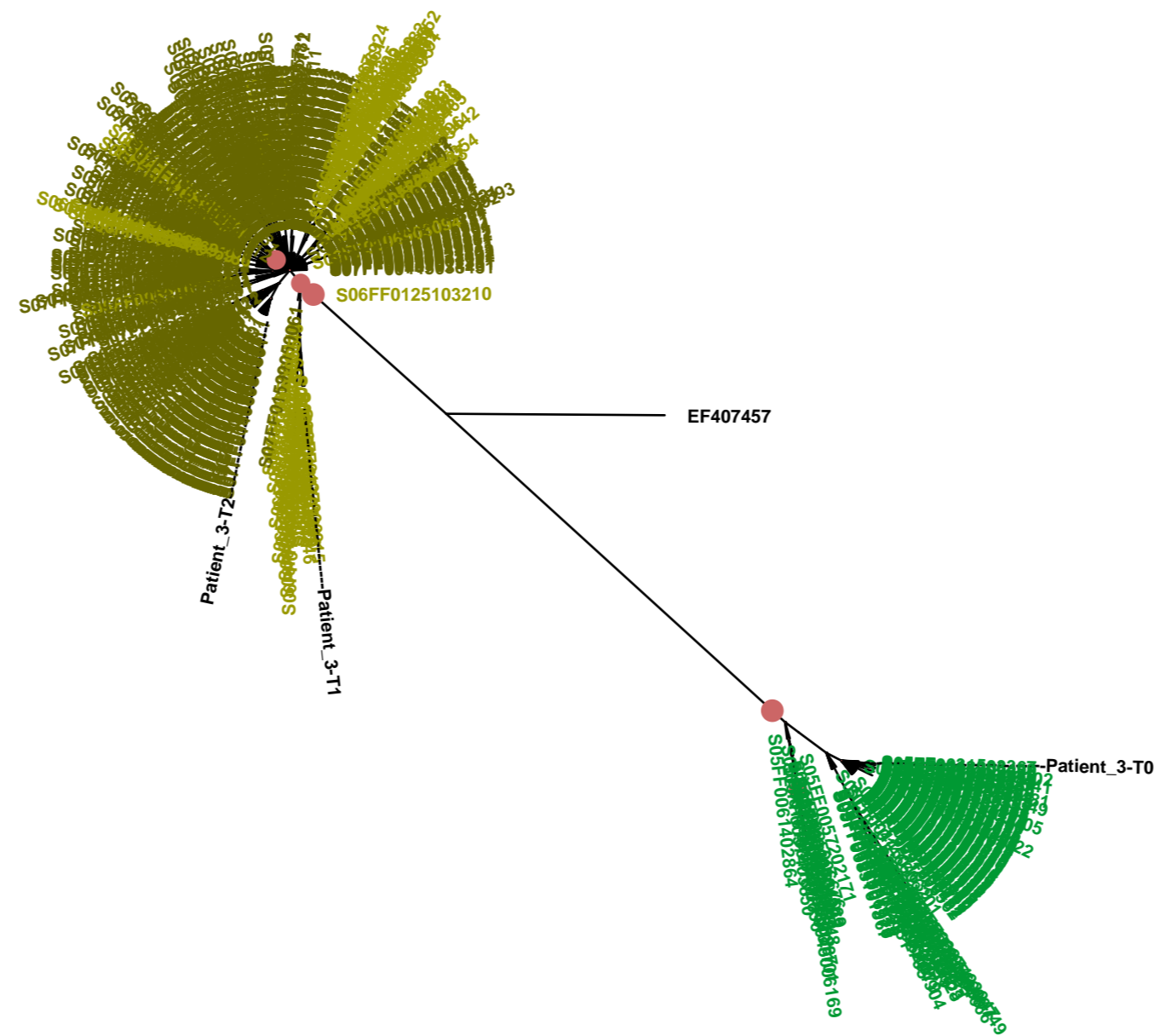

0.009

Supplement: Supplementary file 1 [file viruses-13-01151-s001.zip › Minosse et al_Figure S3.pdf]

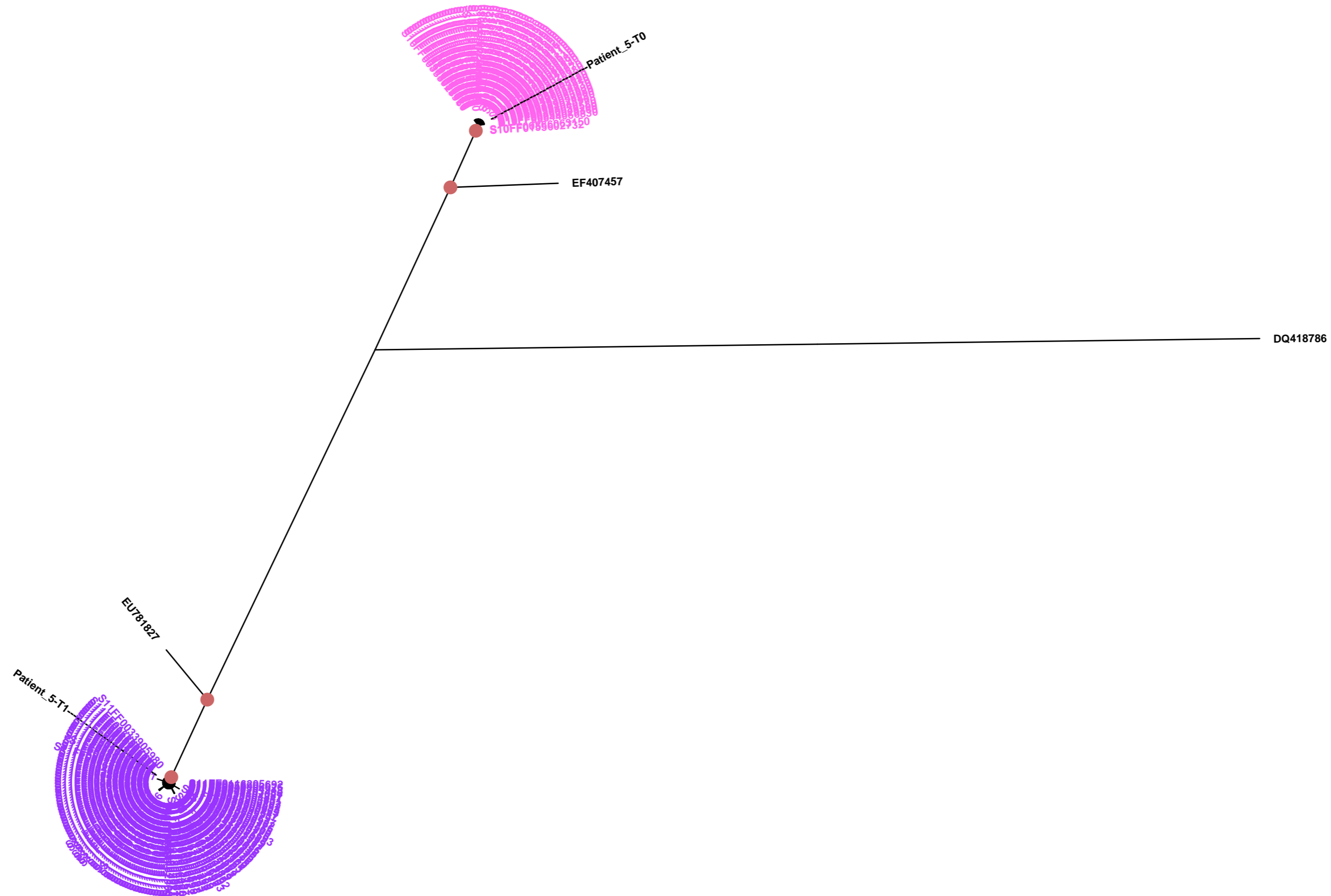

Supplement: Supplementary file 1 [file viruses-13-01151-s001.zip › Minosse et al_Figure S5.pdf]

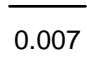

0.007

Supplement: Supplementary file 1 [file viruses-13-01151-s001.zip › Minosse et al_Figure S6.pdf]

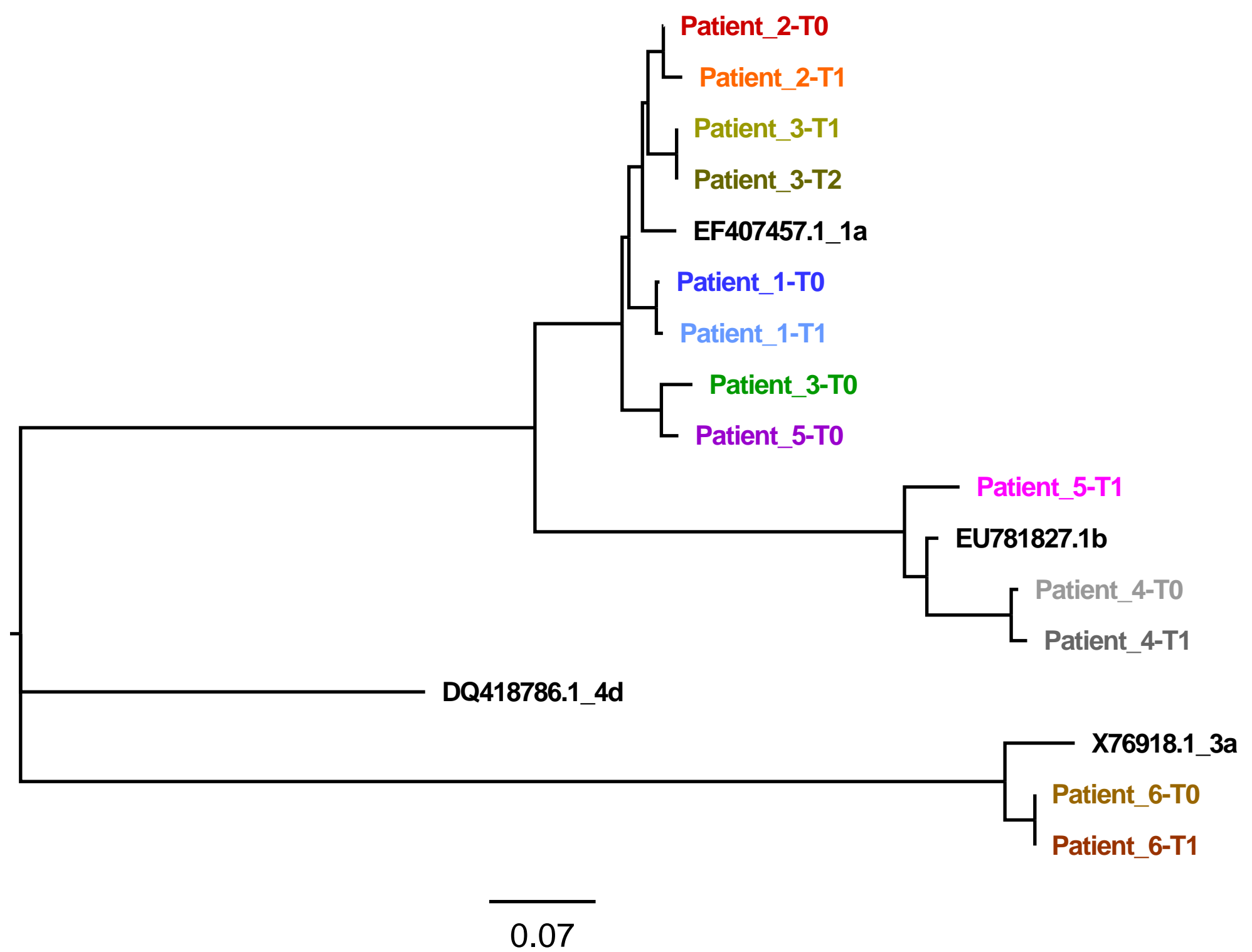

Supplement: Supplementary file 1 [file viruses-13-01151-s001.zip › Minosse et al_Figure S7.pdf]
