# Supplementary material for: Late Relapse and Reinfection in HCV Patients Treated with Direct-Acting Antiviral (DAA) Drugs
Source: Viruses. 2021 Jun 16;13(6):1151. doi: 10.3390/v13061151 (PMC8235384; doi:10.3390/v13061151)
Supplement: Supplementary file 1 [file Minosse et al_Figure S4.pdf › Minosse et al_Figure S4.pdf]

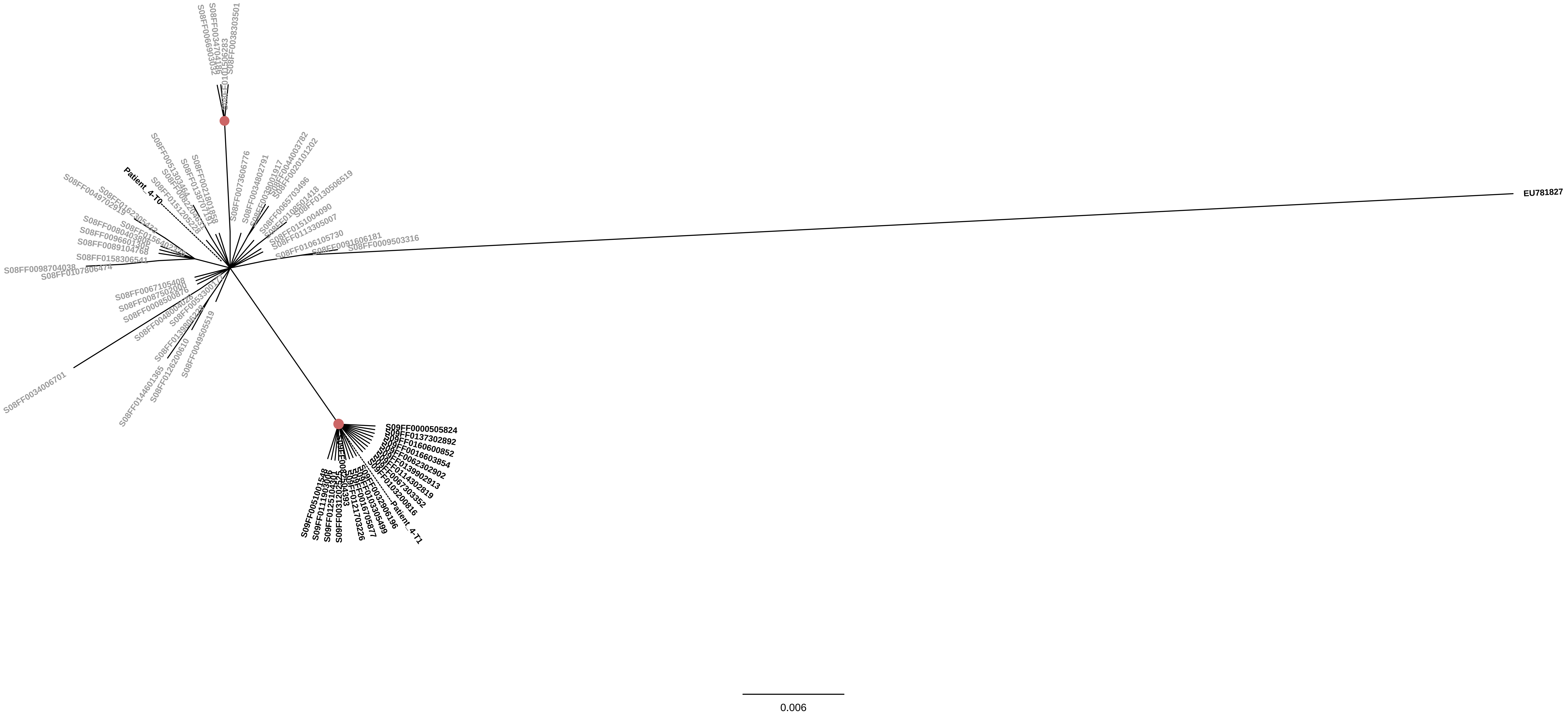

EU781827
S09FF0000505824
S09FF0137302892
S09FF0160600852
S09FF0016603854
S09FF0062302902
S09FF0139902913
Patient_4-T1
Patient_4-T0
S08FF0009503316
S08FF0091606181
S08FF0106105730
S08FF0113305007
S08FF0151004090
S08FF0130506519
S08FF0073606776
S08FF0034802791
S08FF0049702919
S08FF0158306541
S08FF0089104768
S08FF0098704038
S08FF0107806474
S08FF0067105408
S08FF0034006701
0.006
